# Acknowledgment to Reviewers of *Sensors* in 2020

**DOI:** 10.3390/s21030854

**Published:** 2021-01-28

**Authors:** Sensors Editorial Office

**Affiliations:** MDPI AG, St. Alban-Anlage 66, 4052 Basel, Switzerland

Peer review is the driving force of journal development, and reviewers are gatekeepers who ensure that *Sensors* maintains its standards for the high quality of its published papers. Thanks to the cooperation of our reviewers, in 2020, the median time to first decision was 16 days and the median time to publication was 39 days. The editors would like to express their sincere gratitude to the following reviewers for their precious time and dedication, regardless of whether the papers were finally published:
Aalsalem, MohammedLópez Ortega, AlbertoAbad, BegoñaLópez Ramos, Juan AntonioAbah, ColetteLópez, AinaraAbarca-Alvarez, Francisco JavierLópez, AliciaAbascal, JulioLópez, MaríaAbate, GiuliaLópez, Miguel ÁngelAbawajy, JemalLópez-Benitez, MiguelAbbas, TauqeerLopez-De-Teruel, Pedro E.Abbasi, QammerLópez-Estrada, Francisco RonayAbbasi, UbaidLopez-Fernandez, JesusAbbasi, ZahraLópez-Hernández, Luz BereniceAbbasnejad, BehrokhLópez-Huerta, FranciscoAbbaszadeh, ShivaLópez-López, DanielAbbatangelo, MarcoLópez-Mancilla, DidierAbbate, AndreaLopez-Martin, ManuelAbbate, CarmineLópez-Martínez, AlbertoAbbene, LeonardoLópez-Martínez, Jesús AlbertoAbbod, MaysamLopez-Molina, CarlosAbd-Alhameed, RaedLópez-Nava, Irvin HusseinAbdelgawad, KareemLópez-Ochoa, Luis MaríaAbdeljaber, OsamaLópez-Pastor, Jose-AntonioAbdel-Motaleb, Ibrahim M.López-Ruiz, BeatrizAbdel-Nasser, MohamedLópez-Sastre, Roberto JavierAbdi, GhasemLopomo, NicolaAbdikan, SayginLoprencipe, GiuseppeAbdolghafoorian, AbedehLopreside, AntoniaAbdolvand, RezaLoranger, SébastienAbdrabou, AtefLord, SueAbdul Kudus, SakhiahLoredana, DumitrascuAbdul Wahab, OmarLorenzo-Navarro, JavierAbdul, RazaqueLoreti, PierpaoloAbdul-Aziz, AliLorig, FabianAbdulhalim, IbrahimLorincz, JosipAbdulkhaleq, AhmedLorussi, FedericoAbdullah, Oday IbraheemLorusso, MassimoAbdullah, OsamahLorusso, VitoAbdulridha, JaafarLos, SietseAbedini-Nassab, RoozbehŁoś, SzymonAbegão, Luis Miguel GomesLosada-Perez, PatriciaAbel, DirkLosi, ArturoAbenza, Pedro Pablo GarridoLoskot, PavelAbhayasinghe, NimsiriLosson, EtienneAbileah, RonLostado, RubénAbiri, RezaLotoshynska, NataliiaAbolghasemi, VahidLou, ChingwenAboltins, ArtursLou, Der-ChyuanAbonyi, JánosLou, ZhichaoAbosekeen, AshrafLoukatos, DimitriosAbot, JandroLoureiro, PauloAboudan, AlessioLouro, PaulaAbouhussien, AhmedLouvaris, ZafeirisAbou-Saleh, Radwa H.Lova, PaolaAbramović, BornaLovas, TamasAbramovich, HaimLovchik, RobertAbrantes, João Carlos De CastroLoveless, DanielAbrehdary, MajidLovisolo, LisandroAbreu, AntónioLow, EicherAbreu, CarlosLowry, MarkAbrol, AmritLoyola-González, OctavioAbt, GrantLöytönen, MarkkuAbu Ghazaleh, HaithamLozano Guerrero, Antonio JoséAbu Shattal, MohammadLozano Murciego, ÁlvaroAbu-Farsakh, MuradLozano, JesusAbumazwed, AhmedLozano-Diez, AliciaAbu-Zeid, NasserLozano-Galant, Jose AntonioAccoroni, StefanoLozito, Gabriele MariaAcerbi, FabioLu, BoAceto, GiuseppeLu, ChaoAceto, MaurizioLu, Ching-TaAcevedo, AndreaLu, DebiaoAcevedo, Rubén CarlosLu, DingqiangAcharya, Tri DevLu, EnliAcharyya, NirmalenduLu, FeiAchim, CristinaLu, FengAcién, MaribelLu, HaoAciu, Lia E.Lü, HongliangAcken, John M.Lu, HuadongAcuna, EdgarLu, HuimingAdam, GeorgeLu, JianAdam, George K.Lu, JinAdamczuk Oliveira, GilsonLu, JingAdamczyk, MarcinLu, JunpengAdame Vázquez, ToniLu, KelinAdamopoulos, EfstathiosLu, LiAdamopoulou, EvgeniaLu, PanAdams, Alexander TravisLu, PengAdams, Mark L.Lu, QingAdams, MartinLu, ShanAdamy, JürgenLu, ShijianAdão, TelmoLu, ShuangAdcock, ChristopherLu, SiliangAddabbo, PiaLu, WeiAdeagbo, Waheed A.Lu, WenlongAdegoke, ElijahLu, XiaodongAdekunle, Timothy O.Lu, XiaolongAdeloju, SamuelLu, XiaoxuanAdhi Tama, BayuLu, XinAdkins, Kevin ALu, YanAdly, AmrLu, YijiAdolfo Calvo-Cases, AdolfoLu, YuanfuAdornetto, AnnagraziaLu, YubingAdriaens, InesLu, YudongAdrion, FelixLu, ZhemingAfa Michael, InikuroLu, ZhenboAfaqui, Muhammad ShahwaizLubal, PřemyslAfonso, ManyaLuca, Gaetano DeAfshar, Mehdi H.Luca, MariaAfzal, AdeelLuca, RomeoAfzal, Muhammad RaheelLucas, Guilherme B.Agafonov, VadimLucas, PeterAgapiou, AthosLucchi, ElenaAgarwal, AnujLuchian, TudorAgarwal, MangilalLucioli, SimonaAgarwal, RachitLuckner, MarcinAggarwal, GeetikaŁuczak, DominikAggogeri, FrancescoŁuczak, SergiuszAghajani, HalehLuczak, TonyAghakhani, AmirrezaLüdtke, StefanAgiwal, MamtaLudwig, FrankAgocs, EmilLudwig, NicolaAgostini, MatteoLudwinek, KrzysztofÁgoston, KatalinLuger, TessyAgrafiotis, PanagiotisLughofer, EdwinAgranov, GennadiyLugmayr, ArturAgrawal, HimanshuLugo, Jesus EduardoAgrawala, Ashok K.Lugovtsova, YevgeniyaAgüera-Vega, FranciscoLugrís, UrbanoAguiar, AndréLuh, Jer-JunnAguilar-Caballos, María PazLühmann, Alexander VonAguilar-González, AbielLuigi, CoppolinoAguilar-Gonzalez, RafaelLuis Alvarez-Perez, JoseAguilar-Mejía, OmarLuján-Álvarez, ConcepcionAguilera, Cristhian A.Luján-Mora, SergioAguirre-Calderón, OscarLukač, NikoAhad, Atiqur RahmanLukas, VojtěchAhamad, MazbahulLukin, VladimirAhamed, Md MarufLukinac, JasminaAhamed, Nizam UddinLukoševičienė, OnaAhamed, TofaelŁukowski, AdrianAhi, KiarashLullo, GiuseppeAhma, LuanLumentut, Mikail F.Ahmad, AdeelLuna Moreno, DonatoAhmad, AshfaqLuna, Aderval S.Ahmad, AwaisLuna-Lozano, Pablo S.Ahmad, JawadLuna-Perejón, FranciscoAhmad, MuneebLungu, Magdalena ValentinaAhmad, TanveerLuo, BingkunAhmad, WasimLuo, CaiAhmadi, SohaLuo, ChangqingAhmadinia, AliLuo, ChenggaoAhmadivand, ArashLuo, FulinAhmadpour, NaseemLuo, HaiyongAhmed, FaridLuo, HanjiangAhmed, MohiuddinLuo, LihuiAhmedt-Aristizabal, DavidLuo, MingxingAhn, HosangLuo, NingqiAhn, SangdooLuo, QuantianAhn, SoonjaeLuo, ShaojuanAhrberg, Christian D.Luo, XiangangAhsani, VesalLuo, YangAhshan, RazzaqulLuo, YanhuaAhuett Garza, HoracioLuo, YiAhuir-Torres, JuanLuo, YiranAi, HaojunLuo, YutaoAi, WeiweiLuo, ZhiyuanAiello, GiuseppeLuong, HiepAiello, OrazioLuongo, OrlandoAizpurua, Jose IgnacioLupi, CyrilAjay, AshokLupu, CiprianAjith Singh, Mithun KuniyilLupu, EugenAkansel, CosgunLuque, AmaliaAkasiadis, CharilaosLuque, AntonioAkbar, SheikhLuque, JoaquínAkbas, MustafaLuque-Nieto, Miguel AngelAkgun, Omer CanLuque-Nieto, Miguel-AngelAkimoto, TakumaLuque-Sendra, AmaliaAkimov, SergeiŁusakowski, JerzyAkinsolu, Mobayode O.Lussier, BenjaminAkitsu, TakashiroLuštrek, MitjaAkiyama, IwakiLuteberget, Live SteinnesAkiyama, YasuhiroLuttgen, AndreaAkjouj, AbdellatifLuviano-Cruz, DavidAkkaynak, DeryaLuviano-Juárez, AlbertoAkoğlu, GozeLuzuriaga, Jorge E.Akram, Muhammad NadeemLv, ChenAksac, AlperLv, HaoAkşit, KaanLv, NengchaoAksjonov, AndreiLv, ZhiyongAktas, EmelLvova, LarisaAkturk, AkinLy, Ngoc Q.Akyildiz, Ian F.Lyakhov, PavelAl Sadsoon, MohammedLydon, MyraAl Zoubi, ObadaLynn Speiser, JaimeAl_Amri, MohammadLyons, AshleyAla, GuidoLytridis, ChrisAlaggio, RoccoLytvynenko, VladimirAlahi, Md Eshrat E.Lyzenga, DavidAlam, Arif UlMa, BingpengAlam, FakhrulMa, ChangxiAlam, Miah Md AshrafulMa, ChenAlam, Muhamamd MahtabMa, ChunfengAlam, MuhammadMa, CongcongAlam, TauhidulMa, DanAlamaniotis, MiltiadisMa, DaolinAlander, JarmoMa, GuanyiAlanis, Alma Y.Ma, Guo-mingAlavi, Amir H.Ma, HanbinAlbajez, Jose AntonioMa, HongyangAlbarracín, LluísMa, JiajuAlbarracín, RicardoMa, JianhuaAlbéri, MatteoMa, JiayiAlberini, FedericoMa, LiuhaoAlbert, JustinMa, MengchaoAlbert, Wayne J.Ma, MingAlberti, LuigiMa, MingguoAlbertin, FauziaMa, MingshengAlbertini, Bruno De CarvalhoMa, ShuangAlberto, NéliaMa, TinghuaiAlbiol, AlbertoMa, WeiAlbu, FelixMa, WeiqiangAlbu, MihaelaMa, XiaolinAlbuquerque, Daniel FilipeMa, XignpoAlburquerque-Sendín, FranciscoMa, XinAlcaraz, CristinaMa, YanAlcarria, RamonMa, Yi-deAldabaldetreku, GotzonMa, YingyingAldaya, IvanMa, YinjiAldea, EmanuelMa, YufeiAldewachi, HasanMa, YuliangAldini, AlessandroMa, ZhenliangAldrigo, MartinoMaarouf, AhmedAleinov, IgorMaarten, GrachtenAleixandre, ManuelMaas, Hans-GerdAlejo Eleuterio, RobertoMabrouk, RostomAlejo, DavidMabuchi, RyotaAlejos, Ana VázquezMacaire, LudovicAlencastre-Miranda, MoisesMacak, JanAlenin, Andrey S.Macas, MartinAleshin, SergeyMacDermott, ÁineAlessandra, VitanzaMacDessi, SamuelAlet, Pierre-JeanMace, CharlesAletta, FrancescoMacedo, Carina Regina DeAletti, FedericoMacedo, JoaquimAlexander, DockhornMacGregor, Lewis J.Alexandridis, AntonisMachacek, ZdenekAlexandropoulos, GeorgeMachado, Elia A.Alexandropoulou, IoannaMachado, IvanAlexey, YakushenkoMachado, TenreiroAlexiev, KirilMachado, Tiago HenriqueAlexopoulos, KosmasMachaj, JurajAlfano, BrigidaMachikhin, Alexander S.Alfian, GanjarMachno, MagdalenaAlGohi, BasheerMachová, KristínaAlguacil De La Blanca, GerardoMachura, LukaszAl-Hraishawi, HayderMaciak, ErwinAli Shah, HurmatMacías, Elsa M.Ali, AbdulMaciejewski, RyszardAli, AhmadMacioszek, ElżbietaAli, FarmanMaciuk, KamilAli, G. G. Md. NawazMacLennan, DouglasAli, HaiderMacRae, Braid A.Ali, Howbeer MuhamadMacwan, IsaacAli, JamelMadalin, NeaguAli, JehadMadani, MohammadAli, Md LiakatMadeira, JoãoAli, Muhammad MahmoodMadeira, SergioAli, MurtazaMadhurapantula, Rama SashankAli, RashidMadokoro, HirokazuAli, YasirMadruga Saavedra, Francisco JavierAliane, NourdineMaesano, MauroAliaño González, María JoséMaestre Vidal, JorgeAlibakhshikenari, MohammadMaestre, RafaelAlidoost, FatemehMaezawa, KoichiAlifu, HairetiMaga, DušanAlimi, Isiaka AjewaleMagalhães, Fernando HenriqueAlin, BosiocMagarini, MaurizioAlistar, MirelaMagdin, MartinAliyu, AliyuMagliulo, PaoloAljaaf, AhmedMagna, GabrieleAlkan, BugraMagnani, AntonioAlkhalifeh, KhaldounMagne, SylvainAl-Khateeb, HaiderMagner, EdmondAllegra, DarioMagreñán, Ángel AlbertoAllen, JohnMagrinya, Jordi FonollosaAllen-Brady, Kristina L.Magro, CátiaAllendes, RodolfoMahajan, Harshal PrabhakarAlles, Erwin JozefMahajan, SachitAlleysson, DavidMahanti, AniketAl-Majeed, SalahMahapatra, Parth SarathiAlmansa, FranciscoMahata, Manoj KumarAlmeida Ferreira, HugoMahbod, AmirrezaAlmeida Júnior, José HumbertoMahdavi, MohammadAlmeida Marinho, DanielMahdavipour, OmidAlmeida Mendes, Mateus DanielMaher, Louis JamesAlmeida, AitorMaheshwari, MuneeshAlmeida, AnibalMahfuz, SaziaAlmeida, Emerson RodrigoMahler, RonaldAlmeida, FernandoMahmood, TaufiqueAlmeida, JoãoMahmoud, MohammedAlmonacid, BorisMahmud, MuftiAlmond, HelenMahon, Peter J.Almssad, AsaadMahoney, MattAl-Naji, AliMahy, JulienAlomari, AbdullahMaia, Rodrigo FilevAlonso, Alvaro HernandezMaier, Robert R. J.Alonso, CarolinaMaietta, SaverioAlonso, DiegoMaietti, FedericaAlonso, Miguel A.Maimaitiyiming, MaimaitiAlonso, SerafínMaio, LeandroAloqaily, MoayadMair, Lamar O.Alor-Hernández, GinerMairean, CorneliaAlos, JosepMaita, FrancescoAlqahtani, SarraMaiti, Kiran SankarAl-qaness, Mohammed A. A.Maître, JulienAlqudah, YazanMaiwald, MichaelAl-Quraishi, Ayad M. FadhilMajchrowicz, DariaAl-Sadoon, MohammedMajchrzak, TomaszAl-Samman, Ahmed M.Majd, NahidAlshami, Iyad HusniMajeed, AbdulAlsharif, Mohammed H.Majewska, KatarzynaAlshazly, HammamMajewski, Jacek A.Al-Shibaany, ZeyadMajid, RamezaniAlsweiss, SuleimanMajlinger, KornelAl-Tahir, RaidMajstorovic, Vidosav D.Altammar, HussainMajumdar, SuryadiptaAl-temeemy, AliMak, Peng UnAltini, MarcoMakantasis, Konstantinos (Greece)Álvarez Ariza, JonathanMakantasis, Konstantinos (Malta)Álvarez Franco, Fernando J.Makar, ArturAlvarez, DiegoMakarov, Denis V.Álvarez, MarMakarov, MariaAlvarez, RafaelMakek, MihaelÁlvarez-Arenas, Tomás GómezMakhoul, AbdallahAlvarez-Bermejo, Jose AntonioMakino, YoshioAlvarez-Quintana, JaimeMakki, BehroozÁlvarez-Sánches, JoséMakoś, PatrycjaAlveringh, DennisMakowski, LukaszAlves De Oliveira, LucianoMakowski, MichałAlves, José CarlosMakris, ChristosAlves, Luis NeroMaksimovic, FilipAlves, MárioMaksymiuk, KrzysztofAly, KarimMalakooti, MohammadAl-Yaari, AmenMalallah, RaedAl-Yasir, YasirMalara, PietroAlzamora, Fernando MartinezMalde, KetilAmal, HaithamMalecha, KarolAman, Muhammad NaveedMalekian, RezaAmanatiadis, StamatiosMalekmohammadi, HamedAmancio, DiegoMalešević, NebojšaAmano, KinjiroMalik, Amir HaiderAmano, YoshiharuMalina, LukasAmaro, José PedroMalinowska, UrszulaAmato, UmbertoMalka, DrorAmatya, SurajMalkin, RobAmba, PrakharMallorquí, Jordi J.Ambros, JiříMaltese, IlaAmbrosio-lazaro, Roberto CarlosMalvano, FrancescaAmbroziak, AndrzejMalyi, Oleksandr I.Ambrožič, TomažMamrashev, AlexanderAmbrus, RaresManagò, StefanoAmditis, AngelosManaguli, RaviAmelard, RobertManandhar, ShilpaAmer, MohManara, GiulianoAmezquita-Sánchez, Juan PabloManasis, ChristosAmezquita-Semprun, KendrickMancini, EdoardoAmin, FarhanMancini, MarcoAmin, Muhammad JunaidMancini, MaurizioAmin, UllahMancisidor, AitziberAmine, AzizMandal, MrinalAmini, AminMandal, SanghamitraAmiri, SamMandal, SoumyajitAmirov, AbdulkarimMandal, SubhamoyAmirpour, MaedehMandlburger, GottfriedAmit, MoranMandrile, LuisaAmmari, HabibManecy, AugustinAmmer, KurtManenti, SauroAmorim, Vicente J. P.Manera, Maria GraziaAmour, LamineManeuski, DimaAmpatzidis, YiannisManfreda, SalvatoreAmsaad, FathiManfrinetti, PietroAmselem, GabrielManghisi, Vito ModestoAmsters, RobinMangina, EleniAmundarain Irizar, AiertManhart, MichaelAn, Jae-SungMani, Ganesh KumarAn, JiachunManikas, AthanassiosAnagnostopoulos, MariosManiu, Cristina StoicaAnand, VijayakumarManivannan, NadarajahAnanthakrishnan, Soundaram JeevarathinamManjakkal, LibuAnas, Andrea Roxanne J.Mankin, Richard W.Anashkina, ElenaManojlović, Lazo M.Anastasiou, AthanasiosManolova, AgataAnastasopoulos, DimitriosManousakis, NikolaosAnastassiou, CharalambosMansoori, Mohammad AmirAnastassiu, HristosMántaras, Daniel ÁlvarezAnastassopoulos, VassilisMantovani, FilippoAnatolievich Zakharenko, AlekseyMantri, DnyaneshwarAncillao, AndreaManzanares-Lopez, PilarAncin-Murguzur, Francisco JavierManzo, MarioAncione, GiuseppaManzo, MaurizioAncora, DanieleManzoni, CristianAncuti, CosminManzoni, StefanoAndersen, Henning BojeManzoor, AsifAndersen, Jens ChristianMao, ChengshengAndersen, OleMao, HaiyangAnderson, StuartMao, HongjuAndersson, KarlMao, LeiAndo Junior, Oswaldo HideoMao, XiaoanAndò, BrunoMao, XinhuaAndo, KeitaMao, YaoAndolina, Jeffrey R.Mao, Yijie (Lina)Andrade, David CristobalMaran, ViníciusAndrade, Jose ManuelMaranesi, MonicaAndrea, PilottoMarasović, TeaAndreev, SergeyMarateb, Hamid RezaAndreev, ValeriyMarcato, JoséAndrenko, Andrey S.Marcelli, RomoloAndreoli, André LuizMarcelot, OlivierAndreoni, GiuseppeMarch, DavidAndreu, GabrielaMarchand, CédricAndrew, Trisha L.Marchetti, EdaAndrey Andreevich, GolovanMarchevský, StanislavAndriesei, CristianMarchewka, AdamAndrieux, GuillaumeMarciniak, TomaszAndriolo, UmbertoMarciniak-Lukasiak, KatarzynaAndriukaitis, DariusMarcinkevics, ZbignevsAndročec, DarkoMarco, HectorAneece, Itiya P.Marco, MauriAñel, Juan AntonioMarco, SantiagoAnfossi, LauraMarcolin, FedericaAng, Chee SiangMarcon, MarcoAng, Chee WeiMarcos-Martinez, RaymundoAng, Yee SinMarcu, AurelianAngani, Chandra SekharMarcu, IoanaAngelidis, PantelisMarczewski, JacekAngelini, FedericoMarechal, PierreAngelo, AngeloMarek, MaciejAngelopoulos, MariosMares, JanAngelopoulou, AnastasiaMărgărint, Mihai CiprianAnggraini, ViviMargetis, GeorgeAnghelache, GabrielMaria João, SousaAnguelova, Magdalena D.Mariani, PatrizioAnguera, JaumeMariani, StefanoAngulo, CecilioMarianno, CraigAnicho, OgbonnayaMariello, MassimoAnido, LuisMarietta, FodorAnikiev, DenisMarijuán, PedroAnindya, NagMařiková, MonikaAnis, AyalMarin, AndreaAnishchenko, LesyaMarin, EmmanuelAnita Ioana, VisanMarin, FredericAnitas, Eugen MirceaMarin, MarinAnitescu, CosminMarin, PilarAnjos, OféliaMarin, Yisbel E.Annaheim, SimonMarinelli, DanieleAnnaswamy, ThiruMarinello, FrancescoAnnus, PaulMarinesco, StéphaneAnochi, JulianaMarin-Hernandez, AntonioAnsaf, BahaaMarini, FedericoAnsari, KutubuddinMarinkovic, DraganAnselma, LucaMariño, PerfectoAnsó, JuanMarinoni, MauroAnta Álvarez, JoseMarinov, AngelAntal, HibaMarinov, MarinAnthony, CarlMarin-Perez, RafaelAnthony, StephenMarín-Sáez, JuliaAnthopoulos, LeonidasMariotti, LetiziaAntipov, Oleg L.Mariscal, LuisAntoce, Arina OanaMariscotti, AndreaAntolín, DiegoMarkantonakis, KonstantinosAntón, DanielMarkiewicz, JakubAnton, PyrkinMarko Stupin, MarkoAntonakopoulos, TheodoreMarkova, SvetlanaAntonelli, LauraMarkovič, ReneAntoniazzi, FrancescoMarkowski, KonradAntonio, BorgiaMarkowski, PiotrAntonio, Mapuana C. K.Markowski, Piotr MarekAntoniou, JosephinaMarks, Haley L.Antonopoulos, AngelosMarks, RayAntonopoulos, ChristosMarkušić, SnježanaAntonopoulos, Christos P.Marmiroli, BenedettaAntonopoulos, Christos S.Marmugi, LucaAntonovskaya, GalinaMaroto-Molina, FranciscoAntonucci, FrancescaMarotti De Sciarra, FrancescoAntosz, KatarzynaMarozas, VaidotasAntsiperov, Viacheslav EvgenievichMarozzo, FabrizioAntunes, Carla RoloMarpu, Prashanth ReddyAntunes, MárioMarques, CarlosAnuszkiewicz, AlicjaMarques, GoncaloAnvari-Moghaddam, AmjadMarques, GonçaloAnwar, AdnanMarques, Manuel Joaquim BastosAnwar, Syed MuhammadMarques, Manuel JorgeAochi, HideoMarques, Marco LudovicoAoki, YosukeMarques, RuiAparicio-Navarro, Francisco J.Marras, MirkoAppolloni, LucaMarrero, DomingoApruzzese, GiovanniMarshall, Mark T.Aqueveque, PabloMarszalek, ZbigniewArachchige, Hashitha M. M. MunasingheMartalo, MarcoArai, YasuoMärtens, OlevArakawa, MototakaMartian, AlexandruArakawa, TakahiroMartin, BillArakawa, TaroMartin, FerranArakawa, ToshiyaMartin, FranciscoAraki, TeppeiMartín, Jesús SánchezAramesh, MortezaMartin, JimArami, ArashMartín, Joan NavarroArana Arexolaleiba, NestorMartin, LuciAranda, JoaquinMartin, PhilipAranda, Luis AlbertoMartin, SergioArandjelović, OgnjenMartina, CalzavaraAranguren, GerardoMartincic, EmileArani, ArvinMartín-Clemente, RubénArashloo, Shervin R.Martindale, Christine F.Araszkiewicz, AndrzejMartin-del-Campo, SergioAraujo, AlvaroMartinek, RadekAraújo, DuarteMartinelli, AgostinoAraújo, EvandoMartinelli, AlessioAraujo, JeffersonMartinelli, GiovanniArazuri, SilviaMartinet, JeanArbabi, VahidMartinetti, AlbertoArchMiller, Althea A.Martinez Espinosa, Juan CarlosArcia-Moret, AndrésMartínez García, MiguelArdigò, Luca PaoloMartínez Jiménez, JavierArdila-Rey, Jorge AlfredoMartínez López, José IsraelArdjmand, EhsanMartínez Peláez, RafaelArellanes, DamianMartinez, BorjaArellano, Giovanna MartinezMartinez, JoaquinArena, AntonellaMartinez, KirkArena, FabioMartinez, ManuelArena, MaurizioMartínez-Barberá, HumbertoArend, Mark F.Martinez-Carranza, JoséArgüelles, AmadeoMartínez-Carricondo, Patricio J.Arguelles-Cruz, Amadeo J.Martínez-De-Dios, José RamiroArgüello, FranciscoMartinez-de-Rioja, EduardoAri, Ado Adamou AbbaMartínez-García, GonzaloArias Garcia, JanierMartínez-Hinarejos, Carlos-D.Arias-Estrada, Miguel-OctavioMartinez-Izquierdo, EstibalizAricò, PietroMartínez-Lillo, JoséArifoglu, DamlaMartinez-Martin, EsterArino, ChristinaMartinez-Mora, Juan AntonioAriyarit, AtthaphonMartínez-Nova, AlfonsoAriyawansa, GaminiMartínez-Otzeta, José MaríaAriyur, Kartik B.Martínez-Pellitero, SusanaAriza Quintana, AlfonsoMartínez-Sala, Alejandro SantosArjoune, YounessMartin-Fernandez, CristianArjunan, SridharMartín-Gorostiza, ErnestoArkwright, JohnMartini, AlbertoArmada, ManuelMartini, AndreaArmada, Manuel A.Martini, ElisabettaArmando, NgomboMartin-Lopez, SoniaArmen, Ter-MartirosyanMartín-Mateos, PedroArmijo, Juan FranciscoMartino, José Mario DeArmitage, RachelMartino, LucaArnal Barbedo, Jayme GarciaMartino, Tartaglia GianlucaArnaubec, AurélienMartinot, Jean-BenoitArnaud, GouelleMartinotti, SimonaArnold, Francisco JoséMartins Afonso, MarcoArnon, ShlomiMartins, AlfredoArnusch, ChristopherMartins, ElianeArrabito, Giuseppe DomenicoMartins, Fernando RamosArrebola, ManuelMartins, José A.Arrigoni, MichelMartins, MarcosArroyo Cardoso, FilipeMartins, NataliaArroyo, PatriciaMartinsen, Ørjan G.Arrue, Begoña C.Martins-Filho, Joaquim F.Arsalan, MuhammadMartorell, Jaume VerdArtale, GiovanniMartowicz, AdamArtal-Sevil, Jesús SergioMartucci, MatteoArtan, UnalMartynenko, AlexArtese, SerenaMarucci, AlessandroArteta, AlbertoMarucci, AlvaroArtiemjew, PiotrMaruccio, GiuseppeArtola, LaurentMaruda, Radosław W.Artur, GrządzielMaruschak, PavloArtusi, Carlo AlbertoMarzano, AdelaideArvanitis, KonstantinosMarzano, GilbertoArvanitoyeorgos, AndreasMarzband, MousaArvinti, BeatriceMarzbanrad, FaezehArya, Sunil K.Marzi, ClaudiaArza Valdés, AdrianaMarzullo, Vincenzo ManuelArza, MarcosMas, DavidAsadi, BehzadMasaracchia, AntoninoAsadnia, MohsenMasarati, PierangeloAsakura, TakumiMasayuki, MukunokiAsanuma, IchioMasayuki, SatoAsch, TheodoreMasayuki, SohgawaAschonitis, Vassilis GeorgeMascio, Tania DiAsfin, Ruslan E.Mascort-Albea, Emilio J.Asgary, AliMašek, PavelAshfaq Khan, MuhammadMasek, VlastimilAshiba, HirokiMasi, SofiaAshiquzzaman, AkmMasia, LorenzoAshley, JonMasiero, AndreaAshok, AshwinMasini, Barbara M.Ashraf, ImranMasini, Jorge CesarAshraf, NabilMasip, DavidAsif, RameezMasip, YuneskyAskari, SinaMaskeliūnas, RytisAslam, MuhammadMaslen, Eric H.Asorey-Cacheda, RafaelMaslov, MikhailAssaf, TareqMasłyk, MaciejAssante, DarioMasoero, MarcoAssefzadeh, MahdiMaspero, FedericoAssis, Karcius Day RosárioMasquelier, TimothéeAssunção, PedroMassari, AnthonyAstasio-Picado, ÁlvaroMassaro, AlessandroAstolfi, DavideMassaro, SebastianoÅstrand, ElaineMassaroni, CarloAtanasoae, PavelMastinu, EnzoAtanasov, Petar AsenovMas-Torrent, MartaAtanassov, KrassimirMastrandrea, RossanaAtapour-Abarghouei, AmirMastronardi, Vincenzo MarianoAta-Ul-Karim, Syed TahirMastropietro, AlfonsoAthavale, YashodhanMasurkar, NirulAtienzar, PedroMata Miquel, ChristianAtoche, Alejandro CastilloMatasaru, Petre DanielAtri, RoozbehMatatagui, DanielAttia, AhmedMataveli, Guilherme Augusto VerolaAttila, FazekasMateev, ValentinAttilio, ParisiMateo, Carlos M.Aubin, PatrickMateos, CristianAugousti, AndyMaterer, Nicholas F.August, IsaacMaterka, AndrzejAugustynek, MartinMatetić, MajaAulicino, GiuseppeMateu, JorgeAuslender, MarkMateus, PedroÁvalos, Juan GerardoMatheve, ThomasAvanaki, KamranMathew, BobbyAvanaki, Mohammad R. N.Mathew, JoiceAvanthey, LoïcaMatić, TomislavAverta, GiuseppeMatijošius, JonasAvezov, EdwardMatko, VojkoAvignon-Meseldzija, EmilieMatos Mascarenhas, DalbertÁvila, José Pablo ChavesMatos, Joao NunoAvila-Paredes, Hugo J.Matos, TiagoAviles-Espinosa, RodrigoMatran-Fernandez, AnaAvina-Cervantes, Juan GabrielMatras, AndrzejAvizzano, Carlo AlbertoMatricciani, EmilioAvola, DaniloMatsubara, Edson TakashiAvram, CameliaMatsugi, AkiyoshiAvram, MarioaraMatsuguchi, MasanobuAvtar, RamMatsumura, KentaAw, KeanMatsuo, HiroshiAwad, Ali IsmailMatsuoka, MasashiAwad, MohamadMatsushima, JunAwate, SanketMatsuura, KazunoriAwin, FaroqMatsuura, YujiAwolusi, Ibukun GabrielMattarelli, MaurizioAwrejcewicz, JanMatteo, MatteucciAxenie, CristianMattfeld, RyanAyankojo, Akinrinade GeorgeMatthews, AlanAydin, ÜmitMatulin, MarkoAyesta, IgorMaturi, EileenAyoub, NaeemMatusik, PawełAysu, AydinMatveev, SergeyAzadegan, AidaMatys, JacekAzam, Aa HaerumanMaulini, RichardAzamathulla, Hazi MohammadMauri, Pedro VicenteAzar, MassoodMauricio, JoanAzaria, AmosMaurício, JoséAzetsu, TadahiroMaurits, Natasha M.Azevedo, FábioMauriz, ElbaAzimi, Seyyed MohammadrezaMauro, StefanoAziz, Abdul RashidMavridis, IoannisAziz, AhmedullahMavromatidis, LazarosAziz, FayeemMavromatis, IoannisAzorín-López, JorgeMavromatis, SébastienAzurdia, CesarMax, NelsonAzzedin, FaragMaximiano, MarisaBaba, AsifMay, James MichaelBabić, AnjaMaybeck, VanessaBabichev, SergiiMayer, JoceliBabušiak, BrankoMayer, PhilippBača, MiroslavMayer, SimonBacanin, NebojsaMaynadier, AnneBaccarelli, EnzoMaynard, PeterBacciocchi, MicheleMayor, JesusBacco, DavideMazaheri, ArashBacco, ManlioMažeika, DaliusBaccour, NouhaMazeika, LiudasBacellar, DanielMazumdar, Ellen Yi ChenBacem, MbarekMazumder, AbhishekBachelder, EdwardMazurek, MiroslawBachhofner, StefanMazurek, PawełBachman, HunterMazurek, PrzemysławBačić, MarioMazza, FabioBączkowicz, DawidMazzarella, GiuseppeBadar, MudabbirMazzaro, GregoryBadau, DanaMazzei, DanieleBaddour, NatalieMazzetto, FabrizioBadea Doni, MihaelaMazzola, Silvia MichelaBadea, Ana CorneliaMazzulla, GabriellaBadea, MihaelaMcAlister, EmmaBader, Brett W.McAndrew, IanBader, SebastianMcCamley, JohnBadescu, GabrielMcCamley, John D.Badia-Melis, RicardoMcClarren, RyanBae, HyungdaeMcCool, ChrisBae, Ihn-hanMcDanel, BradleyBae, JihyunMcDermott, BarryBae, Jong-HoMcDonald, TimothyBae, Sang WonMcewan, AlistairBae, WonMcgibbon, ChrisBae, YoungchulMcGinnis, EllenBae, YoungsookMcGrath, DanielBaek, Kwang RyulMcGrath, RyanBaek, SeungryulMcheick, HamidBaek, Soo-WhangMcIntyre, Dustin L.Baena, Francisco ManuelMcKee, KristofferBaena, SusanaMckelvey, KimBaerwolff, GuenterMcLagan, DavidBaets, Liesbet DeMcLamore, Eric S.Baeyens, JanMcLaughlan, JamesBaffa, OswaldoMcLeod, EuanBagal-Kestwal, Dipali R.McManus, Barry J.Bagassi, SaraMcNamara, ShamusBagdziunas, GintautasMcPheron, Benjamin D.Baghdadi, AliMd.Nuruddin Hasan, DihanBaghdadi, AmirMeacci, ValentinoBaghdadi, NicolasMeaney, PaulBaghdasaryan, TigranMeder, FabianBaghelani, MasoudMedina Quero, JavierBagrov, Alexander V.Medina, DanielBagui, SikhaMedina, RubenBahmani, HamedMedola, Fausto OrsiBahmani, RaminMedrano, NicolásBahsi, HayretdinMedved, VladimirBai, BaomingMedvedev, IvanBai, BofengMedvedev, ViktorBai, HeMedvidović-Kosanović, MartinaBai, LongMeena, Nand K.Bai, OuMegalingam, Rajesh KannanBai, ShaopingMeggio, FrancoBai, XianxuMegret, PatriceBaig, ImranMehmood, FaizanBaig, Muhammad ZeeshanMehmood, RashidBaiguera, StefanoMehrabi, ArminBaik, SungMehran, AliBailly, GérardMehrmohammadi, MohammadBain, Colin D.Mehrotra, PrakharBaiocchi, ValerioMei, HaiboBais, AbdulMei, HanfeiBajer, JosefMei, LeiBaji, ZsófiaMei, XuesongBajic, DraganaMei, YuBajpai, RamMeikle, WilliamBakdi, AzzeddineMeireles, TiagoBaker, BenMeissner, MiroslawBaker, TharMejia Rincon, LeonardoBakhshande, FatemeMejia-Beltran, EfrainBakr, MustafaMejía-Salazar, Jorge RicardoBakuła, MieczysławMekhrengin, MikhailBala, CameliaMekikis, Prodromos-VasileiosBalabhadra, SangeethaMekinda, LeonceBalado Frias, JesusMelandso, FrankBalado Frías, JesúsMelchiori, MicheleBalaguru, RajaMelchor, JuanBalakrishnan, DeepanMelendez-Pastor, IgnacioBalal, NezahMelgarejo Meseguer, Francisco ManuelBalan, OanaMelgoza-Vázquez, EnriqueBalan, TitusMeli, EnricoBalawejder, MonikaMelillo, PaoloBalazia, MichalMelkonian, Jean-MichelBalbekin, NikolayMello, LuizBalbi, BrunoMelnikov, PavelBalchanos, MichaelMelnykowycz, MarkBalcik, Filiz BektasMelo, PedroBalda, PavelMemisoglu, GorkemBăldean, Doru-LaureanMemmolo, VittorioBaldi, SimoneMemon, Faisal AhmedBaldi, Tommaso LisiniMemon, HafeezullahBaldini, GianmarcoMemon, ImranBalducci, FabrizioMénard, Jean-MichelBalen, TiagoMenasalvas, ErnestinaBalestrieri, EulaliaMencik, Jean-MathieuBalevicius, ZigmasMendecki, LukaszBalint, CornelMendes Junior, José Jair AlvesBalitskii, AlexanderMendez Zorrilla, AmaiaBallard, ZacharyMendez, Martin OswaldoBallato, JohnMéndez, RoiBalleri, AlessioMendez-Astudillo, ManuelBallesta Galdeano, MonicaMendicute, MikelBalletti, CaterinaMendis, GihanBallmann, ChristopherMendizabal, JaizkiBalogh, RichardMendoza, Daniel L.Balsa-Barreiro, JoseMendoza-Acevedo, SalvadorBalsi, MarcoMendyk, AleksanderBalss, UlrichMenegatti, EmanueleBalzer, FrankMeneghetti, AlessioBamiedakis, NikosMeneghetti, GiovanniBanan Sadeghian, RaminMenegoni, NiccolòBanaszak, ZbigniewMenesidou, Sofia-AnnaBanaszek, AnnaMenezes Morato, MarceloBandera, JuanMeng, ChaoBando, YoshiakiMeng, FanliBandodkar, Amay J.Meng, FanyuBandyopadhyay, SupriyoMeng, GangBanerjee, RumanMeng, GuowenBanerjee, SouravMeng, LinBanfield, DonMeng, LingjuBang, HyochoongMeng, QianBani Younes, AhmadMeng, WeiBani Younes, MaramMeng, WeixiaoBaniata, HamzaMeng, WeizhiBanitsas, Konstantinos A.Meng, XiangyuBankov, DmitryMeng, XuBannov, AlexanderMeng, YuBañón Arias, Sebastián Del PilarMeng, YuanBañón, LuisMeng, ZhaozongBanterle, FrancescoMeng, Zi-MingBanville, SimonMengarelli, AlessandroBao, Jing-fuMengistie, Desalegn AlemuBao, XiaoyiMenichetti, MarcoBao, YiMenon, Varun G.Bao, YuhaiMenzel, Siegfried BernhardBar, IlanaMeoni, GabrieleBarabino, BenedettoMera, José ManuelBarakabitze, Alcardo AlexMerazzo, KarlaBarakate, AbdellahMercader, PedroBaranowski, PawełMercado, Jesús M.Baranyai, NóraMercante, LuizaBarata, JoãoMercer, John A.Barati, MasoudMerchán, PilarBaray, Jean LucMercorelli, PaoloBarb, Adrian S.Merdrignac, PierreBarba, Marta GilMerenda, MassimoBarbancho, JulioMerico, DavideBarbarin, YohanMeriggi, PaoloBarbato, MarcoMerino, LuisBarbé, KurtMerino-Martinez, RobertoBarber, RamónMerlen, AlainBarber, SarahMeron, MosheBarberis, AntonioMerrick, TrinaBarbin, DouglasMertzimekis, TheoBarbinta-Patrascu, Marcela ElisabetaMerz, CliffordBarbon Junior, SylvioMesas Carrascosa, Francisco JavierBarbosa, Ramiro S.Meseguer, RocBarbosa, Roberto SpinolaMeshkinzar, AtaBarca, EmanueleMešić, AleksandarBarcik, PeterMesodiakaki, AgapiBarclay, DavidMessier, Kyle PBarczak, KamilMessineo, LudovicoBarek, JiriMester, ChristianBarentine, John C.Metcalfe, BenjaminBarez, FredMetelka, RadovanBarile, ClaudiaMeulenberg, ElineBarillaro, GiuseppeMeuser, TobiasBarille, RegisMeyer-Aurich, AndreasBarkalov, OleksandrMeyers, GregoryBarkjohn, Karoline K. JohnsonMeynants, GuyBarletta, Vita SantaMeza-Ruiz, Iván VladimirBarmada, SamiMezei, IvanBarmparis, Georgios D.Meziani, YahyaBarmpoutis, AngelosMezzina, GiovanniBarmpoutis, PanagiotisMi Lee, BoBarniol, NúriaMiądlicki, KarolBarnoaiea, IonuţMiani, Rodrigo SanchesBaron, CoreyMiao, GuoxingBarone, Pier MatteoMiao, QingqingBarquero-Pérez, ÓscarMiao, YinghaoBarra, AdrianoMiao, YuBarraca, João PauloMiao, ZelangBarrado, CristinaMiao, ZhiqiangBarral, ValentínMiao, ZhonghuaBarrasso, RobertaMiąsik, PrzemysławBarrau, AxelMiccio, Luis A.Barreca, SalvatoreMiccoli, GiuseppeBarreira, EvaMichael Ho, Siu ChunBarrera, DavidMichael, OrtnerBarrera, EduardoMichaelides, Michalis P.Barresi, GiacintoMichailidis, Emmanouel T.Barreto, LuísMichal, Carl A.Barreto, MarcosMichalak, PiotrBarrett-Gonzalez, RonaldMichalas, AntonisBarrios, MaiteMichau, GabrielBarrios, MiguelMichel, AndreaBarriot, Jean-PierreMichel, Carlos R.Barro, Pape AbdoulayeMichel, PiotrBarroso, MargaridaMichie, CraigBarsanti, MicheleMichihata, MasakiBarski, MarekMichler, OliverBarsocchi, PaoloMickiewicz, WitoldBartecki, KrzysztofMiclaus, SimonaBarth, Nicolas C.Middleton, Stuart E.Bartkowska, AnetaMidoglu, CiseBartlett, Michael D.Mieczkowski, GrzegorzBartlomiej, OszczakMielgo-Ayuso, JuanBartoletti, CiprianoMieloszyk, MagdalenaBartolic, JurajMietzner, JanBartoněk, DaliborMigallón, HéctorBartonova, AlenaMigniot, CyrilleBartusek, KarelMiguel, Gonzalo DeBartyś, MichałMihai, AndruscaBarua, ShaibalMihai, DuguleanaBarukčić, MarinkoMihai, Ilie ValentinBarz, CristianMihaicuta, StefanBarzilov, AlexanderMihail, Radu PaulBas, JoanMihailescu, Marius IulianBasak, ShibajiMihailescu, Radu-CasianBasan, ElenaMihajlović, VojkanBasarab, MikhailMihelj, MatjažBasbas, SocratesMika, AnnaBasford, Philip JamesMikelsons, KarlisBashkatov, Alexey NikolaevichMikhaylov, KonstantinBasil, ChristarasMikhelson, KonstantinBasile, FrancescoMikkelsen, Bent EgbergBasile, ValerioMikkelsen, JanBasova, TamaraMikkelsen, KaareBassani, CristianaMikkonen, JussiBastianini, FilippoMikno, ZygmuntBastos, Guilherme S.Mikolajczyk, TadeuszBastos, Teodiano FreireMikulski, JerzyBaszanowska, EmiliaMikuš, TomislavBatalla, Jordi MongayMikusova, MiroslavaBatcho, Charles SebiyoMilanesio, MarcoBatchuluun, GanbayarMilanic, MatijaBatistuti, Marina RibeiroMilanova, MariofannaBatra, Ashok K.Milardo, SebastianoBattaglia, FilippoMilazzo, MarioBattineni, GopiMilde, MoritzBattistini, SimoneMilena Faria, PintoBaù, MarcoMiler, MarioBauer, FrancoisMileti, IlariaBauer, MartinMilicevic, MarioBauer, RomanMilici, StefanoBauer-Marschallinger, BerhardMilikić, JadrankaBaumer, StefanMiliucci, MarcoBaumgartner, WernerMiljković, NadicaBausch, NilsMillan Almaraz, Jesus RobertoBausys, RomualdasMillar, PhillipBautu, ElenaMiller, BartoszBayat, NozhanMiller, Benjamin L.Baykal, YahyaMiller, BorisBayoumi, MagdyMiller, GregoryBayram, AbdullahMiller, JimmieBazazian, DenaMills, Amanda C.Bazzi, AhmadMilne, DavidBazzi, AlessandroMilosavljević, AleksandarBeach, RobertMiltiadou, MiltoBeale, DavidMilz, StefanBeall, Cynthia M.Min, KyunghaBear, LauraMin, MartBeard, CoryMin, RuiBeata, ZimaMin, Se DongBeato-López, Juan JesusMin, WeiqingBeaudette, ShawnMin, YunhongBęben, AndrzejMinaee, ShervinBeben, DamianMinami, TsuyoshiBeberok, ArturMinamiki, TsukuruBec, KrzysztofMinardo, AldoBecek, KazimierzMinářová, JanaBechhoefer, EricMinas, GraçaBeck, Brian M.Minea, MariusBecker, ClemensMinelli, AlbertoBecker, Leandro BussMingesz, RobertBecker, MarceloMingotti, AlessandroBeckers, ThomasMinguella-Canela, JoaquimBeckingham, BryanMinh, Le VanBecucci, MaurizioMiniaci, MarcoBȩdkowski, Janusz MarianMinin, Igor V.Beeby, SteveMinnaert, BenBeecks, ChristianMinussi, Carlos RobertoBeer, MartinMinzu, ViorelBeeri, OferMioshi, EneidaBeg, MarijanMiranda, HugoBeg, NazmulMiranda, João M.Begdache, LinaMiranda, JorgeBégel, ValentinMirarchi, ClaudioBeghi, RobertoMirás-Avalos, José ManuelBegušić, DinkoMiridonov, SergueiBehal, AmanMirjalili, AliBehbahani, Amir HosseinMirkes, Evgeny M.Behrens, TristanMirri, SilviaBeier, GrischaMirsky, Vladimir M.Beilina, LarisaMirsky, YisroelBeirão, PedroMirtaheri, PeymanBeise, Hans-PeterMirvakili, Seyed M.Bejger, ArturMirzavand, RashidBejmert, DanielMishra, AnandBekasiewicz, AdrianMishra, Geetesh K.Belagiannis, VasileiosMishra, HarshadBelaïd, AbdelMishra, Kumar VijayBelciu, AndaMishra, Pawan KumarBelforte, GuidoMishra, Sandeep KumarBelias, DimitriosMishra, Satyendra KumarBeliatis, Michail J.Mishra, Yogendra KumarBell, JohnMishra, Yogeshwar NathBell, TylerMisiurewicz, JacekBellagente, PaoloMišković, NikolaBellanca, GaetanoMiskowicz, MarekBellasio, RobertoMisra, GauravBelli, PierluigiMissell, FrankBellido Outeiriño, Francisco JoséMissell, Frank P.Bellman, ChrisMisson, MichelBellone, MauroMistewicz, KrystianBellucci, PatriziaMistretta, FaustoBelmonte Fernández, OscarMistry, KamleshBelmonte, Jesús LópezMisul, DanielaBeltrame, GiovanniMitani, AtsushiBeltran-Carbajal, FranciscoMitchell, CatherineBen Gal, AlonMitchell, John S.Ben Haj Frej, MohamedMitishita, EdsonBen Yoav, HadarMitka, BartoszBen, YueyangMitobe, KazuhisaBeňa, ĽubomírMitra, KunalBenalia, SourayaMitschke, ChristianBenavente-Peces, CésarMitsou, VasilikiBenbouzid, MohamedMitsui, ToshiyukiBencardino, DanielaMitsukura, YasueBenchirouf, AbderrahmaneMitterer, TobiasBenco, MiroslavMitton, NathalieBendechache, MalikaMitura, AndrzejBender, PaulMiura, KazumasaBenderius, OlaMiyagusuku, RenatoBenedetti, SimonaMiyamoto, IkuyaBenedicenti, LuigiMiyazaki, HiroyukiBenedikovic, DanielMiyazaki, TomoBenéitez, Nuria TeigellMizsei, JánosBenetti, MassimilianoMizuguchi, NobuakiBengson, Jesse J.Mizuno, IkuoBengtsson, EwertMjahed, MostafaBeni, ValerioMkaouer, Mohamed WiemBenissa, SaidMladenov, ValeriBenítez Andrades, Jose AlbertoMladenović, Goran M.Benitez, Victor H.Młyńczak, MarcelBenitez-Garcia, GibranMlynek, JaroslavBenitez-perez, HéctorMlýnek, PetrBenko, BožidarMłyński, DariuszBennet, FrancisMo, ChangyeunBennett, MichaelMo, JinhanBennett, Steven P.Mo, LingfeiBen-Othman, JalelMo, YuBenoussaad, MouradMoacir Godoy, MoacirBenseman, TimothyMoafipoor, ShahramBenyahia, IlhamMoar, WilliamBeranek, LadislavMobashsher, Ahmed ToahaBerbecaru, DianaMobed-Miremadi, MaryamBereś-Pawlik, ElżbietaMobilia, MirkaBereza-Malcolm, LaraMobilio, MarcoBergamaschi, AnnaMocanu, Dan-AdrianBerger, ChristianMocanu, IrinaBerger, CyrilleMocanu, Irina GeorgianaBergman, AndersMocanu, MarianaBergmann, JeroenMoccia, MarcelloBergmann, OlafMoccia, SaraBeriáin, AndoniModenese, AlbertoBerinskii, IgorModenini, DarioBeritelli, FrancescoModica, Giuseppe DiBerkó, AndrásMoein, HadiBernabe, Jorge BernalMoga, LigiaBernacki, BruceMoghimi, MohammadBernal Escobedo, LuisMogi, KatsuoBernal, OlivierMoh, SangmanBernard, BrianMohamed-Seghir, MostefaBernardini, GabrieleMohammad, MahdaviBernardo, Luis Filipe AlmeidaMohammadi, BabakBernaś, MarcinMohammadi, FazelBernatavičienė, JolitaMohammadi, Mohammad EbrahimBernhard, StefanMohammed, AhmedBerntzen, LasseMohammed, NabeelBerretti, StefanoMohanta, AntaryamiBerrocal, JavierMohd-Yasin, FaisalBerry, Paul E.Mohino-Herranz, InmaBertaccini, AssuntaMohns, EnricoBertemes-Filho, PedroMohorcic, MihaelBertini, FlavioMohsen, Marani BarzaniBerto, MarcelloMois, GeorgeBertocci, FrancescoMoiseev, SergeyBertolin, ChiaraMoisescu, Mihnea AlexandruBertoncello, PaoloMoitzi, GerhardBertoni, AndreaMok, Sung-HoonBeruete, MiguelMokhlespour Esfahani, Mohammad ImanBeruvides, GerardoMolardi, CarloBerveglieri, AdilsonMoldoveanu, FloricaBesada, JuanMolento, CarlaBesançon, LonniMolimard, JérômeBeskopylny, AlexeyMolin, Jose PauloBessa, Iury ValenteMolina López, JoseBessmertnykh-Lemeune, AllaMolina, FranciscoBessonov, Vladimir O.Molinos, Eduardo JoseBestak, RobertMoll, JochenBétaille, DavidMöller, SörenBettati, StefanoMollica Nardo, VivianaBetthauser, Lisa M.Mollica, CristinaBettocchi, CarloMolnar, ArthurBeverini, NicoloMolnár, GyörgyBevitt, JosephMolteno, TimBeyabanaki, Amir RezaMolter, DanielBeyerle, GeorgMoltó, GermánBezantakos, SpirosMon, Yi-JenBezerra, Eduardo A.Monaco, Maria Grazia LourdesBezzateev, SergeyMonaco, VitoBhalla, NikhilMondal, SudipBhamra, TracyMonica, RiccardoBhandari, Manohar PrasadMoñino, AntonioBharatharaj, JaishankarMonji, AkiraBhargava, KritiMonk, StephenBharti, PratoolMonnawar, MustafaBhat, GanapatiMonno, YusukeBhatt, Vijay DeepMonobe, HirosatoBhattacharyya, MayukhMonowar, Muhammad MostafaBhattarai, Jay K.Monreale, AnnaBhethenabotla, VenkatMonroe, DerekBhounsule, Pranav A.Monsalve, AlbertoBhowmik, BasurajMonsuez, BrunoBhowmik, Lal MohanMonsurrò, PietroBhowmik, NeelanjanMontana, JessicaBhuckory, ShashiMontangero, ManuelaBhuiyan, Md YeasinMonte Lima, João Paulo Silva DoBhutta, Muhammad RaheelMonteiro, DiogoBi, ChuanxingMonteiro, FilipaBi, HongshengMonteiro, JoãoBi, JinshunMonteleone, SalvatoreBi, KeMontella, RaffaeleBi, XiangMontenegro, Pedro AiresBi, YunboMontero De Espinosa, FranciscoBia, PietroMonterroso-Checa, AntonioBiagetti, GiorgioMontes García, VerónicaBialas, KatarzynaMontes, HectorBian, JinhuMontes, MarcosBian, LihengMontezuma-Carvalho, PauloBianchi, ValentinaMonti, DanielaBianchini Ciampoli, LucaMontisci, AugustoBianco, SimoneMontoliu, RaúlBiancolillo, AlessandraMontori, FedericoBianconi, FrancescoMontoya, Oscar DaniloBiasiucci, AndreaMontrucchio, BartolomeoBiasotti, SilviaMonzo, CarlosBibbo, DanieleMonzon Verona, Jose MiguelBica, IoanMooij, ErwinBicen, OzanMook Kang, ChangBie, HongxiaMookiah, Muthu Rama KrishnanBie, RongfangMoon, HyejinBielecka, MarzenaMoon, HyeonjoonBieniek, AndrzejMoon, Jong-MinBieniek, TomaszMoon, JongsubBiermann, FelixMoon, Kee S.Biermann, PeterMoon, KiwonBifulco, PaoloMoon, MinkyuBigi, AlessandroMoon, Seong-EunBiglia, AlessandroMoon, YaejinBikakis, AntonisMooney, JakeBilal, MuhammadMoore, PhilipBilas, VedranMoore, WayneBilican, DogaMoorman, ValerieBilicki, VilmosMoosazadeh, MahdiBilík, PetrMora, AndreBilleci, LuciaMora, Elena VillalbaBilo, GrzegorzMora, HiginioBin, ShengMora, NiccolòBinetti, LeonardoMorabito, Francesco CarloBinzhong, ZhouMoraes, ChrisBiondi, FilippoMoraes, Fernando CruzBiondi, GiulioMoraes, Francisca Pereira DeBirch, BrianMoraes, Ricardo Alexandre Reinaldo DeBirch, ByrennMoragrega, AnaBirch, David M.Morais, RaulBird, JordanMoral, CristianBirdal, TolgaMorales Maqueda, Miguel ÁngelBirk, AndreasMorales Narváez, EdenBiro, IstvanMorales, NéstorBisaglia, CarloMorales-Navarrete, HernánBischof, Hans-PeterMora-Macías, JuanBisharat, Dia’aaldinMorar, AncaBisi, Maria CristinaMora-Rodriguez, JesusBisogni, Maria GiuseppinaMoraru, LuminiţaBisson, MarinaMorasso, CarloBiswas, KamanashisMoratuwage, DilukaBiswas, ShantonuMoraveji, NeemaBiswas, SumanaMorávková, ZuzanaBitar, Ahmad W.Morbidoni, ChristianBithas, Petros S.Morega, MihaelaBittolo Bon, SilviaMoreira Gonçalves, LuísBiziuk, Marek K.Moreira, AdrianoBizon, NicuMoreira, Adriano J. C.Bizzego, AndreaMoreira, Adriano Jorge CardosoBjörkelund, AndersMoreira, FernandoBjorklund, RobertMoreira, JoaoBjörkqvist, Jan-VictorMoreira, Mário Wedney De LimaBjörling, Elin A.Moreira, Rui SilvaBjörninen, ToniMorel, PascalBlachowski, BartlomiejMorelli, DavideBlahut, JanMorello, RosarioBlake, JacquelineMoreno García, María N.Blanchard, FrançoisMoreno, AsierBlanco Rojas, Maria DoloresMoreno, Carlos LuqueBlanco-Novoa, OscarMoreno, FranciscoBlandin, RémiMoreno, JaimeBlandón-Naranjo, LucasMoreno, VidalBlanes, CarlosMoreno-Armendáriz, Marco A.Błaszczak-Bąk, WioletaMoreno-García, CarlosBłaszczyszyn, MonikaMoreno-Guerrero, Antonio-JoséBlazej, JosefMoreno-Hernández, DavidBlazek, JosebMoreno-Moreno, Carlos D.Blazek, ThomasMoreno-Valenzuela, JavierBlazevic, DavidMorere, YannBlažič, SašoMoret-Tatay, CarmenBlazina, MariaMoretti, FedericoBloch, VictorMorettini, MicaelaBlock, RobertMoretto, SandraBlock, StephanMorgado, António MiguelBlondel, PhilippeMorgan, StephenBloom, GedareMorganti, FabriceBlumenschein, Laura H.Morgenthal, GuidoBlumenstein, JiriMorgera, Alessandro FraleoniBlumrosen, GaddiMoriello, Rosario Schiano LoBlüthgen, ChristianMorillas Gómez, SamuelBlyakhman, Feliks AbramovichMorillo, Daniel SánchezBo, FangMorimoto, TaniaBoada, Beatriz L.Morimoto, YoshiharuBoaga, JacopoMoríñigo-Sotelo, DanielBoardman, Richard PMorino, HiroakiBoavida, FernandoMorishita, ShinichiroBobacka, JohanMoritz, Guilherme LuizBobashev, GeorgiyMoriuchi-Kawakami, TakayoBobda, ChristopheMoriyama, ToshifumiBobin, JéromeMorley, Nicola A.Bobinger, MarcoMor-Mur, MontserratBobkowska, KatarzynaMornar, AnaBoboc, Răzvan GabrielMornieux, GuillaumeBobulska, LenkaMoron, CarlosBobulski, JanuszMoroń, TomaszBoccia, LorenzoMoroni, DavideBochkati, MohamedMoroni, MonicaBocîrnea, Amelia ElenaMorosi, JacopoBock, Joel R.Morosi, SimoneBocková, MarkétaMorozov, OlegBocu, RazvanMorozov, Oleg G.Bodaghi, MahdiMorozova, AnnaBoddaert, XavierMorozova, SofiaBoenisch, GerhardMorozs, NilsBoffi, PierpaoloMorrell, BenjaminBogaerts, JanMorris, DanielBogataj, MarijaMorris, RosieBogawski, PawełMorris, TimBogdan, IleanaMorrone, AldoBogdan, MihaiMorsy, SalemBogdan, PaulMortara, MichelaBogdan, RazvanMorthier, GeertBogliolo, AlessandroMortimer, MichaelBognár, GabriellaMosa, IslamBogris, AdonisMosavi, AmirBöhm, LutzMosbah, MohamedBöhnert, TimMoscalu, MihaelaBohush, RykhardMoscato, VincenzoBojkovski, JovanMoschitta, AntonioBok, SanghoMoscholios, Ioannis D.Bokde, NeerajMoselund, Peter M.Boland, Francis MorganMoser, HaraldBolanowski, MarekMoser, KennethBolboacă, Sorana D.Moshou, AlexandraBolbot, VictorMosiej, JozefBollinger, LanceMoskalev, PavelBolognesi, Cecilia MariaMosleh, AraliyaBolomey, Jean-CharlesMoslem, SarbastBölöni, LadislauMoss, DonaldBolotin, Yu V.Mossa, GiorgioBolshov, MikhailMosser, VincentBoltes, MaikMossi, KarlaBoluda, Jose A.Mostafaei, Habib (Germany)Bombek, GorazdMostafaei, Habib (Italy)Bona, StefanoMostarac, PetarBonaci, TamaraMota, Guilherme Lucio AbelhaBonafoni, StefaniaMota, Rafael Perazzo BarbosaBonasia, RosannaMota, Virgínia FernandesBonastre Pina, Alberto MiguelMotahhir, SaadBoncel, SlawomirMotogaito, AtsushiBonci, AndreaMottola, FabioBonci, TeclaMoualeu-Ngangue, Dany P.Boncinelli, LeonardoMoudry, VítezslavBond, JasonMouli, RamasamyBondavalli, PaoloMoura, JoseBondorf, SteffenMourad-Chehade, FarahBone, GaryMouratidis, DespoinaBonet, ElisendaMourtzios, ChristosBonfè, MarcelloMourtzis, DimitrisBongo, Lars AiloMourya, RahulBonifácio, Luís Filipe PrazeresMousas, ChristosBonifazi, GiuseppeMousavi Kahaki, Seyed MostafaBońkowski, PiotrMousavi Lajimi, S. AmirBonomo, Anthony L.Mousavi, AminBonomo, MatteoMoussas, Vassilios C.Bonomolo, MarinaMoustafa, AhmedBonyár, AttilaMoustafa, MohamedBoolani, AliMoustakas, MichaelBoon, ChengMoutarde, FabienBoori, Mukesh SinghMoutinho Silva, Luis EmanuelBoragno, CorradoMouzourides, PetrosBorawski, AndrzejMowla, Md RakibulBorda, AnnMoya Rueda, FernandoBordel Sánchez, BorjaMoya-Albor, ErnestoBordoy, JoanMoyano Campos, Juan JoséBorecki, MichalMoysis, LazarosBorfecchia, FlavioMozerov, Mikhail G.Borgaonkar, Ravishankar BhaskarraoMozos, IoanaBorgarino, MattiaMozzanica, AldoBorges, Díbio LeandroMozzaquatro, Bruno AugustiBorghi, AlessandraMporas, IosifBorghi, FrancescaMroczyński, RobertBorghi, GuidoMróz, MarekBorich, MichaelMrugalski, MarcinBoril, HynekMrukiewicz, MateuszBoriskov, PetrMrzljak, VedranBorkowski, PiotrMu, ChaoxuBorla, OscarMu, XiaojingBormashenko, EdwardMubin, OmarBorodina, Irina A.Mück, MichaelBorodinas, SergejusMúčka, PeterBorovkov, VictorMufei, XiaoBorowska, MartaMuguruma, HitoshiBorrego, CarlosMuhamedyev, RavilBorrero Sánchez, Juan DiegoMuhammad, KhanBorri, SimoneMuhammad, ShafiqBorro, DiegoMuheidat, FadiBortoni, EdsonMühlberg, Jan TobiasBorza, DianaMuhlethaler, PaulBos, ArendMuin, SifatBosch, StephanMujica, GabrielBoschetti, GiovanniMukherjee, FalguniBose, PritamMukherjee, MithunBoshkoska, Biljana MilevaMulas, MarcoBossavit, BenoitMuldoon, ConorBosse, StefanMulla, YusufBossi, GiuliaMüller, AndreiBosso, NicolaMüller, GerhardBostater, CharlesMüller, HartmutBoström, KimMüller, IngmarBotelho, Wagner TanakaMuller, Jan-PeterBothe, Kyle M.Müller, Julian MariusBöttcher, SebastianMüller, RainerBotte, MarilisaMüller, SebastianBottin, MatteoMummolo, CarlottaBotzoris, GeorgeMumtaz, ShahidBoualouache, AbdelwahabMuncan, JelenaBoubchir, LarbiMunicio, EstebanBouchard, KevinMunir, SaidBouchner, PetrMuniraj, InbarasanBoudaden, JamilaMuniz, PabloBougas, LykourgosMunkelt, ChristophBougiatioti, KaterinaMuñoz Hernández, Germán ArdúlBoukallel, MehdiMuñoz Martinez, VictorBoukhechba, MehdiMuñoz Merino, Pedro JoséBoukhers, ZeydMuñoz Organero, MarioBoukouvalas, ZoisMuñoz, AntonioBoulogeorgos, Alexandros-Apostolos A.Munoz, RobertoBouri, MohamedMunoz, Rodrigo A. A.Bourillot, EricMunoz-Aguirre, SeverinoBourouina, TarikMuñoz-García, Miguel A.Boursianis, AchillesMuñoz-Gea, Juan PedroBousdar Ahmed, DinaMuñoz-Marí, JordiBoutaayamou, MohamedMunschy, MarcBoutayeb, HalimMunshi, TalatBoutejdar, AhmedMunster, PetrBoutry, NicolasMuntean, EdwardBouvet, Pierre-JeanMunteanu, Florentina-DanielaBouziane, BrikMuntoni, GiacomoBowden, Anton E.Munz, MichaelBozalakov, DimitarMur, AngelBozchalooi, Iman SoltaniMuraki, KatsuhikoBozcuoğlu, Asil KaanMuralikrishnan, BalaBožek, PavolMurawska-Ciałowicz, EugeniaBożek, PiotrMurawski, LechBozena, GajdzikMurayama, HideakiBozhko, Yulia Yu.Muresan, Mircea PaulBozkurt, SelimMurillo-Escobar, Miguel AngelBozomitu, Radu GabrielMurillo-Fuentes, Juan JoséBozzano, RobertoMurphy, FinbarrBrad, RemusMurphy, MauriceBrad, StelianMurroni, MaurizioBraeuer, AndreasMurtada, KhaledBraga, Guilherme S.Murugappan, KrishnanBragazzi, Nicola LuigiMusa, SarhanBraginsky, BorisMusci, MirtoBranch, PhilipMusial, JedrzejBrand, SergeMusić, JosipBrandão, AlexandreMusicant, OrenBrandão, ElsaMuslimov, EduardBrandão, Lincoln CardosoMust, IndrekBrandl, MartinMustafa, MustafaBrandolini, FilippoMustafa, Mustafa A.Brandsema, MatthewMusteata, MihaiBrandusoiu, Ionut BogdanMusuc, Adina MagdalenaBrás, SusanaMusumeci, GiuseppeBrasiello, AntonioMuszyński, ZbigniewBratashov, Daniil N.Muthanna, AmmarBratchenko, IvanMuthu Karuppan, Mohan KumarBraun, AlexanderMuthuswamy, JitBraun, SebastianMutinelli, FrancoBrauneck, JensMutlu, HaticeBravo, IgnacioMutreja, IshaBravo, TeresaMutschler, ChristopherBray, ErinMutshinda, CrispinBrcic, DavidMuttikulangara, SanathananBrcic, MarioMuttillo, MircoBreaz, Radu-EugenMuzaffar, RaheebBreed, Greg A.Muzik, JanBreguet, Jean-MarcMužík, JurajBrehm, GunnarMyasoedova, Tatiana N.Breitsamter, ChristianMyllylä, TeemuBrenker, JasonMyllymaa, SamiBrentan, Bruno MeloMylonas, AlexiosBrescia, MassimoMylonas, GeorgiosBresciani, MarianoMylonas, PhivosBressan, FedericaMystkowski, ArkadiuszBrestic, MarianMyung, HyunBretterklieber, ThomasN’Gom, MoussaBreuil, PhilippeNääs, Irenilza De AlencarBreunig, Hans GeorgNabavi, SeyedfakhreddinBrewster, LauranNabil, BachaghaBrezeanu, GheorgheNabizadeh, AzamBrida, PeterNabok, AlexeiBridgelall, RajNaccache, RafikBridle, HelenNaceri, AbdeldjallilBriggs, TiffanyNachtane, MouradBrighina, FilippoNacif, Jose Augusto M.Brincker, RuneNadaud, KevinBrinkworth, RussellNadeau, Nicholas A.Briongos, SamiraNadeem, MuhammadBriottet, XavierNaderi, EsmaeilBrisolara, Lisane Brisolara DeNaderian, AliBrito, AlissonNadolny, ZbigniewBrito, JoséNaeem, UmairBrito, PauloNaftaly, MiraBrkic Bakaric, MarijaNagai, MasayaBrkić, DejanNagamine, KuniakiBroadhurst, C. LeighNaganawa, JunichiBrocca, LucaNagano, HanatsuBrock, HeikeNagarajan, SudhagarBrockherde, WernerNagatani, NaokiBroderick, PatriciaNaghar, AzzeddinBrodie, GrahamNaghilou, AidaBrodny, JarosławNaghshvarianjahromi, MahdiBrogger, JanNagi, ŁukaszBrognara, LorenzoNagle, AnnaBronchalo, EnriqueNagles, EdgarBrooks, RandallNagy, BalázsBrosed, Francisco JavierNagy, LászlóBrouzgou, AngelikiNahab, FattaBrown, KennethNahata, SudhanshuBrown, Philip N.Nahid, Abdullah-AlBrown, Steven G.Naifar, SlimBrown, SuzanaNaik, BhavenBrown, Tim W. C.Naik, Ganesh R.Brown, TimothyNaiker, ManiBrščić, DraženNajafi, FoadBruce, Neil C.Najibi, NasserBruckman, Laura S.Najjar, MohammadBruder, StephenNakamoto, HiroyukiBruggemann, Troy SterlingNakamura, RyoheiBrundage, Cord M.Nakamura, YukoBrunet, PhilippeNakanishi, YoshitakaBrunete, AlbertoNakano, MarikoBruni, RenatoNakashima, MotomuBruni, VittoriaNakashima, ShotaBruno, FabioNakayama, YuBrunone, BrunoNakhla, SamBrus, JanNakhli Mahal, HoumanBruschini, ClaudioNakutis, ŽilvinasBrust-Mascher, IngridNaldi, MaurizioBryche, Jean-FrançoisNalepa, GrzegorzBryk, MajaNalepa, JakubBrzecka, AnnaNallal, MuthuchamyBrzostowski, KrzysztofNam, Gi PyoBu, LingbingNam, Seong-NamBu, XiongzhuNam, YunyoungBubnienė, UrtėNamiot, DmitryBuchowicz, AndrzejNanjunda, Shivananju BannurBuciakowski, MariuszNanni, JacopoBučinskas, VytautasNanni, LorisBuckley, JeffreyNanni, UmbertoBucur, BogdanNanukuttan, SreejithBudaciu, CristinaNapa, Sae-BaeBudaszewski, DanielNapier, ChrisBudeanu, MariusNapoli, AlessandroBudetta, PaoloNaqvi, Rizwan AliBudge, Scott E.Narain, SashankBudgett, DavidNaranjo, Jose EugenioBudinski, VedranNaranjo-Hernández, DavidBudvytis, IgnasNaranjo-Torres, JoséBudzan, SebastianNarayanan, BarathBudzier, HelmutNarayanan, Ram M.Buendía, FélixNarazaki, YasutakaBuffi, AliceNarbudowicz, AdamBuhot, ArnaudNardi, FernandoBui, Hoai Nam KhacNardini, GiovanniBui, Huu KienNarducci, FabioBuisseret, FabienNarkiewicz, JanuszBuiu, CatalinNarsimlu, KemsaramBujnowski, AdamNarusue, YoshiakiBujoreanu, CarmenNascimento Jr., Cairo L.Bukhari, QasimNascimento, Daniel Chagas DoBukin, OlegNascimento, MicaelBulić, PatricioNascimento, SergioBulińska-Stangrecka, HelenaNascimento, TiagoBull, GeoffNaseer, NomanBunea, Alina-CristinaNasielski, JoshuaBunge, Christian-AlexanderNasimuddin, N.Buongiorno, DomenicoNasirahmadi, AbozarBuongiorno, FabriziaNasiri, NoushinBuono, AndreaNaskar, SusmitaBuonocore, FrancescoNasrollahi, AmirBurattini, LauraNasrollahi, KamalBurch, ReubenNasser, AbbassBurdett, RobertNassiopoulou, AndroulaBurdziakowski, PawełNassour, JohnBures, MiroslavNastasa, CristinaBurgdorf, MartinNastro, AlessandroBurghardt, AndrzejNastuta, Andrei VasileBurgués, JavierNatalello, AntoninoBurlacu, AdrianNath, ChandraBurlina, ChiaraNatsuaki, RyoBurokur, Shah NawazNattanmai Parasuraman, RamviyasBurri, SamuelNattkemper, Tim WilhelmBurrows, Andrea C.Natu, RuchaBurszta-Adamiak, EwaNauber, RichardBurud, IngunnNauroze, Syed AbdullahBurwell, GregoryNaus, KrzysztofBus, James S.Nava, GabrieleBusca, BernatNava-Baro, EnriqueBusquets-Mataix, Jose V.Navarro, ElenaBustaffa, ElisaNavarro, Javier LorenzoBustin, AurelienNavarro, Stefan EscaidaBustos, AlejandroNavarro-Camba, Enrique A.Bustos-Martínez, JaimeNavas, HelenaButlewski, MarcinNavas-Delgado, IsmaelButnariu, SilviuNavickas, RomualdasButov, Oleg V.Navickiene, SandroButt, Abdul HaleemNavikas, DangirutisButt, Muhammad AliNavrátil, VáclavButtafuoco, GabrieleNawar, SaidButtazzoni, GiuliaNayak, SridharaButun, IsmailNaydenov, StefanButusov, DenisNazarahari, MiladBuzuk, MarijoNazarkevych, MariyaBuzzelli, MarcoNazarov, Andrej P.Byeon, HaewonNazarov, Dmitry MikhailovichBykhovsky, DimaNazeer, MuhammadBykov, Dmitry A.Nazemifard, NedaByoung-Hee, LeeNazir, SalmanByrne, DallanNdibanje, BruceBystroff, ChristopherNdieyira, JosephBystrov, AleksandrNeagoe, MirceaByun, HyeranNeagu, Bogdan ConstantinByun, SangjinNeagu, Bogdan-ConstantinCabaj, JoannaNečas, DavidCabaj, KrzysztofNeculoiu, DanCabaleiro, ManuelNeethirajan, SureshCaballero-Mora, Maria AngelesNeghinǎ, CătălinaCabeda, José ManuelNegrean, IuliuCabestany, JoanNehra, AjayCabral, JaderNeilsen, TraciCabral, JaydeeNeipp, CristianCabrera Martínez, AbelNejad, Ali FarokhiCabrera, HumbertoNelatury, SudarshanCabrera, Maria Eugenia (Maru)Nelli, RahulCabrera-Munoz, NestorNemes, CiprianCaccavale, MauroNémeth, Zsolt IstvánCadatal-Raduban, MarilouNeophytou, NeophytosCaesarendra, WahyuNepa, PaoloCafaro, FrancescoNepomuceno, Erivelton GeraldoCafolla, DanieleNepomuceno, ThyagoCaggianese, GiuseppeNeri, PaoloCai, GuofaNeshatvar, NazaninCai, HaipengNesterova, Irina V.Cai, HuaqingNetto, Roberto S.Cai, JunNetzelmann, UdoCai, WenyuNeu, JensCai, YapingNeumann, Patrick P.Caiani, Enrico G.Neumann, PhilippCailean, Alin-MihaiNeusypin, KonstantinCain, RyanNeužil, PavelCain, StephenNevalainen, PaavoCalabrò, FrancescoNeves, FilipeCalado, João M. F.Neves, João C.Calafate, Carlos TavaresNeves, JoséCalamanti, ChiaraNeves, Leandro A.Calaon, MatteoNewair, Emad F.Calbimonte, Jean-PaulNewe, ThomasCaldas, PauloNežerka, VáclavCaldeira, João M. L. P.Ng, JosephCalderon, CarlosNg, SebastianCalderon, JuanNgo, ChunyongCăleanu, Cătălin-DanielNgo, DatCalegari, RobertaNgo, Ha DuongCalendar, RichardNgoc Nguyen, QuangCalì, MicheleNguyen Gia, TuanCaliebe, WolfgangNguyen, BinhCaliendo, CinziaNguyen, Binh P.Caligiuri, Michael P.Nguyen, ChuongCalin, VladeanuNguyen, Dong-NhatCalinescu, IoanNguyen, Duc-HiepCalín-Sánchez, ÁngelNguyen, GiangCalixto, IacerNguyen, HoangCalle Cancho, JesúsNguyen, HungCallegari, BiancaNguyen, Huong ThuCalleja, MontserratNguyen, Huy T.Calleja-Arriaga, WilfridoNguyen, Khuong AnCallejón Leblic, María AmparoNguyen, KienCalvaresi, DavideNguyen, LinhCalveras, AnnaNguyen, Long QuangCalvillo-Arbizu, JorgeNguyen, Minh TuanCalvini, RosalbaNguyen, Ngoc HungCalvo, AngelaNguyen, NhaCalvo, DavidNguyen, PhucCalvo, HiramNguyen, Thien D.Calvo, RoqueNguyen, TrangCalvo-Fullana, MiguelNguyen, TrieuCalvo-Lobo, CésarNguyen, Trong-NguyenCalzarossa, Maria CarlaNguyen, Trong-TheCam, HasanNguyen, TrungCamara, CarmenNguyen, Trung H.Cámara-Chávez, GuillermoNguyen, TuCámara-Zapata, José MaríaNguyen, Van DungCamarena-Martinez, DavidNguyen, Viet HaCamargo, Sandro Da SilvaNguyen, VuCamas Anzueto, Jorge LuisNguyen, Xuan SonCambria, ErikNguyen-Van, TrietCamerlingo, CarloNhu-Ngoc Dao, DaoCameron, NoelNi, Chuen-FaCamiciottoli, GiannaNi, HuanCamilleri, VanessaNi, JunCamiz, SergioNiaghi, Ali RashidCampagnoli, RafaelaNiaz, Muhammad TabishCampanella, LuigiNiazi, ImranCampbell, AnthonyNiazi, Imran KhanCampbell, DanaNiccolai, AlessandroCampbell, Michael (Columbia University, USA)Nicholson, VaughanCampbell, Michael (Fort Lewis College, USA)Nico, GiovanniCampbell, Sawyer D.Nicolae, MaximilianCampeau-Lecours, AlexandreNicolas, Garcia AracilCampelo, JoséNicolay, PascalCampian, Cristina MihaelaNicolazzo, SerenaCampioni, IlariaNicolò, AndreaCampo Vázquez, CelesteNicoloiu (Stefanescu), AlexandraCampo, CelesteNicu, Ionut CristiCampo, EricNie, DongHuCampobello, GiuseppeNie, LimingCamponogara, IvanNie, LiqiangCampopiano, StefaniaNie, MengyanCampos, Alex Fabiano CortezNie, ShengCampos, Antonio Luiz Pereira De SiqueiraNie, ZedongCampos, RuiNiedbała, GniewkoCamposo Pereira, ArturNiedbalski, ZbigniewCampos-Taberner, ManuelNiederer, DanielCampuzano, SusanaNiedoba, TomaszCandefjord, StefanNiemczewska-Wojcik, MagdalenaCanders, Wolf-RüdigerNierhaus, TillCannon, KevinNieto Escámez, FranciscoCano, AlbertoNiewiadomska-Szynkiewicz, EwaCano, Maria-DoloresNiewiadomski, AdamCano-Espinosa, CarlosNifli, Artemissia-PhoebeCaño-García, ManuelNijim, MaisCanovas Martinez, RocioNikiforova, AnastasijaCantero-Chinchilla, SergioNikitas, NikolaosCantor-Cutiva, Lady CatherineNikitin, PetrCao, ChangNikodem, JanCao, DengNikodem, MaciejCao, HuiliangNikolaev, DmitryCao, JianNikolaev, KonstantinCao, JianyunNikolaidis, AthanasiosCao, JiaweiNikolaidis, SpirosCao, JiuwenNikolaidis, TheoklisCao, JunweiNikolaos, KarathanasopoulosCao, LinNikolaou, SymeonCao, LiqinNikolelis, Dimitrios P.Cao, PeiNikolic, DejanCao, PengNikulchev, EvgenyCao, SuzhiNilsson, TobiasCao, WenzhuoNimmagadda, ShastriCao, YunqiNimse, Satish BalasahebCao, ZhiguangNing, HuimingCao, ZhiqiangNing, ZhaolongČapek, JanNino, PasqualeCapineri, LorenzoNiola, VincenzoCapitão, Silvino DiasNiort, JannickCapobianco, EnricoNiri, Mona FarajiCapobianco, GiuseppeNisheva, MariaCapodaglio, AndreaNishida, YuyaCapone, SimonettaNishiguchi, HajimeCaporali, AlessandroNishihara, RyoCaporusso, NicholasNishio, MizuhoCapozzoli, LuigiNishiuchi, HiroakiCappelletti, ChantalNishiyama, AkikoCappelletti, LucaNishiyama, MichikoCappuzzello, FrancescoNissen, IvorCapra, AlessandroNistor, EleonoraCapretz, MiriamNistor, SorinCapuano, RosamariaNitta, KouichiCaputo, DomenicoNitti, MassimilianoCarabias-Orti, Julio J.Nitti, MicheleCarabin, GiovanniNitzan, ItamarCarbonaro, Carlo MariaNitzan, MeirCarbonaro, NicolaNitze, IngmarCarbone, PaoloNiu, LeiCarboni, BiagioNiu, YanboCárdenas, CésarNiu, ZhenguoCardenas-Juarez, MarcoNivedita, NiveditaCardieri, PauloNiwa, OsamuCardillo, EmanueleNjegovec, MatejCardona-morales, OscarNkenyereye, LewisCardoso, FernandoNobes, David S.Cardoso, Jorge C. S.Noble, Jeremy W.Cardoso, José RobertoNobre, Jéferson CamposCarfora, Maria FrancescaNobukawa, TeruyoshiCariou, ClaudeNocera, LucianoCariow, AleksandrNocerino, EricaCarles, GuillemNoda, Juan J.Carli, RaffaeleNoga, MarcinCarlos Fernandez-Diaz, JuanNoga, StanisławCarlos Ferreira, JoãoNoghanian, SimaCarlos-Mancilla, Miriam A.Noguchi, AtsushiCarlson, CharlesNogueira Rebelo, Rita DanielaCarmen Carnero, MaríaNogueira, MicheleCarminati, MarcoNoh, ByungjooCarmo, João PauloNoh, MyounggyuCarmona, EmilianoNoh, Yeon SikCarmona, Pedro LatorreNoh, Young-ChanCarnevale, DanielaNohama, PercyCarona, CarlosNolting, LarsCaroppo, AndreaNomikos, NikolaosCarotenuto, MarcoNorcic, GregorCaroti, GabriellaNord, NatasaCarranza, ÓscarNordin, Andrew D.Carrasco, AlejandroNordman, MaariaCarrasco, MiguelNoriega, ÁlvaroCarrascosa, FranciscoNoriega, Leocundo AguilarCarratalá, MaríaNorman, SteveCarrault, GuyNorrlöf, MikaelCarrega, AlexNorta, AlexanderCarreno, NeftaliNorth, Max M.Carreno-Luengo, HugoNosalewicz, ArturCarrier, MathieuNovák, AndrejCarrillo Calleja, Juan ManuelNovak, DomenCarro Fernandez, GermánNovak, JiriCarruthers, DavidNovak, MiroslavCarstensen, Jens MichaelNovak, NejcCarter, AshleyNovelli, AntonioCaruso, AntonioNovellino, AlessandroCaruso, GiuliaNovickij, VitalijCaruso, UgoNovikov, Arthur I.Carutasu, GeorgeNovoa, JoséCarvalho, DiegoNóvoa, X. RamónCasaccia, SaraNovosselov, IgorCasado-Hernández, IsraelNovotný, MichalCasado-Vara, RobertoNowak, MateuszCasalbore, DanieleNowak, RobertCasalino, GabriellaNowakowska-Lipiec, KatarzynaCasals, AlíciaNowakowski, ArturCasanella, RamonNowakowski, JacekCasari, PaoloNowicki, MichałCasas, OscarNowicki, Michał R.Casau, PedroNowosielski, AdamCasavola, AlessandroNoyer, Jean-CharlesCascianelli, SilviaNozato, HideakiCaseiro Rocha, Alfredo MoreiraNozzoli, FrancescoCaseiro, AlexandreNtantogian, ChristoforosCasimiro, AntónioNuchkrua, ThananaCaso, GiuseppeNunes, FernandoCasoni, MaurizioNunes, TeresaCaspary, ReinhardNúñez, AlfredoCassano, FabioNunez, ItzelCassara, PietroNuñez, NeftaliCassetti, GabrieleNúñez, PedroCassidy, John F.Nuñez-Andrés, AmparoCastagno, JeremyNúñez-Ramírez, FidelCastagnola, ElisaNuño-Maganda, Marco AurelioCastaldi, PaoloNurmi, JariCastaldo, PaoloNuster, RobertCastañeda Miranda, AlejandroNwaboh, Javis AnyangweCastaño, FernandoNwajana, Augustine O.Castejon, CristinaNwogu, IfeomaCastellano Paulis, JulenNyarko, Emmanuel KarloCastellanos Garzón, José AntonioNyberg, RogerCastelli, EricNyka, KrzysztofCastelli, GiulioNykiel, GrzegorzCastellini, PaoloO’Byrne, MichaelCastiglione, ArcangeloO’Duill, SeanCastillo, DanielO’Keefe, KyleCastillo, Jose CarlosO’Leary, RichardCastillo, OscarO’Loughlin, DeclanCastro Do Amaral, GustavoO’Mahony, ConorCastro, Cristina DeO’Neil, GlenCastro, IsabelO’Nils, MattiasCastro, Ivan D.O’Shaughnessy, DouglasCastro, José Luis AlbaO’Sullivan, DavidCastro, Maria Claudia FerrariO’Toole, MichaelCastro-Escarpulli, GracielaObland, MichaelCastro-Garcia, Juan AntonioObreja, Serban GeorgicaCastro-Méndez, AuroraObrzut, JanCasula, GiuseppeOcchipinti, LuigiCatala, AndreuOchi, MasayukiCataldo, AndreaOchieng, Washington YottoCataldo, RosellaOchoa-Brust, AlbertoCatalin, DumitrescuOchoa-Ruiz, GilbertoCatalina-Lucia, CocianuOchoa-Zezzatti, AlbertoCatani, JacopoOchowiak, MarekCatania, AlessandroOddo, Calogero M.Catania, PietroOdry, AkosCatapano, IlariaOdziemczyk, WaldemarCatelli, Danilo S.Oest, AdamCatena, RobertOfferhaus, Herman L.Catherwood, Philip A.Offermans, PeterCatini, AlexandroOgashawara, IgorCattaneo, GiuseppeOgawa, KeijiCattani, LucaOgawa, ShinpeiCaubel, Julien J.Ogawa, TakahiroCaulfield, MalcolmOggeri, ClaudioCaurin, GlaucoOgiela, LidiaCausa, FlaviaOgiela, UrszulaCausse, MickaëlOgilvie, SeanCauteruccio, FrancescoOgino, MikitoCavaco Mendes, Mário José GonçalvesOgiso, SatokiCavagnaro, MartaOgras, UmitCavalieri, Daniel CruzÖgren, MikaelCavallari, Marco RobertoOgundile, Olayinka O.Cavalli, Rosa MariaOgut, MehmetCavallo, ArmandoOh, Je HoonCavallo, EugenioOh, Jeong-HoCavallo, FilippoOh, JungsuekCavenaghi, MarcosOh, Ki YongCavoretto, PaoloOh, KwangseokCawley, PeterOh, Min SeokCazacu, Marius MihaiOh, Min-cheolCazorla, MiguelOh, SeongkeunCazzola, DarioOh, SeungminCazzolato, Mirela T.Oh, TaekeunCeburnis, DariusOh, Tong InCechowicz, RadosławOhashi, HirokiCecilia, Jose MariaÖhberg, FredrikCecilio Da Silva Jr., DiógenesOhira, Shin-IchiCecílio, JoséOhlckers, PerCedomir, MilosavlevicOhno, SeigoCelaya-Padilla, José M.Ohodnicki, PaulCelik, NumanOhsawa, RyouCellini, AntonioOhtsu, MasayasuCely, Juan S.Oikonomou, KonstantinosCennamo, NunzioOjarand, JaanCenten, PeterOjaroudi Parchin, NaserCentenaro, MarcoOjeda, Dora LuzCenteno, AnthonyOjeda, EdilbertoCenteno, Jorge Antonio SilvaOjeda, LauroCenturelli, FrancescoOjo, Mike OluwatayoCerbu, CameliaOjsteršek, RobertCercas, Francisco António BuchoOk, Jong G.Cerchione, RobertoOkada, TatsuakiCerjan, BenOkajima, JunnosukeCermak, PeterOkamoto, ShogoCernadas, EvaOkamoto, TakashiCernat, AndreeaOkarma, KrzysztofČerný, MartinOkumura, SusumuCerretti, RaffaellaOlague, GustavoCerro, GianniOlaizola, Igor GarcíaCerruto, EmanueleOland, EspenCeruti, AlessandroOlanrewaju, AyokunleCervantes, EmilioOlaru, AndreiCervantes, JairOlasupo, Tajudeen O.Cesano, FedericoOlaszek, PiotrCésar Cavalcanti Roza, VálberOlazagoitia, José LuisCesarini, AndreaOleniacz, RobertČesnulevičius, AlgimantasOlesberg, JonathonCéspedes, SandraOletic, DinkoCetin, SabriOlewnik-Kruszkowska, EwaCeyssens, FrederikOlga, DoroshenkoCha, Kyoung ChulOliva, DiegoCha, Seung HyunOliva, GabrieleCha, WonsukOlivares, JimenaCha, Young-JinOlivares, TeresaChacko, Jenu VargheseOlivares-Olivares, Pablo JoséChacón Cuberos, RamónOlive, DavidChacón Muñoz, Jesús MiguelOlive, XavierChai, DengfengOliveira Salles, MaiaraChai, JingOliveira, Andre Schneider DeChai, QuanOliveira, HelderChai, Young HoOliveira, HenriqueChaim, Marcos LordelloOliveira, HorácioChaix, Jean-FrançoisOliveira, João PedroChakrabarty, AnjanOliveira, LuísChakraborty, JoyrajOliveira, RicardoChakraborty, JoyramOliveira, RúbenChakraborty, KaushikOliveira, Sonia M.Chakravarty, ShatadruOliver, ProbstChalhoub, GérardOliver, SergioChalmers, AlanOlivera, Vicente MatellánChalyan, TatevikOlivetti, ElenaChamorro Petronacci, Cintia MicaelaOlivetti, Elena CarlottaChamoso, PabloOlivier, PierreChan, Chia-TaiOliviero, MassimoChan, Ho YinOller, JoaquimChan, King YukOlmo, AlbertoChan, PakOlmos, Antonio MartínezChan, SunneyOlofinjana, AyodeleChan, Teck KaiOlorunniji, Femi J.Chan, Yu-WeiOlsson, FredrikChander, HarishOlszewska, EwaChandran, SatheeshOltean, GabrielChandrasekaran, ArvindOlthuis, WouterChanel, CarolineOlugbara, OludayoChang, Chao-TsunOluyisola, OlumideChang, Chia-ChenOlvera Trejo, DanielChang, Chia-OuOlvera-López, ArturoChang, Chih-hung (Alex)Omboni, StefanoChang, Chih-YuanOmelina, ĽubošChang, Chih-YungOmelkov, SergeyChang, Chung-LiangOmetov, AleksandrChang, Chung-PingOmidalizarandi, MohammadChang, GuobinOmidvarborna, HamidChang, Hsing-chungOmura, YasuhisaChang, Hsiu-WenOña Simbaña, Edwin DanielChang, I. PinOnaca, AlexandruChang, Jer-MingOnat, AltanChang, JiyulOniga, FlorinChang, Kai-ChunOniga, Valeria-ErsiliaChang, Kang-MingOnireti, OluwakayodeChang, Ke-VinOno, KanjiChang, LingOno, KazuoChang, Lin-huangOnofrei, Roxana RamonaChang, Ming-ChingOo, Thant ZinChang, QingOoi, Chin HongChang, SekchinÖpik, AndresChang, SeongjuOpromolla, RobertoChang, Seung JinOrcos, LaraChang, Sheng-PoOrdaz, PatricioChang, TengfeiOrdoñez Morales, Francisco JavierChang, TonyOrengo, GiancarloChang, VictorOrešković, JasnaChang, WanjungOrgaz, Eva VargasChang, WenlongOrion, ItzhakChang, Won-DuOrlando, DaniloChang, Yao-FengOrlando, SantoChang, Yau-ZenOrlovskii, YuriiChang, Yeong-HwaOrłowska-Kowalska, TeresaChang, Yu-ChungOrobtchouk, RegisChaniotakis, Nikos A.Orosei, RobertoChannon, Robert B.Orosz, TamásChantana, JakapanOrozco Barbosa, LuisChao, How-RanOrozco, UlisesChao, YuanOrsini, AndreaChapeleau, XavierOrsini, GiovannaChapman, RyanOrsolino, RomeoChapman, SteveOrtale, RiccardoChappel, EricOrte, AngelCharalambous, CharalambosOrtega Avila, Ana BelenCharlebois, Serge A.Ortega Sarmiento, SamuelCharlot, BenoîtOrtega-Huerta, MiguelCharpenay, VictorOrtego, DiegoCharrier, JoelOrtegon, JaimeCharvat, KarelOrtenzi, LucianoCharvátová, HanaOrtgies, Dirk H.Chatterjee, AyanOrtín-Obón, MartaChatterjee, SampurnaOrtis, AlessandroChatterjee, SubhasriOrtiz, FranciscoChatterton, StevenOrtiz, JesúsChatzidakis, StylianosOrtiz-Lozano, José ÁngelChatzigiannakis, IoannisOrtner, MichaelChatzimisios, PeriklisOrton, Thomas J.Chatzitofis, AnargyrosOrtuño, José MaríaChau, Kwok-WingOrúe López, Amalia BeatrizChau, Lai-KwanOrynycz, OlgaChaudet, ClaudeOrzan, Mihai CristianChaudhary, AnkitOsaba, EnekoChaudhary, UjwalOsakada, YasukoChaudhuri, Sandeep K.Osborn, LukeChaudhury, AyanOsco, Lucas PradoChauhan, VedangOseev, AleksandrChauhan, Vedang DilipkumarOshan, TaylorChaumette, EricOshman, ChristopherChava, Rama KrishnaOshtorjani, Ehsan KianiChavanne, XavierOskarbski, JacekChávez, Guillermo CámaraOsman, ShazaliChazalon, JosephOsonga, Francis J.Che, ZhengpingOsório, Jonas H.Chęciński, JakubOssa, Luis Fernando CastilloCheema, Adnan AhmadOssandon, MiguelChehri, AbdellahOsseiran, AdamChelli, Francesco M.Oßwald, KaiCheluszka, PiotrOstasevicius, VytautasChen (Richard), LiÖsterberg, PatrikChen, AlbertOstnes, RunarChen, BaifanOstrovidov, SergeChen, BaileOstrowska, KseniaChen, BaojunOsuna-Enciso, ValentínChen, BinqiangOsypiuk, RafałChen, BoOszust, MariuszChen, Bo-HaoOszutowska-Mazurek, DorotaChen, ChanghaoOta, NobutoshiChen, Chao (China)Otálvaro, Sameul SernaChen, Chao (USA)Otamendi, Francisco JavierChen, ChaoheOtebolaku, AbayomiChen, ChaonaOterkus, SeldaChen, ChenOtero, AbrahamChen, Cheng-HuanOtero, JorgeChen, Cheng-WeiOtero, PabloChen, ChiachungOthman, AliChen, Chien-ChangOthman, ArsalanChen, Chien-ShengOtis, MartinChen, Chien-yuanOtnes, RoaldChen, Chi-HuaOton, ClaudioChen, Chin-LingOtoum, SafaChen, Chin-ShanOtremba, ZbigniewChen, Chin-TaiOtrok, HadiChen, Chi-YuanOtsuka, KeisukeChen, ChuanfaOtsuka, YuichiChen, Chun-TaOtsuka, YutaChen, DechaoOtt, PeterChen, FengOu, JianzhenChen, GuoqingOu, WeihuaChen, Hai BinOuahabi, AbdeldjalilChen, HanchiOualkadi, Ahmed ElChen, HaoOuda, MahmoudChen, HaoyaoOudre, LaurentChen, HongtianOuhyoung, MingChen, Hongyang Ouyang, HanChen, HouyangOuyang, YingkaiChen, HowardOvaliadis, KyriakosChen, Hsing-ChungOvereem, SebastiaanChen, HuifangOvseník, ĽubošChen, Hung-ShingØvstedal, OlaChen, JiagengØvsthus, KnutChen, JiahongOwda, Amani YousefChen, Jian-LianOwen, JoshuaChen, JiaweiOwerko, TomaszChen, JieOyekan, JohnChen, JienanÖzdamar, ÖzcanChen, JiucunOzer, EkinChen, Jun (China)Ozeri, ShaulChen, Jun (USA)Ozolins, OskarsChen, JunpingPaar, GerhardChen, KaiPaar, RinaldoChen, KaifeiPabst, OliverChen, Kai-ShengPaccagnella, AlessandroChen, KePacheco, FanniaChen, KongzhePacheco, João G.Chen, Liang-BiPacheco-Londoño, Leonardo C.Chen, Lien-WuPacia, Marta Z.Chen, LingPaciorek-Sadowska, JoannaChen, LinglingPacurar, ClaudiaChen, LingxinPaczos-Grzeda, EdytaChen, Lung-ChienPadalkar, MugdhaChen, MaolinPaderewski, PatriciaChen, MiPadilha Lanari Bo, AntonioChen, MinPadilla, Francisco M.Chen, MingPadilla, MoisesChen, MingzhePadua, LuisChen, Nai-HuaPadulo, JohnnyChen, Peng (Southeast University, China)Paek, JeongyeupChen, Peng (Technology of Anhui University, China)Paek, Sung WookChen, PengzhanPagán, Brianna R.Chen, Ping-HeiPagano, SergioChen, Po-YuPage, AlexChen, RanPagello, EnricoChen, Roland K.Pagiatakis, GerasimosChen, RoufeiPagliazzi, Marco.Chen, Rung-ChingPai, Ming-ChangChen, ShengyangPaiano, RobertoChen, Shen-MingPaice, AndrewChen, Shih-JuiPaidi, Santosh KumarChen, Shi-HuangPaidi, VijayChen, Shih-YuPaim, LeonardoChen, ShuisenPaiva, CassioChen, ShunyunPająk, MichałChen, Shuo-HanPajaziti, ArbnorChen, Si-TongPak, AlexeyChen, SongyuePak, SoojongChen, SumingPakos, WojciechChen, TaoPakrashi, VikramChen, Tao-HsingPakula, AnnaChen, TiPal, NabamitaChen, TianfeiPal, PinakiChen, TingguiPalacín, JordiChen, Tsung-LinPalacios-Laloy, AgustinChen, Wei (Fudan University, China)Palacz, MagdalenaChen, Wei (Xi’an University of Science and Technology, China)Palade, AndreiChen, WeihaiPalade, VasileChen, WeiminPalamà, RiccardoChen, WenPalani, HariChen, WenwenPalanisamy, SelvakumarChen, WuPalau, AnnaChen, XiangPalazzi, ValentinaChen, XiangchengPalczynska, BeataChen, XiangdongPaleari, MarcoChen, XiangpingPalego, CristianoChen, XianzhongPaleologu, ConstantinChen, XiaoliangPalermo, AmeliaChen, XiaolongPalermo, EduardoChen, XiaozhiPalestra, GiuseppeChen, XigaoPaleu, ViorelChen, XinqiangPalevicius, ArvydasChen, XiyuanPalinko, IstvanChen, XudongPalipana, SameeraChen, XueminPalis, StefanChen, YanPałka, NorbertChen, YanfeiPalladino, DomenicoChen, YangPalleschi, VinceChen, YaofeiPallotta, GiulianaChen, Yen-LinPallotta, LucaChen, Yen-weiPalm, RainerChen, Yeou-JiunnPalma, LorenzoChen, YewangPalma, Luis BritoChen, Yi-ChengPalma, MatteoChen, YifanPalma, SusanaChen, YijiaPalmer, DianeChen, YingnongPalmeri, RobertaChen, YingxinPalmerini, GiovanniChen, YinshengPalmieri, Francesco A. N.Chen, YipingPalmieri, LucaChen, YiyangPalmieri, MarcoChen, YongPålsson, BjörnChen, YouganPalumbo, AnnunziataChen, YuanliuPalumbo, DavideChen, YuePalumbo, PierpaoloChen, Yu-HsuanPalzer, StefanChen, YulinPamplona Segundo, MaurícioChen, ZengshunPampuro, NiccolòChen, ZezongPamucar, DraganChen, ZhanlongPamuła, TeresaChen, ZhePan, GuoqingChen, ZhenghuaPan, HaozhiChen, Zheng-maoPan, Jeng-ShyangChen, ZhiCongPan, Sung-BumChen, ZhigangPan, TaisongChen, ZhiminPan, WeifengChen, ZhongbiaoPan, XiaofangChen, ZongHaiPan, YiqunChen, ZuoqiPan, Yong-LeChendong, DuanPan, YunCheneler, DavidPanagiotakis, CostasCheng, Chia-ChiPanagiotopoulou, AntigoniCheng, Chun-AnPanagiotou, PericlesCheng, Fang (China)Panagopoulos, Dimitris J.Cheng, Fang (Singapore)Panagoulia, DionysiaCheng, GongPanahi, FereidounCheng, HongguangPanapakidis, IoannisCheng, Hsin-Yi KathyPanayiotis, KoutsabasisCheng, Huanyu (Larry)Panda, ManojCheng, JiePander, TomaszCheng, JingPandey, GauravCheng, JuanPandey, Om JeeCheng, JunPandey, Prem ChandraCheng, KaiPandiev, IvailoCheng, LinPandit, HemantCheng, QiangPandíțh, AnúpCheng, RuiqiPanduro, Marco A. PanduroCheng, Shyi-ChyPandya, AbhilashCheng, Wen-chiehPaneque, ManuelCheng, Wen-HsiPaneque, PilarCheng, Wood-hiPanero, ElisaCheng, XinPang, FufeiCheng, XuanPang, GuangchangCheng, YangPang, XiaodanCheng, YangchunPani, DaniloCheng, YongqiangPanitsa, MariaCheon, JiminPankiewicz, BogdanCheong, Kang HaoPankowska, MalgorzataChernov, AndreyPannek, CarolinChernyi, SergeiPannek, JürgenCheron, GuyPannone, DanieleCheskis, DimaPanowicz, RobertChessa, StefanoPantano, MariaCheung, ChichungPantazi, Xanthoula EiriniCheval, SorinPantazis, GeorgeChew, Hooi PinPanwar, MayankChi, ChengPanzardi, EnzaChi, HaoranPao, Tsang-LongChi, Sheng-WeiPaolesse, RobertoChi, Wu-ChengPaoletti, Mercedes E.Chi, ZichnegPaolicelli, FedericaChiabrando, FilibertoPaolini, ChristopherChiadighikaobi, Paschal ChimeremezePaolozzi, AntonioChiadò, AlessandroPaolucci, MichelaChiandone, MassimilianoPaone, NicolaChiang, Chen-KuoPapa, MauricioChiang, Chia-ChinPapa, UmbertoChiang, Hai-PangPapacharalampopoulos, AlexiosChiang, Kai-WeiPapadakis, AndreasChiang, Ming-HsiPapadia, GabrieleChiang, Shiuh-hua WoodPapadopoulos, BasilChiariotti, FedericoPapadopoulos, GeorgiosChiatto, MatteoPapadopoulos, Georgios Z.Chiavaioli, FrancescoPapadopoulos, NikosChiba, TakashiPapadopoulos, TheofilosChica, Jose-Maria SerranoPapageorgas, Panagiotis G.Chicea, DanPapageorgiou, Ismini EChiementin, XavierPapageorgiou, MarkosChien, Feng-TsunPapaj, JanChien, Jung HungPapakonstantinou, ApostolosChien, Ying-RenPapakostas, George A.Chierici, AndreaPapalambrou, AndreasChiesi, MartaPapalitsas, ChristosChih, MingchangPapalou, AngelikiChikurtev, DenisPaparrizos, JohnChilamkurti, NaveenPapathanassiou, Anthony N.Chilipirea, CristianPapathanassiou, ApostolosChillara, Vamshi KrishnaPapic, VladanChin, Cheng SiongPaplinski, JanuszChinappi, MauroPapoutsidakis, Michail G.Ching, Congo Tak ShingPappu, ChandraChinh, Nguyen DucPappu, Chandra S.Chinnadayyala, Somasekhar R.Parada, RaúlChinni, RosemarieParadisis, DimitriosChinnici, MartaParaforos, Dimitrios S.Chinthammit, WinyuParalič, JánChiodini, SebastianoParaoanu, Gheorghe SorinChipaux, MayeulPardàs, MontseChirayath, VedPardo, AntonioChiriyath, AlexParece, TammyChirosa Ríos, Luis JavierParedes-Madrid, LeonelChiti, FrancescoParent, GillesChiu, Chien-KuoParfenov, Vadim A.Chiu, Chi-YukParham, KiyanChiu, Chuang-YuanParia, DebadritaChiu, Nan-FuPariente, Cristóbal GarcíaChiu, Shih-WenParinov, Ivan A.Chiuri, AndreaPark, ByeongjinChiverton, JohnPark, ByungwoonChizari, HassanPark, ChansikChladek, GrzegorzPark, Dae SungChmielewski, SzymonPark, DaesungChmura, PawełPark, EunhyeokChmyrov, AndriyPark, GisuCho, ChunheePark, HaemiCho, Dang-ilPark, HangueCho, GihwanPark, HanhoonCho, HeeryonPark, Hyung-MinCho, Hyeon-YeolPark, HyunheeCho, Seong JinPark, JaehwaCho, SungHyunPark, Jae-HyeungChocholouš, PetrPark, Je-KyunChoe, Se-woonPark, JeonghunChoi, AnthonyPark, Jin GyuChoi, Cheon WonPark, JinseChoi, DukhyunPark, Jong-IlChoi, Gyu-SangPark, Jong-SeungChoi, HeungjaePark, JongwoongChoi, HojongPark, JoontaekChoi, Hyun-HoPark, Jun HuiChoi, HyunjinPark, Jung-DongChoi, Jaehyuk (Korea)Park, Jung-MinChoi, Jaehyuk (USA)Park, JunyongChoi, JeongsikPark, JuyounChoi, JongmooPark, Ki SooChoi, JongwonPark, KidongChoi, JungilPark, KihanChoi, Kyeong-KeunPark, Ki-HoChoi, Min-KookPark, KiSungChoi, Mun-TaekPark, MinChoi, OukPark, MyunghwanChoi, SangjoPark, PangunChoi, Seung-bokPark, PiljaeChoi, SujiPark, Sang MinChoi, WonjaePark, SangbaekChoi, Woo JunePark, SangjoonChoi, Woo-SeokPark, SehyunChoi, WooyeolPark, Soo-HyunChoi, Yong JunPark, Soon-YongChoi, Yong SukPark, Sung IlChoi, YosoonPark, TaeHyoungChoi, YoungjinPark, Tae-HyoungChoi, YukyungPark, Won KwangChoiński, DariuszPark, Yoo MinChoisne, JuliePark, You-JinCholewa, TomaszPark, Young JuneChomiak, TaylorParker, Stewart F.Chon, KiParmehr, Ebadat GhanbariChong, AdrianParmet, YisraelChong, PeterParra Boronat, LorenaChong, Sook-YeeParra, LorenaChong-Quero, Jesús EnriqueParra, RamonChoo, HyunwookParra, VicenteChoo, Pei LingParrein, BenoîtChoo, YoungminParres-Peredo, AlvaroChorsi, Meysam T.Parrilla Pons, MarcChorti, Arsenia (Ersi)Parrilla, LuisChortos, AlexParrington, LucyChorzepa, Mi G.Parron, JosepChou, NamsunParry, DaveChou, ShuoyanParšeliūnas, EimuntasChou, Tien-YinParteli, Eric J. R.Choudhry, Rizwan SaeedPartha Narayan, MishraChouhan, Raghuraj SinghPartila, PavolChouhan, Shailesh SinghPartsinevelos, PanagiotisChoulga, MargaritaParupudi, TejasviChowdhury, AnirbanParus, ArkadiuszChowdhury, DhimanParvez, ImtiazChowdhury, FarhanParviainen, MikkoChowdhury, JabedParziale, AntonioChowdhury, MorshedPascal, CarlosChowdhury, NiazPascarella, DomenicoChowdhury, SazzadurPascolo, PaoloChowdhury, SourangsuPascucci, SimoneChristensen, Lance E.Pasero, ErosChristofilakis, VasilisPashchenko, DmitryChristova, MagdalenaPašić, BorivojeChronopoulos, DimitriosPasinszki, TiborChronopoulos, Spyridon K.Pasluosta, CristianChrysoulas, ChristosPasolini, GianniChu, Dinh ToiPasolli, EdoardoChu, HenryPaspallis, NearchosChu, HongqingPasquale Giofrè, VincenzoChu, JinkuiPasquali, PaoloChu, Seok JaePasqualotto, RobertoChu, Shu-ChuanPasquier, JacquesChu, ZhigangPassian, AliChua, BeeleePassos, DiegoChua, Ming-YamPastor Vargas, RafaelChuang, Cheng-HungPastor, JesusChuang, Chun-HsiangPastor, José L.Chuang, Hung-YiPastorelli, StefanoChuang, Tzu-YiPastor-López, IkerChuang, YuelongPaszkiel, SzczepanChuang, Yung-ChungPaszkiewicz, AndrzejChudnovsky, AlexandraPáta, PetrChui, Kwok TaiPatalas-Maliszewska, JustynaChun, HyunchaePatchay, SandhiranChun, Pang-joPatel, Bhavik AnilChunchuzov, IgorPatel, SaroshChung, Chih-ChungPatela, SergiuszChung, DeborahPatelarou, AthinaChung, Hyun-JoongPaternain, DanielChung, JaehakPaterno, Leonardo GiordanoChung, Jin-GyunPathak, SarthakChung, KyungYongPatil, Ashok KumarChung, MinyoungPatil, DevendraChung, Sang-HwaPatil, Samadhan BhaulalChung, Sheng-HengPatil, UjwalkumarChung, SungwonPatooghy, AhmadChung, Tein-YawPatra, SubirChung, Vincent PeyPătrașcu, MonicaChung, WoojinPatriarchi, TommasoChung, Yong-JooPatricio, Madaín PérezChung, YongwhaPatrick, ErinChung, Young MoPatroi, DeliaChuo, Li-XuanPatrone, FabioChvojka, PetrPatterson, Albert E.Chybicki, AndrzejPatterson, JenniferChybowski, LeszekPatwardhan, AmolCiaburro, GiuseppePau, GiovanniCiaccheri, LeonardoPaudel, BasantaCianca, ErnestinaPaugam, RonanCiatto, GiovanniPauk, JolantaCicchi, StefanoPaul, AnandCicciù, MarcoPaul, AndreaCiccone, MarcoPaul, AnirbanCicirelli, FrancoPauliukaite, RasaCiecholewski, MarcinPaulo, Joao RuivoCieplak, TomaszPavaloaia, Vasile DanielCieślik, SławomirPavan, Esteban EnriqueCieśliński, Jan L.Pavan, GianniCięszczyk, SławomirPavel, Octavian DumitruCiftyürek, EnginPavelka, KarelCifuentes, Carlos A.Pavesi, MauraCilfone, AntonioPavić, LukaCimalla, VolkerPavlidis, George P.Cinar, EyupPavlov, Ilya N.Cincotta, StefaniePavlyuk, DmitryCindemir, UmutPaw, Yew ChaiCintia, PaoloPaweł, DymoraCioni, StefanoPawlik, PawelCiorap, RaduPawłowicz, BartoszCiotirnae, PetricaPawłowski, EligiuszČiplys, DaumantasPawson, DavidCiria, MiguelPayá, LuisCísar, MiroslavPaynter, IanCisotto, GiuliaPayo, IsmaelCiuc, MihaiPayrebrune, KristinCiuk, TymoteuszPaz, IgorCiuonzo, DomenicoPazdera, LubošCiupitu, LiviuPazderski, DariuszCivera, MarcoPazera, MarcinCivitarese, GabrielePazhamalai, ParthibanCivit-Balcells, AntonPaziewski, JacekČížek, MartinPazzi, VeronicaClare, Richard MichaelPearce, JonathanClark, RossPeces, César BenaventeClarke, Keith C.Pecoraro, GiovanniClaro, Daniela B.Pecorella, TommasoClaudi, RiccardoPecqueur, SébastienClaudino, JoãoPediaditis, MatthewClaudio, SavaglioPedreño, Jose NavarroClaudiu Radu, PoznaPedrero González, AntonioClaver, Jose M.Pedrero, MariaClavijo, MiguelPedrosa, Eurico FarinhaClemens, SheilaPedrosa, PedroClement, PierrickPeer, PeterClement, YuenPegorini, ViníciusClemente, CarminePeham, ChristianClemente, Carmine StefanoPei, DanClemente, Filipe ManuelPei, WeihuaClemente, FrancescoPei, ZingwayClemente-Suarez, Vincente JavierPeidro, AdrianClementi, FrancescoPeila, RobertaClemmensen, TorkilPeinado, AlbertoClermont, Christian A.Peiner, ErwinClevenger, Caroline M.Peinl, RenéClose, GaelPeixoto De Almeida, MiguelCluitmans, Pierre J. M.Pejcinovic, BranimirCobb, Adam C.Pejic Bach, MirjanaCoca, EugenPekárek, JanCoccia, MarioPeláez, José A.Cocco, DanielePeláez-Moreno, CarmenCocconcelli, MarcoPelc, MariuszCochran, SandyPeleato, BorjaCochrane, CédricPelevin, Vadim V.Cocianu, Cătălina-LuciaPellas, NikolaosCoclite, Anna MariaPellegrini, GaiaCocuzza, SilvioPellenz, Marcelo EduardoCodetta Raiteri, DanielePelletier, Mathew G.Coelho, Aurélio Luiz MagalhãesPelliccia, RiccardoCoelho, Denis A.Pelosi, GiuseppeCoelho, JoãoPelton, Joseph N.Coelho, João M.P.Peluso, ValentinoCoelho, João PintoPemberton, RoyCoelho, LuisPeña Pitarch, EstebanCoelho, Luís CarlosPeña, AraceliCoelho, Vitor N.Peña, DexmontCohen, AsafPeña, M. TeresaCohen, RamiPendar, HodjatCointault, FrédéricPeng, ChangCola, GuglielmoPeng, Chao-ChungColbertaldo, PaoloPeng, ChuangCole, Jared HPeng, Dao-LiColeine, ClaudiaPeng, DengfengColella, RiccardoPeng, DiColes, NeilPeng, GaoliangColin, ElizabethPeng, HaijunColla, Eugene VPeng, JianqingColla, ValentinaPeng, JingjingCollart-Dutilleul, Pierre-YvesPeng, Kang-ChunCollen, AnastasijaPeng, LinningCollin, EddyPeng, PinganCollmann, RalphPeng, SiyuanColl-Perales, BaldomeroPeng, XiColombo, ClaudioPeng, XiaojiangColosi, FrancescaPeng, YahuiColton, Jonathan S.Peng, YepingColuccia, AngeloPeng, YongColusso, ElenaPeng, YuhuaiComai, SaraPeng, YuxinComeau, FrankPeng, ZhengyuComesaña, PedroPeng, ZhenmingComfort, PaulPenichet-Tomas, AlfonsoComisso, MassimilianoPennazza, GiorgioComite, DavidePennino, DiegoComparetti, AntonioPentiuc, Stefan GheorgheConceição Junior, Pedro OliveiraPepe, AntonioConceição, RaquelPepe, MassimilianoCondino, SaraPeppas, KostasConfais, BastienPeppel, TimConforti, MassimoPeral, JesusCongedo, MarcoPerazzo, PericleConnolly, JamesPerchoux, JulienConsolo, GiancarloPercoco, GianlucaConson, MassimilianoPerdikis, SerafeimConstantin, Gabriel-AlexandruPerea Moreno, Alberto JesúsConstantin, NistorPerego, PaoloConstantino, CosimoPereira De Figueiredo, Felipe AugustoConte, DavidePereira, Ana CristinaConteduca, DonatoPereira, AntónioContini, DanielePereira, Elena M. HernándezContreras Castillo, JuanPereira, Ernesto C.Conversi, DavidPereira, Fabio HenriqueConvertino, MatteoPereira, JoseCooley, Christopher G.Pereira, Paulo RogerioCoombes, MatthewPereira, RubenCooney, CiaranPereira, TâniaCooper, RobinPereira, TelmoCooper, RosemariePereiro, JuanČopič, MartinPérennou, AndréCoppola, ClaudioPerera, AsankaCoppola, GiuseppePerera, KithsiriCoppola, SaraPeres, António M.Coraddu, AndreaPeres, EmanuelCoratella, GiuseppePeres, Henrique E. M.Corazza, Maria VittoriaPerestrelo, RosaCorbari, ChiaraPeretto, LorenzoCorbin, Joel C.Pereverzyev, SergeiCorcoran, PadraigPérez Arribas, FranciscoCorcoran, PeterPérez De Prado, Rocío JosefinaCorcos, Daniel M.Perez De Vargas Sansalvador, IsabelCorcovilos, Theodore A.Perez Herrera, Rosa AnaCordeiro, CarlosPérez Jiménez, RafaelCordella, FrancescaPérez Menéndez, Ramón JoséCordero Fuertes, Juan AntonioPerez Ruiz, ManuelCordero, JorgePérez Trujillo, Francisco JavierCoria, Luis N.Pérez, Alfredo J.Corigliano, AlbertoPerez, HuseinCorke, PeterPérez, Isidro A.Cornelis, JanPerez, JoaquinCoronato, AntonioPerez, JoshueCoros, MariaPerez, Marco AntonioČorović, SelmaPerez, Mauricio DavidCorradi, AntonioPerez-Acebo, HeribertoCorradi, NicolaPérez-Bueno, MarisaCorrado, RindonePerez-delHoyo, RaquelCorreia, AmericoPérez-Escudero, AlfonsoCorreia, LuísPérez-González, AntonioCorreia, MiguelPérez-Higueras, NoéCorreia, Paulo LobatoPérez-López, CarlosCorreia, PedroPérez-Martín, EnriqueCorrigan, Damion K.Pérez-Meana, Héctor ManuelCorriou, Jean-PierrePerez-Navarro, AntoniCorsaro, CarmeloPerez-Ramirez, CarlosCorsi, CarolPérez-Rey, IgnacioCorsi, CristianaPérez-Rodríguez, FernandoCorso, GilbertoPérez-Suay, AdrianCortés, Araceli GonzálezPérez-Torres, RafaelCorti, FabioPergola, NicolaCorti, MartinaPeri, DanieleCorves, BurkhardPeriasamy, Arun PrakashCosco, FrancescoPérigo, Elio AlbertoCosentino, AntoninoPerikos, IsidorosCosoli, GloriaPerilli, StefanoCosoli, SimonePeriyasamy, Aravin PrinceCossel, KevinPernau, Hans FridtjofCosta Ramos, DanielPernice, RiccardoCosta, ÁngelPerogamvros, NikolaosCosta, AnnamariaPerotoni, Marcelo BenderCosta, CorradoPerpetuini, DavidCosta, Daniel G.Perpiñan, GilbertoCosta, ErnanePerrey, StéphaneCosta, FilippoPerri, StefaniaCosta, LuisPerrier, PierreCosta, MadalenaPerrochon, AnaickCosta, Manuel Filipe Pereira Cunha MartinsPerrone, DanieleCosta, Mário JorgePerroy, Ryan L.Costa, MiguelPerry, PhilipCosta, RicardoPerry, StuartCosta, SusanaPerš, JanezCosta, YandrePersano, AnnaCostache, RomulusPersaud, KrishnaCostantini, ElisabettaPersichetti, LucaCostantino, DomenicaPersijn, StefanCostantino, FrancescoPersson, MikaelCostanzo, AntonioPertegal-Felices, María LuisaCostanzo, SandraPertile, MarcoCosta-Rama, EstefaníaPervaiz, HarisCostelha, HugoPesant, GillesCostescu, CristinaPescapè, AntonioCotfas, Liviu-AdrianPescaru, DanCotfas, PetruPesci, AriannaCotsovos, Demetrios M.Pescini, ElisaCouce, María D.Pescosolido, LoretoCouris, SteliosPesek, MatevžCoutrot, AntoinePessanha Santos, NunoCovatariu, GabrielaPessoa, RodrigoCoviello, GiuseppePetcu, DanaCovington, JamesPeter, LukášCozzolino, DanielPeter, SteffenCozzolino, MarilenaPeterka, JozefCraciun, Eduard-MariusPeterman, WendyCraciunescu, TeddyPetiot, Jean-FrancoisCragg, PeterPetit, NicolasCraiger, John PhilipPetkie, DouglasCraus, MiticaPetko, MaciejCraven, Michael P.Petošić, AntonioCredi, CaterinaPetracca, MartinaCreedon, Sile A.Petrakis, EleftheriosCrenganis, MihaiPetrarca, MaurizioCrenna, FrancescoPetrariu, AdrianCrescentini, MarcoPetrellis, NikosCrescenzi, RoccoPetrescu, CatalinCrescitelli, AlessioPetrescu, LiviaCrespo Mariño, Juan LuisPetrescu, LucianCrétual, ArmelPetric, TadejCrezee, HansPetricevic, Slobodan J.Criado, JavierPetrila, IulianCrippa, PaoloPetritoli, EnricoCrisan-Vida, Mihaela MarcellaPetronijevic, EmilijaCristani, MatteoPetroșanu, Dana-MihaelaCristea, CeciliaPetrov, DmitryCristea, Mihaela DanaPetrov, PeterCristian, GravaPetrov, VictorCristiano, ChiaraPetrov, Victor V.Cristina, Gaitan NicoletaPetrunin, IvanCritello, Davide C.Petsa, ElliCrivello, AntoninoPettersson, AndersCroce, Anna CletaPetti, LuisaCroce, DanielePetutschnig, AndreasCrooks, GeorgePexa, MartinCrussiere, MatthieuPeyman, AzadehCruvinel, Paulo E.Peyrodie, LaurentCruz, FranciscoPezoldt, JörgCruz, GonçaloPezzutto, SimonCruz, Homero ToralPfau, ThiloCruz, Jose LuisPfeiffer, CarlosCruz, TiagoPfeiffer, PierreCruz-Vega, IsraelPfitzner, ChristianCruz-Villar, Carlos-AlbertoPfreundt, Franz-JosefCsapó, EditPham, CongDucCsató, LehelPham, GiaoCuan-Urquizo, EnriquePham, Khoa DangCuate, OliverPham, Tuyen DanhCucci, CostanzaPhan, Hoang-PhuongCuccoli, FabrizioPhan, Manh-HuongCuckov, FilipPhrampus, Benjamin J.Cucu, HoriaPiacentini, Maria FrancescaCuesta Gómez, AliciaPiamrat, KandarajCuesta, EduardoPiana, EdoardoCuesta, FedericoPiancastelli, LucaCueva, Héctor HerreroPianini, DaniloCuevas De La Rosa, FranciscoPiano, MartinaCuevas-Covarrubias, SergioPiao, XianjiCuevas-Martínez, Juan CarlosPiasecki, AdamCugurullo, FedericoPiasecki, MichałCui, FeiyunPiasecki, TomaszCui, GePicchio, RodolfoCui, HuaqingPiccinni, GiovanniCui, JiashanPiccoli, IlariaCui, JiePicerno, PietroCui, JinhuaPichiorri, FlorianaCui, JiwenPiciarelli, ClaudioCui, JunjiaPicó Vila, RubénCui, LaizhongPicone, DeanCui, PinchenPicone, MarcoCui, QingyuPidanič, JanCui, WenyuanPidaparti, RamanaCui, YaoyaoPiekarczyk, JanCui, YongjiePiekarczyk, MarcinCui, YuPielichowski, KrzysztofCui, ZhengPielli, ChiaraCui, ZhichengPiepmeier, JeffCui, ZhihuaPieraccini, MassimilianoCui, ZhisongPierdicca, RobertoCui, ZiqiangPierleoni, PaolaCui, ZongyongPierucci, LauraCuiñas, IñigoPieruccini-Faria, FredericoCulshaw, BrianPietrabissa, AntonioCunha, Ana Paula Martins Do AmaralPietropolli Charmet, AndreaCunis, TorbjørnPietrosemoli, ErmannoCuocolo, RenatoPietruszewska, WiolettaCupec, RobertPighills, AlisonCuperlier, NicolasPike, MatthewCupiał, MichałPikhart, MarcelCuriac, Daniel-IoanPikhay, EvgenyCurrenti, GildaPikridas, MichaelCurtis, PeterPilan, LuisaCusidó, JordiPilarska, AgnieszkaCutolo, FabrizioPiłat, AdamCveticanin, LivijaPilat, Adam KrzysztofĆwiąkała, PawełPilat, ZbigniewĆwiklak, JanuszPilecki, ZenonCyr, FredericPilipović, AnaCzajewski, WitoldPilla, FrancescoCzapiewska, AgnieszkaPilla, VivianeCzapla-Myers, JeffPiltan, FarzinCzaplewski, KrzysztofPimenov, DanilCzapp, StanislawPimenta, Tales CleberCzerw, KatarzynaPin, LvCzigány, TiborPina, PedroCzyżewski, AndrzejPinacho Davidson, Pedro PabloD’Acierno, LucaPinardi, StefanoD’Acquisto, LeonardoPing, JianfengD’Acunto, MarioPing, YuanD’Adamo, IdianoPinhasi, YosefD’Addona, Doriana MarilenaPinho, BrunoD’Alessandro, AntonellaPinho, MarceloD’Alessandro, AntoninoPinho, PedroD’Alonzo, MarcoPinho, Tatiana M.D’Alvia, LivioPini, MarcoD’Amato, EgidioPinkas, IddoD’Amato, RosariaPino, EstebanD’Amours, ClaudePino, Esteban J.D’Andrea, AndreaPino-Ortega, JoséD’Andrea, AntonioPintelas, PanagiotisD’andrea, CristianoPintelas, Panagiotis E.D’Andrea, VitoPinto, AdrianoD’Andreagiovanni, FabioPinto, Alex Sandro RoschildtD’Angelo, GianniPinto, Marina Marques Da Silva CabralD’Aniello, GiuseppePinto, PedroD’Anna, EdoardoPinto, SaraD’Arco, MauroPintore, GiovanniD’Aurelio, RobertaPintus, RuggeroD’Auria, AnnaPiorecky, MarekD’Emilia, GiulioPiorkowski, AdamD’Orazio, AntonellaPiotr Kucner, TomaszD’Urso, GuidoPiotr, ArabasDa Costa Cordeiro, Weverton LuisPiotrowski, PawełDa Costa Neves, Carlos GuilhermePiotto, MassimoDa Costa, CesarPiqueras, PedroDa Cunha, Felipe DomingosPiracci, Emilio GiuseppeDa Ronch, AndreaPirbhulal, SandeepDa Silva Camargo, SandroPirc, EvaDa Silva Cruz, Luis AlbertoPires De Lima, RafaelDa Silva E Silva, Francisco JoséPires, IvanDa Silva, BrunoPires, Ivan MiguelDa Silva, José MachadoPirim, HarunDa Silva, Juarez BentoPirinen, PekkaDa Silva, Luís PintoPirinoli, PaolaDa Silva, Mario MarquesPiro, BenoîtDa Silveira Petruci, João FlávioPiroddi, LucaDąbkowska, IwonaPirogova, ElenaDabove, PaoloPirola, FabianaDabrowski, Pawel S.Pirotti, FrancescoDabrowski, PiotrPirouzpanah, SahandDadic, MartinPisana, SimoneDaffara, ClaudiaPisani, MarcoDafonte, CarlosPishchalnikov, Roman Y.Daga, Alessandro PaoloPiskur, PawełDahal, KeshavPisla, DoinaDahl, MattiasPislaru-Danescu, LucianDahlhausen, MatthewPissadakis, StavrosDai, BaiqingPissard-Gibollet, RogerDai, BoyiPíštěk, VáclavDai, HaipengPistofidis, PetrosDai, Hong-NingPistori, HemersonDai, HoudePitalúa-Díaz, NunDai, JiguoPitas, Charalampos N.Dai, JunhuPiteľ, JánDai, KerenPitkäaho, TomiDai, WeiPitombeira-Neto, Anselmo RamalhoDai, ZhengfeiPitropakis, NickDaigle, Courtney LyndPitruzzello, GiampaoloDak, PiyushPitti, Rostand MoutouDalaklis, DimitriosPittino, FedericoDalgleish, MatPiuzzi, EmanueleDalin, ZhouPivarčiová, ElenaDalkiranis, Gustavo GonçalvesPiyathilaka, LasithaDall’Acqua, StefanoPizarro, FranciscoDalla Betta, Gian-FrancoPłaczek, BartłomiejDalla Vedova, Matteo D. L.Placzek, MarekDalmedico, NicolasPlana, Josep QuintanaDalverny, OlivierPlantard, PierreDamasceno, Flavio AlvesPlasqui, GuyDamaševičius, RobertasPlatero, CarlosDamborská, AlenaPlatero, Carlos A.Danaher, TimPlathottam, Siby JoseDaneault, Jean-FrancoisPlatil, AntoninDanel, RomanPławiak, PawełDanescu, RaduPlaza, PedroDanezis, ChrisPlechawska-Wójcik, MałgorzataDang Hoa, TranPleşca, AdrianDang, JiPlesinger, FilipDang, Thanh DucPletersek, AntonDang, VinhPletl, SzilveszterDani, SamirPlets, DavidDaniel Israeli-Korn, SimonPleva, MatúšDaniela, LindaPlikusiene, IevaDaniele, MichaelPliquett, UweDanielli, AmosPljonkin, AntonDanion, FredericPłotka-Wasylka, JustynaDanklmayer, AndreasPlotnikov, LeonidDannenberg, RogerPlovie, BartDanner, MartinPluciński, JerzyDantan, AurélienPlusquellic, David F.Dantas, Mario A. R.Plusquellic, JimDante, AlexPlutino, Maria RosariaDao, Van-DuongPlyusnin, Victor F.Dapino, MarceloPnevmatikos, NikosDarabant, Adrian SergiuPôças, IsabelDardano, PrincipiaPodd, Frank J.W.Daróczy, BálintPodgorelec, BlažDa-Ronch, AndreaPodgursky, VitaliDarrozes, JoséPodhorski, AdamDarsena, DonatellaPodnar, SimonDaruwalla, AnoshPodobnik, JanezDarvann, TronPodsiadły, RadosławDarvishi, MehdiPoenar, DanielDas Choudhury, SrutiPohanka, MiroslavDas, AbhishekPoisel, HansDas, AnshumanPokorny, Florian B.Das, ManoharPokorný, PetrDas, NarottamPolajžer, BoštjanDas, RupamPolańczyk, AndrzejDas, SangitaPołap, DawidDasaklis, Thomas K.Polatidis, NikolaosDasanna, Anil K.Polechoński, JacekDashtipour, KiaPolese, MicheleDasygenis, MinasPoletkin, KirillDatta, RahulPoletto, LucaDatta, RupsaPoli, GiulianoDau, Van ThanhPoliks, MarkDaunys, GintautasPolitano, Grazia GiuseppinaDavalli, AngeloPollari, FrancescoDavarpanah, AfshinPolley, NabarunDavid C., BurnettPolo, JesusDavid, JafrancescoPolo-Parada, LuisDavid, Llopis-CastellóPoltorak, LukaszDavid, SergioPolvara, RiccardoDavid, Vlad LaurentiuPolykretis, ChristosDavies, JenniferPölzl, GerhardDavila, AbundioPomar, JesúsDavila, KennyPomares, SaúlDavino, DanielePomati, FrancescoDavis, FrankPommer, ChristianDavoli, LucaPompe, TiloDavoudi, MasoumePompilli, AnnamariaDavoust, AlanPonce, HiramDawidowski, WojciechPoncet, Aurelie M.Dawn, ArnabPonchak, George E.Dawson, JeremyPonciano, Jean-JacquesDe Aguiar, Claudinei RodriguesPonikiewski, TomaszDe Almeida, Jose ManuelPoniszewska-Maranda, AnetaDe Andrés Rubio, Ana IsabelPonomarev, EugeneDe Angelis, AlessioPonomaryov, VolodymyrDe Araujo, Arthur Luiz AlvesPons, MiquelDe Benedetto, EgidioPons, PatrickDe Berardis, DomenicoPontes, Thiago BessaDe Biagi, ValerioPontikos, NikolasDe Blas, Clara SimonPontin, AntonioDe Bonis, AngelaPontual Falcão, TacianaDe Carli, LorenzoPoonthottathil, NavaneethDe Carvalho, MamedePoór, PeterDe Carvalho, Rogerio AtemPooranian, ZahraDe Castro, Bruno AlbuquerquePop, ClaudiaDe Castro, Miguel FranklinPop, Cristina BiancaDe Cesare, GiampieroPop, Florin V.De Cumis, Mario SicilianiPop, Horia F.De Dood, Michiel J.Pop, Ion OctavianDe Farias, Claudio M.Pop, Petrică C.De Farias, Claudio MiceliPopa, BogdanDe Felipe Mesquida, DavidPopa, Cicerone LaurentiuDe Figueiredo, Felipe A. P.Popa, Dan-CristianDe Figueiredo, Felipe Augusto PereiraPopa, DanielDe França, Elvis JoacirPopa, Gabriel NicolaeDe Freitas Nascimento, LuanaPopa, TraianDe Freitas, Edison PignatonPop-Calimanu, Ioana MonicaDe Freitas, Elisabete FragaPope, Gunnar C.De Grande, RobsonPopelka, StanislavDe Grande, Robson E.Popescu, DanDe Jesus Pereira, António ManuelPopescu, Daniela-ElenaDe La Cruz Sánchez, ErnestoPopescu, Ionel CatalinDe La Fuente, Jesús GrajalPopescu, NirvanaDe La Fuente, RaúlPopescu, Theodor D.De La Iglesia, Daniel H.Popescu, VasilicaDe La Llana Calvo, ÁlvaroPopov, AntonDe La Morena, JoaquinPopov, SergeiDe La O Serna, José AntonioPopov, VladimirDe La Paz, FélixPoreh, DavodDe La Rica, RobertoPortela, FilipeDe La Sen, ManuelPorter, EmilyDe La Torre Juarez, ManuelPortesi, ChiaraDe La Torre, Gabriel G.Portillo Rodríguez, OtnielDe Lacalle, Luis N. LópezPortnoy, SigalDe Las Heras Rosas, CarlosPorto, Simona Maria CarmelaDe Los Reyes, RaquelPorwik, PiotrDe Luca, Anna ChiaraPorzio, AlbertoDe Macedo Mourelle, LuizaPosada-Quintero, Hugo F.De Maria, GiuseppePossan, EdnaDe Medeiros, RicardoPostnikov, EugeneDe Melo, CelsoPostolache, OctavianDe Miguel, SilviaPotamitis, IliasDe Mingo López, Luis FernandoPotha, NektariaDe Miras, JérômePotortì, FrancescoDe Morais, Wagner OuriquePotosnak, Mark J.De Moura, JoaquimPotter, MarkDe Müllenheim, Pierre-YvesPottier, AntonyDe Nalda, RebecaPotukuchi, Praveen KumarDe Napoli, LuigiPotyrailo, RadislavDe Oliveira Junior, MarcosPouliakis, AbrahamDe Oliveira Moraes, AlisonPouliopoulos, AntonisDe Oliveira, Aline MacedoPoulopoulou, IoannaDe Oliveira, Helinando PequenoPoulose, AlwinDe Oliveira, Leise KelliPound, MichaelDe Oliveira, Mario AndersonPour, Amin BeiranvandDe Oliveira, Paulo RenatoPourpanah, FarhadDe Oliveira, Thiago E. AlvesPoursanidis, DimitrisDe Oña, JuanPourzolfaghar, ZohrehDe Pace, FrancescoPovolotskiy, Alexey V.De Pascale, FrancescoPowis, TerryDe Poorter, EliPoza-Lujan, Jose-LuisDe Regis, MichelePozdnyakov, DmitryDe Sanctis, MauroPozo, FrancescDe Santis, MichelePozzebon, AlessandroDe Simone, Marco ClaudioPrabhakar, AmlenduDe Simone, ValentinaPrabhu, RadhakrishnaDe Sousa Jr., Rafael T.Prabowo, Briliant AdhiDe Sousa Junior, Vicente AngeloPrada-Delgado, Miguel ÁngelDe Sousa Meneses, DomingosPradeep, BuddharajuDe Sousa, Frederico B.Prades, DanielDe Souza Gil, EricPraeg, NadineDe Souza, Éfren LopesPraher, BernhardDe Stefano, LucaPrajzler, VaclavDe Vargas Sansalvador, Isabel M. PerezPrakht, VladimirDe Vasconcelos, Elder AlpesPranggono, BernardiDe Vecchis, RenatoPrapotnik Brdnik, AnitaDe Venuto, DanielaPrasad, AbhinavDe Vito, SaverioPrasad, Dilip K.De Vries, Wiebe H.K.Prasad, GauthamDe Wilde, PieterPrasad, MukeshDe, SuparnaPrasad, ShaliniDeaconescu, Andrea IPrashant, KumarDeal, JoshuaPrates, GonçaloDeans, CameronPrati, RonaldoDebaene, GuillaumePrats Boluda, GemaDebeir, OlivierPrause, ChristianDebliquy, MarcPrecup, Radu-EmilDebono, Carl JamesPredic, BratislavDecarli, NicolòPreethichandra, Daluwathu Mulla GamageDechouniotis, DimitrisPrefasi, EnriqueDefay, EmmanuelPrehofer, ChristianDegli-Esposti, SaraPreiner, ReinholdDehais, FrédéricPrelipceanu, MariusDehghani, MiladPremachandra, ChinthakaDehghan-Niri, EhsanPremasiri, RanjithDehoux, ThomasPresotto, LucaDei, MichelePreu, SaschaDeinzer, FrankPreußler, StefanDekavalla, MariaPreuveneers, DavyDel Core, GiuseppePreviati, MaurizioDel Giudice, FrancescoPribičević, BoškoDel Hougne, PhilippPřibil, JiříDel Pizzo, SilvioPřibyl, JanDel Río Celestino, MercedesPrice, OwenDel Rio, Marta SylviaPrice, PatriciaDel Soldato, MatteoPricop, EmilDel Sordo, StefanoPriede, JānisDel Val, LaraPriefer, RonnyDel Valle Villalonga, LauraPriego Quesada, Jose IgnacioDel Villar, IgnacioPrieto González, LisardoDela Calle Herrero, Francisco JavierPrieto Guerrero, AlfonsoDelabrida, SaulPrieto, Juan F.Delahoche, LaurentPrieto, Juan LuisDelaney, DeclanPrieto-Castrillo, FranciscoDelaney, Keith B.Prijić, AnetaDelebarre, ChristophePrimak, SergueiDelegido Gómez, JesúsPrimatesta, StefanoDelgado Santander, RicardoPriniotakis, GeorgiosDelgado, CarmenPrins, Maarten R.Delgado, RafaelPrior, RuiDelgado, Sergio NogalesProcek, MarcinDelia-Alexandrina, MitreaProchazka, AlesDelibasis, Konstantinos K.Prochazka, DavidDelić, VladoPróchniewicz, DominikDelicato, FlaviaProchor, PiotrDelichatsios, Michael A.Proença, Pedro F.Delisle Rodriguez, DenisProkhorov, Mikhail EvgenevichDell’Olio, FrancescoProto, AntoninoDelmonico, LucasProux, OlivierDe-los-Santos-Álvarez, NoemíProvenzano, Frank A.Delot, ThierryProwell, StacyDel-Valle-Soto, CarolinaPrsic, DraganDemartini, ClaudioPrudente, Victor Hugo RohdenDementyev, ArtemPrudenzano, FrancescoDemertzis, KonstantinosPruneanu, Stela-MariaDemestichas, KonstantinosPrus, BarbaraDeming, YangPruszyńska-Karbownik, EmiliaDemrozi, FlorencPryamikov, AndreyDemyanov, VladislavPryss, RüdigerDenape, JeanPrzyborski, MarekDénes, AttilaPrzybyło, JaromirDeng, FangPrzystupa, KrzysztofDeng, JifengPsannis, KonstantinosDeng, JunjingPsarrou, AlexandraDeng, MingxiPsomopoulos, Constantinos S.Deng, QihongPsychalinos, CostasDeng, QiliangPsychas, DimitriosDeng, WeiliPtak, MariuszDeng, WuPu, CongDeng, XianjunPu, JiangboDeng, XiaogangPu, ZiyuanDeng, XiaoliPucci, RitaDeng, XinyangPuel, GuillaumeDeng, YangPueo, BasilioDeng, YingbinPuertas, EnriqueDeng, YunbinPuiatti, AlessandroDeng, ZhaohongPuig, VicençDeng, ZhiguoPuigt, MatthieuDeng, ZhihongPuiu, MihaelaDenisenko, Valery V.Pujari, AmitDenisiewicz, ArkadiuszPukalchik, MariiaDenisov, Ivan A.Pukanská, KatarínaDeniziak, StanisławPulay, AlanaDenkenberger, DavidPuli, Venkata SreenivasDenoual, MatthieuPuliafito, AntonioDentamaro, VincenzoPulido Fernández, ManuelDenti, EnricoPullano, Salvatore A.Denzler, JoachimPuniya, Bhanwar LalDeo, Randhir P.Purcar, VioletaDeo, RavinPuschita, EmanuelDeore, Prashant S.Pusnik, MajaDepciuch, JoannaPutilov, ArcadyDepiante, MarcoPutrino, GinoDepolli, MatjazPuttinger, StefanDer Wiesche, Stefan AusPyo, SukhoonDerawi, Mohammad OmarPyra, JózefDerbel, FaouziPyrchla, JerzyDerlatka, MarcinPyromalis, DimitriosDerlukiewicz, DamianPyszko, RenéDerrode, StéphanePytharouli, StellaDertimanis, Vasilis K.Pytka, JarosławDerugo, PiotrQamar, ZeeshanDesai, JaydipQasha, RawaaDeserno, Thomas M.Qazi, AbroonDeshpande, Rucha A.Qi, GuanqiuDesmaële, DenisQi, HongDesmulliez, MarcQi, JianboDesprats, ThierryQi, JieDetrembleur, ChristineQi, KunlunDeufemia, VincenzoQi, LianyongDeveci, MuhammetQi, LongDevidson, SergioQi, XiaojuanDevost, DominicQi, XiaozhiDevoto, StefanoQi, YanfeiDevy, JeromeQi, YuankaiDewan, AshrafQian, DianweiDewey, Rebecca SusanQian, JianqiangDewy, RichardQian, Jin-yuanDey, MaitreyeeQian, KunDey, NilanjanQian, WeiDey, ShuvashisQian, ZhengDezan, CatherineQiao, JingDezső, JózsefQiao, YongliangDhakal, AshimQin, Cheng-ZhiDhakal, RajendraQin, DanyangDhakshinamoorthy, AmarajothiQin, DonghuanDhanjai, DhanjaiQin, FeiDhaou, Imed BenQin, HongdeDi Angelo, LucaQin, HongyuDi Barba, PaoloQin, Jiao-huaDi Blasio, GabrieleQin, LiguoDi Bona, GianpaoloQin, ShiqiaoDi Ciaccio, FabianaQin, YechenDi Domenico, SimoneQin, YiDi Federico, VittorioQin, ZhaoyeDi Flumeri, GianlucaQin, ZhenDi Fulvio, AngelaQin, ZongDi Gravio, GiulioQing, XinLingDi Leo, GiuseppeQingyuan, ZhuDi Lorenzo, EmilioQiu, ChenDi Marco, AlessandroQiu, GuangyuDi Marco, RobertoQiu, HaiDi Martino, FerdinandoQiu, JianbinDi Martire, DiegoQiu, Jing-HuiDi Masi, SabrinaQiu, QiwenDi Mauro, MarioQiu, SenDi Natali, ChristianQiu, TianDi Nunzio, LucaQiu, WeibaoDi Nuovo, AlessandroQiu, XianboDi Palma, PasqualeQiu, YongjianDi Pietro, PaolaQiu, YubaoDi Sieno, LauraQiu, ZhenDi, ZhenhuaQu, FengzhongDiaconita, VladQu, HaiboDiaconu, Daniel ConstantinQu, HongweiDiamantas, SotiriosQu, MuchaoDiamantoulakis, Panagiotis D.Qu, RuitaoDiamond, SolomonQu, XiaoleiDias Da Cunha, RudneiQu, YonghuaDias, Fernando SuparreguiQuan, GaofengDias, PauloQuan, WeiDíaz Redondo, Rebeca P.Quang Trung, TranDiaz, Francisco AlbertoQuattrini, RamonaDiaz, Jose ManuelQuattrocchi, AntoninoDiaz, MoisesQue, LongDíaz-Alonso, DanielaQueluz, Maria PaulaDíaz-Álvarez, AntonioQuer, StefanoDiaz-Guerra, DavidQuero, GiuseppeDíaz-Otero, Francisco J.Quero, José ManuelDiCarlofelice, AlessandroQuest, DaleDickert, Franz L.Quevedo, EduardoDickey, MichaelQuevedo, MiguelDickmann, FrankQuevedo-Gutiérrez, EduardoDiculescu, VictorQuinlan, JasonDiddens, DiddoQuinn, Mark KennethDieber, BernhardQuintana, Penelope.J.E.Diederich, ChrisQuintero M., Christian G.Diego, Belén CurtoQuoc Le, Trung (Tim)Diethe, TomQuochi, FrancescoDietz, Marina S.Qureshi, AmnaDiez Blanco, Luis EnriqueRabaev, IrinaDíez, Luis FranciscoRabatel, GillesDiez, YagoRabe, UteDíez-González, JavierRabelo, RicardoDíez-Jiménez, EfrénRabiee, RamtinDiFino, AngeloRabinovici, RaulDigulescu, AngelaRackauskas, SimasDillingham, PeterRackiene, RomaDillon, Laura W.Rackus, Darius G.Dilo, ArtaRácz, AnitaDilonardo, ElenaRadac, Mircea-BogdanDimakis, NicholasRadanliev, PetarDimcheva, NinaRadicioni, FabioDimitrakis, GeorgiosRadko, Sergey P.Dimitriou, NikolaosRadkowski, RafaelDimitropoulos, KosmasRadlak, KrystianDimitrov, BorislavRadoi, AnamariaDimou, AnastasiosRadonic, VasaDimoulas, CharalamposRadu, AleksandarDina, RăzvanRaducanu, Bogdan C.Dinardo, GiuseppeRadulescu, MirceaDing, FanRadvan, RoxanaDing, FengRadzieński, MaciejDing, GuoruRadziszewska-Zielina, ElżbietaDing, JiachenRadziuk, DaryaDing, JunjunRaetz, SamuelDing, KangRaffaeli, RobertoDing, ShihongRaffelsberger, ChristianDing, WenboRagia, LemoniaDing, YichunRagulskis, MinvydasDing, YongRagusa, EdoardoDing, YuanzhaoRahim, Md. Noor ADing, Yuemin (China)Rahimi Nohooji, HamedDing, Yuemin (Norway)Rahimi, AfshinDing, ZhenyangRahimi, FaridDing, ZhizhongRahimian, ArdavanDinh, AnhRahimpour, AlirezaDinh, ToanRahimzadeh, AminDinis, Maria De LurdesRahman, AkhlaqurDinis, RuiRahman, AshfaqurDinulovic, DraganRahman, HabibDionísio, RogérioRahman, MizanoorDios, FedericoRahman, Mohammad MahfujurDipnall, JoannaRahman, MoshiurDiraco, GiovanniRahman, MostaqurDissanayake, ManjulaRahman, MuhibDitroilo, MassimilianoRahman, SaydurDivić, VladimirRahmani, RahimDixon, Adam J.Rahmat, AminDixon, BrandonRahmati, MehdiDixon, SteveRahmatian, ArashDiykh, MohammedRai, LaxmishaDizo, JanRaida, ZbynekDjenouri, YoucefRainieri, CarloDjeraba, ChaabaneRaiyn, JamalDjerada, ZoubirRajahonka, MerviDjeziri, Mohand ArabRajamanickam, YuvarajDjokovic, RadojicaRajan, ParthibanDjordjevic, Goran Lj.Rajanen, DorinaDługosz, MarekRajaram, DushhyanthDługosz, RafałRajaram, ShankarDmitriev, AlexeiRajchowski, PiotrDo Nascimento, Marcelo ZanchettaRajesh, KrishnamurthyDo Rosário, Denis LimaRajewski, RemigiuszDo, Dinh-ThuanRajšp, AlenDo, Quang HungRajtukova, ViktoriaDo, Thanh NhoRak, JanuszDo, TronghopRakoczy, AnnaDobešová, ZdenaRama, Estefanía CostaDoblas, AnaRamacher, SebastianDobre, CiprianRamadan, FawzyDobre, Robert-AlexandruRamadi, KhalilDobrea, Dan-MariusRamadoss, AnanthakumarDobrescu, LidiaRamalingam, BalakrishnanDobrev, IvoRamanaviciene, AlmiraDobrev, YassenRamanavicius, ArunasDobreva, AntoanetaRamaraj, SukanyaDobrzanska, MagdalenaRamasso, EmmanuelDobrzycki, ArkadiuszRambach, JasonDocoslis, ArisRambault, LaurentDodd, Emma M. A.Ramella, GiulianaDodi, GianinaRamesh, BharathDodi, GiuseppeRamirez Lopez, Leonardo JuanDoeven, EganRamirez Miquet, EvelioDogan, SafakRamirez, AlejandroDogandzic, AleksandarRamírez, Joan ManelDogaru, RaduRamirez, Juan ManuelDoh, Deog-HeeRamírez-Faz, JoseDokladal, PetrRamirez-Gonzalez, GustavoDolci, MathiasRamirez-Mendoza, Ricardo AmbrocioDołęga, WaldemarRamos González, VictoriaDolenec, RokRamos, Ana Paula MarquesDoley, DavidRamos, DanielDolez, PatriciaRamos, José A.Dolezel, PetrRamos, VictorDolganov, Pavel VladimirovichRamos-Jiménez, ArnulfoDolgikh, Grigory IvanovichRampa, VittorioDoll, KonradRamser, KerstinDolnik, BystrikRan, RongDomaneschi, MarcoRana, Abu Ul Hassan SarwarDomanska, JoannaRanagalage, ManjulaDomański, AdamRanc, VáclavDombek, GrzegorzRandazzo, AndreaDomenjoud, MathieuRandazzo, VincenzoDomin, JaroslawRaney, KeithDomingues, InêsRangel De Sousa, FernandoDomingues, Maria FátimaRangel, PabloDomínguez Brito, Antonio CarlosRangelow, IvoDominguez, DavidRanjan, AlokDomínguez, Jorge LuisRanjan, RishabhDominguez, ManuelRanjbarzefreh, MahdiDomínguez, RaulRanjit, SumanDominguez, Rocio B.Ranky, PaulDominguez, SergioRantos, KonstantinosDominguez-Bolano, TomasRao, Apparao M.Domínguez-Morales, Juan P.Rao, JianDomínguez-Morales, Manuel JesúsRao, LangDominguez-Pumar, ManuelRao, MuzaffarDomnikov, PetrRao, SmithaDonadello, SimoneRaparelli, TerenzianoDonaire-Avila, JesúsRapin, MichaelDonaldson, NickRaposo, MariaDonat, FelixRascon, CalebDonati, MassimilianoRashedi, EhsanDonati, SilvanoRasheed, TahirDondajewska-Pielka, RenataRashid, UsmanDonelli, MassimoRashid, ZulqarnainDong, BoweiRasmussen, MichelleDong, Chuan-ZhiRaspopoulos, MariosDong, DianbiaoRasskazov, IliaDong, GanggangRassõlkin, AntonDong, JunliangRasti, PejmanDong, LongjunRatassepp, MadisDong, MingliRathod, VivekDong, NaRathod, Vivek T.Dong, QinglinRathore, HeenaDong, QiuchenRatiu, AndreeaDong, ShaochunRato, Ana ElisaDong, ShurongRauch, ŁukaszDong, Suh-YeonRauch, MatthieuDong, WeihuaRaumonen, PasiDong, WenRauti, SampsaDong, XiaolongRavan, MaryamDong, XichaoRavankar, AbhijeetDong, XiuzhenRavankar, AnkitDong, YingRavelo, BlaiseDong, YunquanRavina, MarcoDong, ZaopengRavindra, NuggehalliDong, ZiqianRavindran, PrabuDonlagic, DenisRawassizadeh, RezaDonner, ReikRay, BiplobDonzella, ValentinaRay, ParthaDorafshan, SattarRay, SujayDordevic, DaniRay, SupriyoDorizzi, BernadetteRaymundo-Pereira, Paulo A.Dorneles, Lucio StrazzaboscoRaza, UsmanDoronzo, Domenico M.Razenkov, IgorDoroz, RafalRazvan, MohammadrezaDorri, AliRazvarz, SinaDos Santos Prol, FabricioRe, CristinaDos Santos, Eduardo NunesReali, GianlucaDos Santos, José Cleiton SousaRealpe, MiguelDos Santos, Paulo Cezar RochaRébillat, MarcDoshi, NeelRebordão, José ManuelDossi, CarloRecchia, Daniela R.Dou, WenRecchiuto, CarmineDoulamis, AnastasiosRedding, BrandonDoulamis, NikolaosReddy, Yenumula B.Doumenis, GregoryRedkar, SangramDoussan, ClaudeRedweik, PaulaDowrick, ThomasRees, GarethDoyle, DanielReeve, KathleenDraganov, IvoRegazzoni, DanieleDragic, PeterReggiani, LucaDrago, CarloReggiannini, MarcoDragomir, AlinRegmi, BishnuDragomir-Stanciu, DanielRego, GasparDragonieri, SilvanoRego, PaulaDragulinescu, AndreiRegueiro, Carlos V.Drajic, DejanRegueiro-Ferreira, Rosa MaríaDrap, PierreRehm, EricDrapaca, CorinaRehman, SaeedDrapikowski, PawelReich, ChristianDresp-Langley, BirgittaReich, ChristophDressel, MartinReiche, Christopher F.Drewniak, JózefReid, Russell C.Drewniak, SabinaReiff-Marganiec, StephanDrexler, DanielReig, CàndidDrgona, PeterReig, GemmaDrioli, CarloReina, Daniel GutiérrezDrissi-Habti, MonssefReinaldo Ribeiro, FernandoDrobics, MarioReina-Romo, EstherDrobiec, ŁukaszReindl, LeonhardDrossos, KonstantinosReinoso, OscarDroz, ChristopheReis, ArsénioDrozd, JanReis, JoelDrozdowski, PawelReis, Manuel J.C.S.Drozdziel, PawelReiser, DavidDrummond, ColinReiser, RaoulDrusa, MarianReising, Donald R.Du, GuanglongReiss, Lucinda V.Du, HaishunReithmeier, EduardDu, HuaqiangRejah, ShahiDu, Jiang BingRejer, Hab. IzabelaDu, JianhaoRejfek, LubosDu, JinglinRekas, MieczyslawDu, JinyangRembe, ChristianDu, Li DongRen, Hai PengDu, LipingRen, HaoshanDu, QifeiRen, JianfengDu, ShoujiRen, JiankangDu, ShuangRen, LiangDu, SijunRen, LiminDu, WinncyRen, MingDu, XinRen, QinlongDu, XinliRen, ShangjieDu, YegangRen, ShengbingDu, ZhenhuiRen, Shun-qingDuan, BenchunRen, Tsang IngDuan, FengRen, WeiDuan, Huan-FengRen, WuweiDuan, LingzeRen, YukunDuan, QiangRen, YuzhuoDuan, WeiliRen, ZhileDuan, XuechaoRen, ZhiyuanDuan, YongRenard, Jean-BaptisteDuan, YuanfengRenaudin, ValérieDuarte Correia, SérgioRende, Sante FrancescoDuarte, MarcosRenkwitz, ToralfDubey, HarishchandraRenner, Bernd-ChristianDubey, RameshwarRennhofer, HaraldDubuc, BrennanRenza, DiegoDudczyk, JanuszRenzaglia, AlessandroDudek, MichalRepanovici, AngelaDudescu, Mircea CristianRepelianto, Ageng S.Duenas, Jose A.Rerucha, SimonDuffo, NuriaReshetnyak, VictorDugelay, SamanthaResque Dos Santos, Carlos GustavoDugonjić Jovančević, SanjaRetiere, FabriceDuka, Adrian-VasileRetscher, GuentherDulebenets, Maxim A.Retscher, GüntherDulf, Eva H.Retterer, John MichaelDulińska, Joanna MariaRettig, RasmusDuma, Virgil-FlorinReuland, YvesDumbrava, VirgilRevel, Gian MarcoDumciene, AudroneRevelou, Panagiota-KyriakiDumić, EmilRevollar, SilvanaDumitrascu, DanutRexha, BlerimDumitrescu, AnamariaRey, GaëtanDumitrescu, MarianaReyes Vera, ErickDumstorff, GerritReyes, Bersain A.Dumur, FrédéricReyes-González, ArturoDunai, LarisaReyna Maldonado, AlbertoDunaj, PawełRezaee, MohammadDung, Le TheRezaei, MahdiDung, Nguyen DinhRezai, PouyaDunham, SimonRezaie, RezaDunn, BruceRezapour Mashhadi, Mohammad MahdiDunstan, JocelynRezig, SadokDuong, Chi NhanŘezník, TomášDuplaga, MariuszRezvani, MohsenDuplančić Leder, TeaRezzoug, NasserDupré, AntoineRhee, Jong IlDupuis, KateRheinwalt, AljoschaDupuis, RomainRi, ShienDupuy, LionelRianna, GuidoDuque, OscarRiaz, QaiserDuque, RafaelRiazul Islam, S.M.Duque-Domingo, JaimeRiba, Jordi-RogerDuraibabu, Dinesh BabuRibatski, GherhardtDuran, CristhianRibeiro Costa, Nuno AlexandreDurana, PavolRibeiro Pinto, JoãoDurdán, MilanRibeiro, Bruno TeixeiraDurdaut, PhillipRibeiro, Eduardo ParenteĐurić, Bogdan OkrešaRibeiro, Sergio SilvaDuric, NebRibes-Pleguezuelo, PolDurini, DanielRibichini, RemoĎuriš, StanislavRicaud, BenjaminDuroc, YvanRiccardi, UmbertoDutoit, BertrandRicchi, AntonioDutta, ArindamRicci, LorenzoDutta, AyanRicci, MarcoDutta, GaurabRicci, StefanoDutta, ParikshitRicciardella, FilibertoDutta, SandipRicciardi, ArmandoDuvigneau, FabianRicciardi, GuillaumeDuygu, SarikayaRiccio, DanielDwivedi, Ashutosh DharRiccioli, CeciliaDworak, VolkerRicciuti, ManolaDymora, PawełRicco, RaffaeleDyo, VladimirRichards, ToddDyrcz, CzesławRichardson, Andrew G.Dyson, MatthewRichardson, JustinDzantiev, BorisRichelli, AnnaDzantiev, Boris B.Richtera, LukášDzedzickis, AndriusRico Yuste, AlbertoDzidic, AlenRico, Ulises PinedaDziong, ZbigniewRico-Yuste, AlbertoDziuda, ŁukaszRida, ImadDziurdzia, PiotrRidolfi, MatteoDziurzanski, PiotrRiebe, DanielDzwonkowski, ArielRiel, AndreasE., YiwenRifà-Pous, HelenaEady, MatthewRiggio, MariapaolaEagle, Shawn R.Righi, MarcoEbel, ThomasRighi, RodrigoEbong, Abasifreke U.Riguidel, MichelEbrahimi Atani, RezaRiid, AndriEbrahimi Salari, MahdiRijckaert, HannesEbrahimi, AidaRim, MinjoongEbrahimi, AmirRimal, BhagawatEbrahimkhanlou, ArvinRimár, MiroslavEbralidze, Iraklii I.Rinaldi, StefanoEcheverria, ElenaRincón, ElviraEchigo, TomioRinkenauer, GerhardEckelkamp, ElizabethRinkevich, AnatolyEdahiro, MasatoRio-Belver, RosaEddingsaas, NathanRiordan, DanielEdelman, Bradley (Germany)Rios-Aguilar, SergioEdelman, Bradley (USA)Rios-Figueroa, Homero VladimirEdgington, DuaneRios-Navarro, AntonioEdler, DennisRioual, ‪StephaneEdmondo, BattistaRipka, PavelEdo, Maria BermudezRiquelme, FabiánEdomwonyi-Otu, Lawrence C.Rissolo, DominiqueEdwan, EzzaldeenRistic, MichaelEdwards, EverardRistic-Durrant, DanijelaEdwards, HelenRistorini, MartinaEdwards, KeithRistović, IvicaEersels, KasperRita, Fuentes-AguilarEfremova, Elena V.Ritrovato, PierluigiEfremova, NataliaRitter, TimEftimov, TinkoRiul Junior, AntonioEgarievwe, Stephen U.Riurean, SimonaEggebrecht, Adam T.Rivas-Blanco, IreneEggers, TatianaRivera, DiegoEgli, RamonRivera, GilbertoEgorov, EgorRivera, JorgeEguía, PabloRivera, Zandra BetzabeEguíluz, Augusto GomezRivero-Angeles, Mario EduardoEguiraun, HarkaitzRivertz, Hans JakobEhrlich, KatjanaRizal, ConradEhrlich, Stefan K.Riziotis, ChristosEibl, EvaRizvi, Syed Tahir HussainEisemann, MartinRizzardi, AlessandraEisencraft, MarcioRizzi, Gian AndreaEitzinger, ChristianRizzi, MariaEiza, Max HashemRizzolo, SerenaEjaz, WaleedRmayti, MohammadEkinci, Yunus LeventRo, Jong-SukEkmekcioglu, ErhanRoa Rovira, Joan JosepEkonomou, LambrosRoatǎ, Ionuţ ClaudiuEkpo, SundayRoatta, SilvestroEkstrom, MikaelRobert, TenzerEkwaro-Osire, StephenRoberto, FazioEl Kharraz, JauadRoberts, CraigElagin, VasiliyRobinson, NicoleElahi, HassanRobitzsch, AlexanderElango, JeevithanRobles, GuillermoElangovan, VinayakRobles, Ramiro SamanoElbahhar, FouziaRobustelli, UmbertoElberink, Sander OudeRoccaro, PaoloElble, Rodger J.Rocchetti, GabrieleElduque, DanielRocchi, IreneElena, HusanuRoccotelli, MicheleEleni, MakaronaRocha Vaz, JoãoEler, Danilo MedeirosRocha, AtslandsElfergani, IssaRocha, JorgeElfouly, TarekRocha, PauloElgendy, MostafaRoche, FrédéricElghamrawy, HaidyRodehorst, VolkerEl-Ghazaly, AmalRodella, IlariaElhattab, AhmedRodrigues, AntonioElia, StefanoRodrigues, EduardoEliades, MarinosRodrigues, FernandaEliáš, PetrRodrigues, Luis MonteiroElias, SaidRodrigues, MarcosEliopoulos, Panagiotis A.Rodrigues, Mário S.Elkatatny, SalaheldinRodrigues, Thiago BragaElken, JüriRodrigues, VarleiElkin, ColinRodriguez Arreola, AlbertoEllul, RaymondRodriguez Diaz, Camilo ArturoElmashhara, Maher GeorgesRodríguez Hermosa, Juan LuisElmazria, OmarRodríguez Pérez, MiguelElRashedy, RemahRodriguez, AlejandroEl-Schich, ZahraRodriguez, AlvaroElshaer, AhmedRodriguez, AngelEl-Shafee, Omar OsamaRodriguez, Apolinar MuñozElshaw, MarkRodriguez, Aurelio HierroElsobky, MouradRodriguez, CelestinoElsts, AtisRodriguez, DionisioElvira, NicaRodríguez, Francisco J.Ely, FernandoRodríguez, José-VíctorElyan, EyadRodriguez, Juan ManuelEmadi, ArezooRodriguez, JuvenalEmaldi, MikelRodriguez, NibaldoEmberton, SimonRodriguez, NoelEmery, JohnRodríguez, Orlando CamargoEmilio, SardiniRodriguez, SergioEmri, MiklosRodríguez-Archilla, AlbertoEncarnação, PedroRodríguez-Delgado, Melissa M.Endisch, ChristianRodriguez-Esteban, RaulEndo, HiroyukiRodríguez-Gonzálvez, PabloEndo, Patricia TakakoRodríguez-Hernández, María Del CarmenEndo, TatsuroRodríguez-Lara, Blas ManuelEndriulaitienė, AuksėRodríguez-Licea, Martín-A.Engeberg, Erik D.Rodriguez-Lorenzo, LauraEngel, JonathanRodriguez-Lozano, Francisco J.Engelbrecht, RainerRodriguez-Martin, ManuelEngelhardt, Paul E.Rodríguez-Martínez, AlbertoEngler, EvelinRodriguez-Mendez, Maria LuzEnguita, José MaríaRodriguez-Navas, GuillermoEnright, JohnRodriguez-Pino, MarcosEnser, HerbertRodriguez-Pulido, Francisco J.Entrena Arrontes, Luis AlfonsoRodriguez-Resendiz, JuvenalEo, YangDamRodríguez-Rodríguez, IgnacioEom, Il KyuRodriguez-Sanchez, CristinaEon, RehmanRodriguez-Zurrunero, RobertoEpelbaum, StephaneRodruiguez, Luiz Carlos EstravizEpelde, GorkaRoehrich, JozefEpiphaniou, GregoryRoffia, LucaEramo, VincenzoRogerio Pinheiro, PlácidoErden, FatihRogers, ChristopherErhart, JiřiRogers, EricErivan, RogerRoggen, DanielErlandsson, KjellRogulj, KatarinaErmolov, IvanRoh, Byeong-heeErrapotu, Sai MounikaRoh, ChanghyunErshov, AlexeyRoh, Jong WookErturk, EmreRoh, YongraeEscobar-Gómez, ElíasRohac, JanEscobedo, CarlosRohacs, JozsefEscobedo, PabloRohan, Pierre-YvesEscola, José Javier AstrainRöhrig, ChristofEscorihuela, JorgeRoig, Ignacio BoschEscudero, Carlos J.Rojas Sola, JoséEscudero, OlgaRojas, Christian A.Esen, CemalRojek, IzabelaEsfahani Monfared, YasharRojhani, NedaEsfahani, AlirezaRojo Baio, Fábio HenriqueEsfahani, SiavashRojo-Álvarez, José LuisEsfandiarpour, FatemeRokicki, TomaszEskelinen, Matti A.Rokosz, KrzysztofEsmaeeli, RojaRoldán-Blay, CarlosEsmerian, KarekinRolfe, PeterEspejo-García, BorjaRomagnoni, PiercarloEspinilla, MacarenaRomain, RossiEspinosa, Hugo G.Roman, AnamariaEspinosa, JuliánRoman, Raul CristianEspinoza, Carlos ZúñigaRoman, SophieEsposito, AntonioRomán-Castro, RodrigoEsposito, FlavioRomanchik Kriuchkova, EugenioEsposito, GianlucaRomanets, YuriyEsquivel, KarenRomaniuk, MaciejEstatico, ClaudioRomano, DonatoEsteban, JaimeRomano, GianmarcoEstelrich, JoanRomanovskii, Oleg A.Estepa, RafaelRomanowicz, Paweł J.Estêvão, João M. C.Romeira, BrunoEsteves Da Silva, Joaquim C.G.Romeo, KaterineEstrada-López, Johan J.Romeo, LucaEstrada-Moreno, AlejandroRomeo, StefaniaEstrela, Vania V.Romero Morales, CarlosEstudillo-Ayala, Julian M.Romero, Fernando CastanoEtoz, SevdeRomero-Diaz, PabloEudes, AlexandreRomero-Sanchez, MartinEugene, YakovlevRomero-Sanchez, Martin EnriqueEugenio, FazioRomine, WilliamEvan, PengRomo Pérez, VicenteEvangelides, ChristosRon-Angevin, RicardoEverett, MarkRoncella, RiccardoEvsutin, OlegRondeau, EricEvtugyn, GennadyRonen, ShukiEwusi-Annan, EboRong, GuoxinExarchos, Dimitrios A.Rong, YuEyer, KlausRonita, BardhanEyobu, Odongo StevenRönnholm, PetriEzquer, IgnacioRonzani, DanieleFabbiano, LauraRoohi, MiladFabian, PiotrRoose, PhilippeFabianowski, WojciechRopelewska, EwaFabris, MassimoRoper, CourtneyFacca, ChiaraRopero, JorgeFadda, MauroRoques, ChristianFadden, ChrisRoriz, PauloFadlullah, ZubairRos, FredericFadul-Pacheco, LilianaRosa, Bruno M.G.Fafoutis, XenofonRosário, DenisFagadar-Cosma, EugeniaRosati Papini, Gastone PietroFaghri, ArdeshirRosati, GiulioFagiolini, AdrianoRoscow, JamesFahn, Chin-ShyurngRose, DestanieFaifer, MarcoRoseiro, LuísFaisal Haider, MohammadRoselli, IvanFajkus, MarcelRosenberg, LukeFakhari, AminRosenberger, Albert T.Fakhfouri, ArmaghanRosenberger, ChristopheFakhr Hosseini, ShabnamRosenfeld, AnatolyFalade, Oluyemi PeterRosenholtz, RuthFalaschetti, LauraRosenkranz, AndreasFalchi, FabrizioRosina, ElisabettaFalco, AntonioRosique Contreras, María FranciscaFalco, ClaudioRosner, DanielFalco, GregoryRosolem, Joao BatistaFalk, RainerRossi, AlessioFalkowski-Gilski, PrzemyslawRossi, ClaudioFallucchi, FrancescaRossi, Giovanni BattistaFamouri, ParvizRossi, LorenzoFan, Deng-PingRossi, MassimilianoFan, HongRossi, René M.Fan, HongqiRossi, StefanoFan, HuaRossignol, JérômeFan, JunchuanRossner, FlorianFan, KangqiRosso, CarloFan, Kuang-ChaoRosu, Stefan GeorgeFan, LeiRotariu, CristianFan, LiyangRothacker, LeonardFan, MengbaoRothbauer, MarioFan, MiaoRothhardt, ManfredFan, OuyangRothkrantz, LeonFan, PingyiRoths, JohannesFan, Shu-Kai S.Roubertoux, PierreFan, XingwangRoussos, EvangelosFan, XugeRoux, StéphaneFan, YinRoveda, LorisFan, YuanchengRovida, FrancescoFan, Yu-ChengRovini, ErikaFan, ZhaoyanRoy, AbhishekFancy, Saiada FuadiRoy, DhruvajyotiFang, FeiRoy, SayanFang, JianRoy, SomeshwarFang, JianwuRoy, SpandanFang, LimingRoy, SudiptaFang, MingRoy, Tushar KantiFang, PengRoyer, Jean-JacquesFang, RongxinRóżański, AdrianFang, Shih-HauRozsival, PavelFang, YiRuan, CunjunFang, ZaojunRuan, JunhuFanghella, PietroRuan, XiaojunFanjul-Bolado, PabloRubega, MariaFanjul-Vélez, FélixRubenstein, Dustin R.Fanti, AlessandroRubini, SanthaFantoni, AlessandroRubio Alonso, HiginioFapojuwo, AbrahamRubio Loyola, JavierFarago, PaulRubio, Jose De JesusFarahani, Behzad V.Ruby, RukhsanaFarajikhah, SyamakRucci, AlessioFaralli, MicheleRucco, MatteoFaria, Brígida MónicaRucco, RosariaFarid, ArvinRucka, MagdalenaFarid, FarnazRudnicki, KonradFarid, Muhammad ShahidRudnitskaya, AlisaFarina, LauraRüdt, MatthiasFarka, ZdeněkRueckert, ElmarFarlik, JanRuelas, AdolfoFarmer, MichaelRuescas, Ana BelenFarnesi, DanieleRuffert, ChristineFarooq, MuhammadRuffin, PaulFarrokhifar, MeisamRufino, GiancarloFarrow, Reginald C.Ruggeri, PaoloFarrugia, LourdesRuggieri, SergioFarshbaf, SepidehRughoobur, GirishFarup, IvarRugini, LucaFarzampour, AlirezaRuiperez Valiente, Jose AntonioFasano, AndreaRuiz Armenteros, Antonio MiguelFasbender, DominiqueRuiz Martínez, AntonioFascista, AlessioRuiz, Jordi-Roger RibaFaseeh Qureshi, Nawab MuhammadRuiz, MercedesFasel, BenediktRuiz, Vicente GonzálezFassi, FrancescoRuiz-Díez, VíctorFathi, SoheilRuiz-Jimenez, JoseFathy, YasminRuiz-Munoz, Jose FranciscoFattahi, JaouharRuiz-Sarmiento, Jose RaulFattoruso, GraziaRuiz-Valdepeñas Montiel, VíctorFattouh, AnasRullo, AntoninoFaustino, AnaRulseh, Aaron MichaelFauzi, IlianaRumyantseva, MarinaFaye, SébastienRundo, FrancescoFazzolari, RoccoRuo Roch, MassimoFeatherstone, WillRuotolo, FrancescoFebbraio, FerdinandoRuotsalainen, LauraFedele, RosarioRuppert, Michael G.Fedeli, AlessandroRus, Lecturer LiviuFederic, MichardRus-Casas, CatalinaFediuk, RomanRusinek, RobertFedorko, GabrielRusnák, MilošFedorov, Fedor S.Russ, JohnFedorov, GeorgyRusso, MariateresaFeeney, AndrewRusso, Mario VincenzoFei, TaiRusso, MatteoFei, YiyanRusso, MicheleFeis, AlessandroRusso, MladenFeito Higueruela, Francisco R.Rusu, CristinaFekete, ZoltánRutagemwa, HumphreyFekr, Atena RoshanRutkauskas, SauliusFeld, SebastianRutkowska, JaroslawaFeldt, MarkusRutkowski, LucileFeliciani, ClaudioRuz, Jose JaimeFelici-Castell, SantiagoRužarovský, RomanFelis Enguix, IvanRuzgas, TautgirdasFelis, IvanRuzgys, PauliusFelisberto, PauloRužić, IgorFeliu, VicenteRyadnov, MaxFelix, Anderson AndréRybak, JarosławFemine, Antonio DelleRybnikova, Nataliya A.Femminella, MauroRybus, TomaszFendrych, MatyasRydosz, ArturFeng, BotaoRykov, YuriFeng, ChuangRyl, JacekFeng, FangchenRymarczyk, TomaszFeng, Guang-LiangRyndin, Eugeny A.Feng, GuojinRyndzionek, RolandFeng, Hsuan-MingRyoo, Young-JaeFeng, KunpengRys, DawidFeng, LianRysak, AndrzejFeng, LiangbingRyu, ChanseokFeng, PhilipRyu, HyejeongFeng, QiboRyu, Jae-MyungFeng, RuyiRyuh, YoungsunFeng, ShengRyzhii, Maxim V.Feng, ShijieRząsa, MariuszFeng, ShilunRzepecka, ZofiaFeng, ShuanglongRzhanov, YuriFeng, YanmingRzucidlo, PawelFeng, YaokaiSaadane, RachidFeng, YaozeSabatini, RobertoFeng, YunfeiSabban, AlbertFeng, ZhouSabchevski, Svilen PetrovFerenc, RieszSabella, DarioFerguson, Frank T.Saber, MohamedFermino, Rogério CésarSaberioon, MohammadmehdiFernandes, Ana Patrícia Da SilvaSabharwal, AshutoshFernandes, FábioSabsabi, MohamadFernandes, HenriqueSacala, IoanFernandes, MarceloSǎcǎleanu, Dragoş IoanFernandes, TelmoSaccomandi, PaolaFernández Gavela, AdriánSachau, DelfFernandez Grande, EfrenSachs, JürgenFernández Iglesias, DiegoSadeghi Esfahlani, ShabnamFernández Jaramillo, Arturo AlfonsoSadeghi, SheilaFernandez Olvera, Anuar De JesusSadiq, Ali SafaaFernández Ramos, María DoloresSadjadi, FiroozFernandez, AlbertoSadjadi, Firooz A.Fernández, AntonioSadok, IlonaFernández, María GarcíaSadowski, ŁukaszFernandez, RoemiSadre, RaminFernández-Ahumada, Luis ManuelSaeed, KhalidFérnandez-Batanero, Jose M.Saeed, NaghamFernández-Caramés, Tiago M.Saeed, NasirFernandez-Garcia, RaulSaenz-Cogollo, Jose FranciscoFernandez-Granero, Miguel AngelSafaei, MohsenFernández-Isabel, AlbertoSafanelli, José LucasFernandez-Lopez, AntonioSafar, LeosFernández-Pacheco, Daniel G.Šafarič, RikoFernández-Pousa, Carlos R.Saffache, PascalFernandez-Prieto, ArmandoSaffell, JohnFernandez-Roldan, Jose AngelSafonov, Alexander AleksandrovichFernández-Ruiz, María R.Safonov, IliaFernández-Sánchez, CésarSaggin, BortolinoFernandez-Veiga, ManuelSagiya, TakashiFernandez-Vidal, Severo RaulSaha, Jhantu KumarFernández-Yáñez, PabloSaha, Rony KumerFernando, XavierSaha, SangeetFerraioli, LuigiSaha, SudipanFerrández Vicente, José ManuelSahin, CanerFerrández-Pastor, Francisco JavierSaho, KenshiFerrandiz, Jose ManuelSahoo, SubratFerrando-Villalba, PabloSahore, VishalFerrante, AlbertoSahour, HosseinFerrara, Maria AntoniettaSaia, RobertoFerrari, PaoloSaifuzzaman, MdFerrari, RosalbaSaijo, YoshifumiFerrari, SimoneSaiko, GuennadiFerrario, Maria FrancescaSaikumar, NiranjanFerraris, ClaudiaSailer, RudolfFerraro, AngeloSaini, IkjotFerraro, VenereSaini, RajkumarFerrazani Mattos, Diogo MenezesSainz De Abajo, BeatrizFerré-Borrull, JosepSaita, YusukeFerreira Lousado, José PauloSajavičius, SvajūnasFerreira, Aida AraújoSajjanhar, AtulFerreira, AnaSakač, NikolaFerreira, André G.Sakagami, NorimitsuFerreira, ArmandoSakamoto, João Marcos SalviFerreira, ArturSakata, ShuzoFerreira, Daniel BassoSakellariou, MichaelFerreira, FaustoSakhabutdinov, AiratFerreira, FernandoSakhabutdinov, Airat ZhFerreira, HugoSakic, PierreFerreira, JoaoSakkalis, VangelisFerreira, Joao C.Saknīte, IngaFerreira, JoaquimSakon, TakuoFerreira, Karine ReisSakurai, KouichiFerreira, LucasSałabun, WojciechFerreira, LuisSałaciński, TedeuszFerreira, Luís PintoSalafia, Marco GiuseppeFerreira, Nuno M. F.Salah, KhaledFerreira, Paulo Victor R.Salahdine, FatimaFerreira, RicardoSalam, AdulFerreiro-González, MartaSalami, Mohammad RezaFerrer, GonzaloSalamone, FrancescoFerrer, Miguel A.Salata, StefanoFerrera, MaximeSalauddin, MdFerrero Martín, Francisco J.Salazar, AddissonFerrero, AndreaSalazar, SergioFerrero, FabienSalceda-Delgado, GuillermoFerri, GiuseppeSalcedo, Walter J.Ferrigno, GiancarloSaleme, Estêvão BissoliFerro, AlfredoSalice, FabioFerro-Famil, LaurentSalido Tercero, JesúsFerruz, JoaquinSaliga, JanFesta, Demetrio CarmineSaligane, MehdiFetaya, EthanSalimi, MehdiFetisov, LeonidSalinas, CarlotaFetisov, YuriSalinet, JoaoFhager, Lars OhlssonSalisbury, JosephFiala, PavelSalkuti, Surender ReddyFialho, VitorSalmane, HoussamFiani, MargheritaSalminen, KatriFicco, MassimoSalnitri, MattiaFice, MartynSalpavaara, TimoFicko, MirkoSaltaren, RoqueFidalgo, Jesús LópezSaltas, VassiliosFidani, CristianoSalvador, GilFiedler, GoeranSalvati, DanieleFiedler, MarkusSalvi, MassimoField, MatthewSalvo Rossi, PierluigiFiglus, TomaszSalvo, PietroFigueira, FlavioSama, MichaelFigueira, Rita BacelarSama, Natalia FalaganFigueiras, EditeSaman, ParvanehFigueiredo, LinoSamanta, Pralok KumarFigueiredo, Nuno M.Samaras, IoannisFigueras, EduardSamat, AlimFigurski, MariuszSamborski, SylwesterFigwer, JarosławSametinger, JohannesFilchev, LachezarSamiappan, SathishkumarFilipe, José AntonioSamiei, EhsanFilippeschi, AlessandroSammut, CharlesFilippov, AndreySamouylov, Konstantin E.Filko, DamirSamoylov, Alexander M.Filo, GrzegorzSampaio, PaulaFingas, MervSampaolo, AngeloFink, GeoffSampath, KaushikFinkel, PeterSampietro, DanieleFinšgar, MatjažSamsonov, Timofey E.Fiore, UgoSanches De Andrade, Carlos EduardoFiorillo, AntoninoSanchez Aparicio, Luis JavierFiorillo, FaustaSánchez Egea, Antonio J.Fiorillo, FaustoSánchez Sutil, Francisco JoséFiorucci, MarcoSánchez, Antonio BernardoFischer, AndreasSánchez, BordelFischer, GeorgSánchez, Borja BordelFischer, IngaSánchez, Jairo R.Fischer, JanSánchez, Julio IsidroFischer, JeoffreySánchez, René-VinicioFischer, StefanSanchez, VictorFiscon, GiuliaSanchez-Cambronero, SantosFisher, Jeffrey S.Sánchez-Durán, Jose A.Fister, DušanSanchez-Gomez, JesusFitta, MagdalenaSanchez-Gordon, SandraFizzah-Jilani, SyedaSanchez-Lopez, Jose LuisFlammini, FrancescoSanchez-Lopez, Juan De DiosFletcher, KennethSánchez-Molina, Jorge AntonioFlorea (Voicu), CarmenSanchez-Montero, RocioFlorea, AdinaSanchez-Perez, GabrielFlorea, Bogdan CristianSánchez-Rodríguez, DavidFlorea, CorneliuSancho Bru, Joaquín LuisFlorea, LauraSander De Carvalho, Lino AugustoFlores, GerardoSanders, ChrisFlores-Fuentes, WendySandi, LjubicFloriano, Andrés GarcíaSandler, Hilary A.Floros, GeorgeSandner, ThiloFlynn, DavidSandoval Goes, Luiz CarlosFoit, KrzysztofSandoval-Herazo, Luis CarlosFok, Mable P.Sandra, RogerFokin, GrigoriySands, TimothyFokine, MichaelSandu, TitusFolgado, HugoSandwall, PeterFolke, MiaSang Yeob, KimFonod, RobertSang, MeiFonseca, AnaSang, ShengboFonseca, Elza Maria MoraisSang, Tzu-HsienFonseca, Manuel J.Sangili, ArumugamFonseca, PauSanislav, TeodoraFontanari, VigilioSanli, FusunFontes, RamonSannino, FilomenaFontgalland, GlaucoSano, Edson EyjiForcellini, DavideSanphuang, VaritthaForenbacher, IvanSan-Segundo, RubenForestiero, AgostinoSan-Segundo, RubénForke, RomanSant’Ana, JeanForlani, GianfrancoSanta, FernandoForliano, CanioSanta, JoséFornaro, ClaudioSantamaria-del-Angel, EduardoForner-Cordero, ArturoSantamaría-Peña, JacintoFornusek, CheSantana, JoseForouzanfar, MohamadSantarsiero, GiuseppeForrer, DanielSanthanagopalan, SunandForrester, StephSanti, EmanueleFörster, AnnaSanti, FabrizioFort, AdaSantiago-Rodríguez, EfraínForte, DomenicSantin, Altair OlivoForti, StefanoSantini, AntonelloFortuna, LuigiSantini, SilviaFosalau, CristianSantis, Adelmo DeFoster, Kenneth R.Santonastaso, Giovanni F.Foster, ScottSantonico, MarcoFoszcz, DariuszSantorelli, AdamFoti, CalogeroSantoro, SimoneFotia, LidiaSantos, AbelFotiadis, DimitriosSantos, AdrianoFotiou, NikosSantos, Auteliano A.Fotouhi, HosseinSantos, CristinaFoucher, Pierre-YvesSantos, Daniel Rodrigues DosFoulds, IanSantos, Eva M.Foumani, MehdiSantos, Filipe Neves DosFountas, SpyrosSantos, InêsFountoulakis, IliasSantos, IriaFourati, NajlaSantos, Jesús DanielFournier, GeorgesSantos, JoséFowler, AlisonSantos, Juan M.Főző, LadislavSantos, LuisFraddosio, AguinaldoSantos, MatildeFrade, MiguelSantos, Max Mauro DiasFradet, LaetitiaSantos, PauloFraga, Mariana AmorimSantos, RodrigoFragoso, AlexSantos, RubimFraile, Juan-CarlosSantos, TeresaFrämling, KarySantos-García, GustavoFrancais, OlivierSantosh, K.C.Francesca Carfora, MariaSantucci, ValentinoFrancesco, FeliciSantulli, CarloFrancés-Villora, José VicenteSantuz, AlessandroFrancik, SławomirSañudo, RobertoFrancini, AndreaSanwar Hosen, A.S.M.Francis, DanielSanz Ortiz, Ángel SantiagoFranciscangelis, CarolinaSanz Urquijo, BorjaFrancisco Algorri Genaro, JoséSanzo, AntonioFranco Silva, EdelbertoSapena-Baño, AngelFranco, AlessandroSaproo, SameerFranco, LeonardoSaquet, AdrianoFranco, WalfreSara, PensieriFrangi, AttilioSara, RadimFrank, RegineSarac, MineFranklin, Daniel R.Saraiva, Erlandson FerreiraFranko, AttilaSarangapani, JagannathanFränti, PasiSarbu, AndreiFrattini, DomenicoSarcevic, PeterFratu, OctavianSarda, EdoardoFraunholz, DanielSardesai, NaimishFredeunburg, ZacSardi, Giovanni MariaFredianelli, LucaSardianos, ChristosFreeborn, ToddSardone, RodolfoFreire, André PimentaSareen, HarpreetFreitag, FelixŠarić, MatkoFreitas Júnior, Walter Da CruzSarigiannidis, PanagiotisFreitas, Anderson ZanardiSarikov, AndreyFreitas, Edison Pignaton DeSarjas, AndrejFrémont, VincentSarkar, Nurul I.Freni, GabrieleSarkar, SaheliFrese, UdoSarkar, TapatiFreymueller, JeffSarlis, NicholasFrick, VincentSarma, RaktimFridlund, AlanSarolic, AntonioFridolin, IvoSarr, MoussaFriebe, MichaelSarraguça, MafaldaFriederich, FabianSarti, GiovanniFriedland, GeraldSartori, DanieleFriedman, JasonSarwar, YusufFriedman, KeithSasagawa, KiyotakaFriedrich, Christoph M.Sasaki, KenFriedt, Jean-MichelSasaki, MinoruFrisk, ThomasSasaki, TohruFritz, JanSasayama, TeruyoshiFrizzo Stefenon, StéfanoSassu, MauroFrnda, JaroslavSateriano, AdeleFröhlich, Antônio AugustoSathyachandran, Sanath KumarFronczak, MaciejSatler, MassimoFrontoni, EmanueleSato, HiroyukiFrovel, MalteSato, KoyaFryc, IrenaSato, ManabuFryskowska, AnnaSato, PriscilaFu, BoSato, RyutaFu, ChaoSato, TadatakeFu, ChengSato, TakaoFu, ChunyunSatrapinskyy, LeonidFu, HailingSattar, FarhaFu, HaiqiangSattar, FarookFu, HuixuanSattar, ShahramFu, JianyuSattarov, Robert R.Fu, KerenSaucedo, Nuvia M.Fu, LiSauer, KarenFu, LongshengSauer, NathalieFu, Lu-HuaSaura Lacárcel, José RamónFu, QiangwenSava, AlexandreFu, ShengSavaglio, ClaudioFu, ShuSaval-Calvo, MarceloFu, XiuwenSavastano, MatteoFu, YanweiSavazzi, StefanoFu, YuguangSavchuk, StepanFu, ZeyuSavi, PatriziaFudickar, SebastianSavino, AlessandroFue, KadegheSavino, StefanoFuentes, RamónSavkin, Andrey V.Fuentes, SigfredoSavkovic, BorislavFuentes-Pineda, GibranSawacha, ZimiFugetsu, BunshiSawaf, Mohamad Basel AlFuggetta, GiorgioSawicki, DariuszFujieda, NaokiSawicki, JacekFujigaki, MotoharuSawicki, PiotrFujii, SatoshiSaxena, NavratiFujimura, AtsushiSaxena, NeeteshFujinami, KaoriSaxena, ShefaliFujita, ToyohisaSayab, MohammadFujiwara, EricSayadi, HosseinFukuda, Jun-ichiSayago, IsabelFularz, MichalSayed Ahmed, Khaled RagabFulginei, Francesco RigantiSayem, Abu Sadat MuhammadFuller, AmeliaSayydi, NimaFumera, GiorgioSazio, Pier John AnthonyFunahashi, AkiraSbert, MateuFunari, RiccardoSberveglieri, VeronicaFunatsu, Beatriz M.Sbrollini, AgneseFung, Jimmy C. H.Scalenghe, RiccardoFung, Wai-KeungScalera, LorenzoFunk, RichardScalise, LorenzoFunka-Lea, GarethScampoli, PaolaFurferi, RoccoScandurra, AntoninoFurrer, FadriScapaticci, RosaFurtado, AntónioScappaticci, LorenzoFurtak, JanuszScaradozzi, DavidFurutani, RyoshuScaramuzza, MatteoFusco, AndreaScardulla, FrancescoFusilier, Donato HernándezScarelli, FredericoFuso, FrancescoScarlino, PasqualeFuss, Franz KonstantinScarpiniti, MicheleFutatsumori, ShunichiScarselli, GennaroFuzzo, DanielaŠčetar, MarioGabardo, ChristineSchabowicz, KrzysztofGabash, AoussSchäfer, Ihr JörgGabbar, Hossam A.Schäfer, KlausGaber, TarekSchäfer, MaximilianGabl, RomanSchaff, FlorianGablech, ImrichSchakel, WouterGabler, Hampton ClayScharber, MarkusGabriel, Jan PhilippScharoun Benson, SaraGabriel, Jose LuisSchaub-Meyer, SimoneGabriele, BarisSchauflinger, MartinGabriele, MagnaSchaum, AlexanderGabriella, TamburroSchazmann, BenjaminGabrielli, AlessandroScheid, Jennifer L.Gabrielli, FabrizioScheller, Frieder W.Gac, PawełSchena, EmilianoGacemi, DjamalSchenato, LucaGachet Paez, DiegoSchepers, JamesGadd, MatthewSchepler, KennethGaddam, AnuroopSchiano Lo Moriello, RosarioGade, RikkeSchiefler, Nivaldo T.Gadea-Gironés, RafaelSchiepp, ThomasGądek-Moszczak, AnetaSchilcher, UdoGadhoumi, KaisSchimalski, Marcos BeneditoGadjanski, IvanaSchimpe, MichaelGadsden, AndrewSchiopu, IonutGaffar, AshrafSchirhagl, RomanaGafurov, Artur M.Schirrmann, MichaelGagaoua, MohammedSchlachetzki, JohannesGagaoudakis, E.Schlegel, ChristianGaggero, TomasoSchlüer, Anna-BarbaraGai, KekeSchmid, GiovanniGai, ShaoyanSchmid, MaurizioGaiceanu, Habil. MarianSchmid, Moritz S.Gaiceanu, MarianSchmid, NataliaGaida, DanielSchmid, UlrichGaire, RajSchmidt, BernardGaitan, Vasile GheorghitaSchmidt, FranziskaGajda, JanuszSchmidt, Jan H.Gajic, DragoljubSchmidt, MarcusGajski, DubravkoSchmidt, SabineGal, Ionel-AlexandruSchmitt, CorinnaGál, TamásSchmitt, MartinGalantucci, Luigi MariaSchmitt, RobertGalan-Vidal, Carlos AndresSchmitt, ThierryGalas, RomanSchmitz, AnneGalati, GaspareSchmitz, NorbertGalatus, RamonaSchnakenberg, UweGalbács, GáborSchnaubelt, SebastianGaldames, Patricio A.Schneider, Fabio KurtGaldi, PaolaSchneider, ThomasGalea, ChristianSchnieder, SebastianGaleana Zapién, HiramScholkmann, FelixGalehdar, AmirScholl, Philipp M.Galinac Grbac, TihanaScholten, KeeGalindo, Velia Carolina OsunaSchormans, JohnGalinetto, PietroSchrag, GabrieleGalińska, BarbaraSchreiber, Karl UlrichGallacher, DavidSchreiner, WolfgangGallacher, KevinSchrempel, FrankGallagher, JohnSchröder, DietrichGallardo, LeonorSchultz, StephenGallas, QuentinSchultze, VolkmarGallay, MichalSchumacher, JensGallego, AntolinoSchumacher, JohannesGallegos-Funes, FranciscoSchumacher, ThomasGallelli, VincenzoSchütze, OliverGallina, AlbertoSchwartz, David EricGallo, PierluigiSchwarz, StefanGalluccio, LaurentSchwarz, Thomas J.E.Galo, MauricioSchwarzbach, PaulGaltarossa, AndreaSchweidler, SimonGalván-Tejada, Carlos E.Schweitzer, WolfGalvão, IvanSchwendel, ArvedGalve, Joan MiquelSchwenker, FriedhelmGamage, Duminda VidanaSchwertfeger, SörenGamba, Micaela TrogliaSchwesinger, NorbertGambi, EnnioSchwieger, VolkerGamboa, HugoSciarrone, AndreaGamec, JánScimemi, Giuseppe FilecciaGamella Carballo, MariaScionti, AlbertoGan, HaoScitovski, RudolfGan, MinSciuto, Emanuele LuigiGan, ShiyuScordo, AlessandroGan, Yong X.Scorza, AndreaGan, YuScotti, GiuseppeGanagina, IrinaScotti, MarcusGanau, MarioScribante, AndreaGancarz, MarekScurti, FedericoGanci, GaetanaSebastião, EmersonGandolfi, MarialuisaŠebek, JanGanesan, BaskaranSebestyen, GheorgheGang, MeiSebkhi, NordineGanga, AntonioSedaghat, NimaGangadharan, RajanSedlák, PetrGangopadhyay, AvijitSedlák, VladimírGanguli, SwetavaSedmidubský, JanGanovelli, FabioSedunov, AlexanderGanser, ChristianSee, ChanGanvir, AshishSee, Terrence Shie PingGao, Bo-CaiSeel, ThomasGao, Feng (China)Seeling, PatrickGao, Feng (UK)Seepersad, GarrettGao, HanSegal, NicolasGao, HangSegec, PavelGao, HongboSegers, LaurentGao, JianSeguel, FabianGao, JianbinSegura, José C.Gao, JianzhaoSegura-Garcia, JaumeGao, JingSehgal, RahulGao, JunqiSeichepine, FlorentGao, KaiSeidel, SallyGao, LeiSeifert, Ann-KathrinGao, LianruSeijo, Maria CarmenGao, LiboSeimeni, MariaGao, MengSeiphoori, AliGao, NanSekh, Arif AhmedGao, PeichaoSelezneva, MariaGao, QiSelim, HossamGao, RuipengSellami, AkremGao, ShangceSellitto, Miguel AfonsoGao, ShuoSelvaprabhu, PoongundranGao, Si-PingSelyanchyn, RomanGao, Wei (Chinese Academy of Sciences, China)Semaan, RichardGao, Wei (Guangdong University of Technology, China)Semeniuta, OleksandrGao, WenyueSeminati, ElenaGao, XiaofengSen Gupta, AnanyaGao, XiaomengSen, AnandoGao, XiaomingSen, DebarshiGao, XinghuaSen, SujatGao, ZhanSenay, GebrielGao, ZhiSenel, MehmetGao, ZhimingSenger, MoritzGao, ZhiweiSengupta, BidishaGao, ZongmeiSengupta, DebarunGaray-Jimenez, Laura I.Senhadji-Navarro, RaoufGaray-Vitoria, NestorSentana, IreneGarces, HugoSenyurek, Volkan YusufGarcia Alia, RubenSeo, Byung-KukGarcia Alonso, Jose ManuelSeo, Dong HyunGarcia Alvarez, ManuelSeo, Dong-HoanGarcía Cuenca, LauraSeo, DongmahnGarcía Galán, SebastiánSeo, EunilGarcia Gonçalves, Luiz MarcosSeo, HogeonGarcía Iglesias, DanielSeo, HwajeongGarcía Naya, José AntonioSeo, JahoGarcia Rodriguez, JoseSeo, MunkyoGarcía Sánchez, ManuelSeo, Sang HyunGarcia Souto, Jose AntonioSeo, ToruGarcía Tascón, MartaSeok, JunheeGarcia, Angel PontinSeok, SenhoGarcía, AntonioSeow, Chee KiatGarcia, CristobalSeow, CheekiatGarcia, EmiSepasgozar, SamadGarcia, GonzaloSequeira, AnaGarcia, JordiSequeira, JeanGarcia, Jose Manuel CanoSequeira, João SilvaGarcía, Jose Manuel MossiSerafín, VeronicaGarcía, Juan C.Serafín, VerónicaGarcía, MarceloSerajian, RezaGarcía, Miguel TorresȘerban, Bogdan CătălinGarcia, MissaelSerdean, FlorinaGarcia, Nuno M.Serdült, UweGarcía, SantiagoSeredin, OlegGarcía, TaniaSerenelli, LucaGarcía, VicenteSergeev, Aleksandr AleksandrovichGarcia, VictorSergi, Pier NicolaGarcía-Alcaraz, Jorge LuisSeriani, StefanoGarcia-Aracil, NicolasSerikov, ArkadyGarcia-Baños, BeatrizSerio, CarmineGarcía-Cabezón, CristinaSeritan, GeorgeGarcia-Constantino, MatiasSerna Barquera, Sergio AlonsoGarcía-Cortés, SilverioSernani, PaoloGarcía-Esparza, Juan A.Serodio, CarlosGarcia-Espinosa, EduardoSerrano, Arturo HernandezGarcía-Fuentes, Miguel Á.Serrano, Dolores R.Garcia-Garcia, LeonardoSerrano, Javier ToledoGarcia-Herrero, FranciscoSerrano, José IgnacioGarcía-Holgado, AliciaSerrano, NúriaGarcia-Lagos, FranciscoSerrano, SalvatoreGarcía-Lamont, FaridSerrano-Pertierra, EstherGarcía-Lastra, Juan MaríaSerrão, CarlosGarcía-Loygorri, Juan MorenoSerres, AlexandreGarcía-Macías, EnriqueSerry, MohamedGarcía-Martín, SheilaServa, LorenzoGarcía-Massó, XavierServadio, PierannaGarcía-Mateos, GinésServi, MichaelaGarcía-Miranda Ferrari, AlejandroServin, ManuelGarcia-Naya, Jose A.Sesé, JavierGarcía-Ordás, María TeresaSešek, AleksanderGarcía-Orellana, Carlos-javierSessa, SalvatoreGarcía-Peñalvo, Francisco JoséŠestak, IvanaGarcia-Perez, ArturoSestraș, PaulGarcía-Rubio, CarlosSetiawan, DediGarcía-Sánchez, José RafaelSeuwou, PatriceGarcía-Sánchez, TomásSeverino, AlessandroGarcía-Soidán, Jose L.Severino, RicardoGarcía-Unanue, JorgeSevic, DragutinGarcía-Vázquez, Juan-PabloSevieri, GiacomoGarcia-Villegas, EduardSevil, Hakki ErhanGardel, AlfredoSeybold, JonathanGardel-Vicente, AlfredoSeyedi, MirHojjatGardill, MarkusSeyyedhasani, HasanGargiulo, Gaetano D.Sfarra, StefanoGarmatyuk, DmitriySferrazza, CarmeloGarmendia, IñakiSgora, AggelikiGarmire, DavidSha, ChaoGarmire, ElsaShaaban, IbrahimGarnero, GabrieleShabatina, TatyanaGaroli, DenisShabbir, NomanGarramiola, FernandoShafiee, MahmoodGarrett, Joseph L.Shafiei Shiva, JavadGarrido Alzar, Carlos L.Shafiq, OmairGarrido, Juan E.Shafique, UmarGarrido, NeusShafiullah, G.M.Garrido-Castro, JuanShagdar, OyunchimegGarza, PaoloShah, AzizGarzia, FabioShah, MilinkumarGašinec, JurajShah, Nilam C.Gaspar, CristinaShah, PuravGasparini, LeonardoShah, Syed Afaq AliGašparović, MateoShah, Syed AzizGasparri, FabioShah, Syed BilalGasperis, Giovanni DeShah, Syed TariqGasser, ChristophShah, Syyed Adnan RaheelGastaldi, LauraShah, Vrutangkumar V.Gastaldo, PaoloShahaboddin, ShamshirbandGattass, MarceloShahian Jahromi, BabakGattone, Stefano AntonioShahid, Abdur Rahman BinGattringer, HubertShahid, ImranGaudioso, ElenaShahidi, RezaGaus, Yona Falinie A.Shahrestani, SeyedGauthreaux, SidneyShahriar, Md RifatGavazzi, BrunoShahriari, YaldaGavrilas, MihaiShahrour, IsamGavriloaia, GheorgheShahshahani, AmirhosseinGavrilova, MarinaShahtahmassebi, AmirrezaGay, SimonShahzad, AtifGayathri, Radhabai GopinathanShaik, Mohammed RafiGazda, PiotrShaikh, Salman AhmedGazquez Parra, Jose AntonioShakeel, HamzaGazzoni, MarcoShaker, GeorgeGbadebo, AdenowoShakthivel, DhayalanGe, DongdongShakya, Kabindra ManGe, HaiboShalaby, MohamedGe, Jun CongShalev, GilGe, LeijiaoShamanaeva, Liudmila G.Ge, XiaohuShamonin, MikhailGe, XiaoliangShamseldin, TamerGe, YufengShamshirband, ShahabGe, YunfengShamshiri, Redmond R.Ge, ZhiqiangShamszaman, Zia UshGebicki, JacekShan, GongbingGębicki, JacekShan, HongmingGebre, Biruk A.Shan, MaoGediminas, VaičiūnasShan, MingheGeiger, AlainShan, XinGeladi, PaulShanbhag, VigneshGelenbe, ErolShang, ChaoGeman, OanaShang, SonghaoGemba, Kay L.Shang, YongGeminiani, AliceShankar, MallikarjunGenerowicz, AgnieszkaShanmugam, BharanidharanGeng, KunShannigrahi, SusmitGeng, TaoShao, DangdangGeng, WeidongShao, HaidongGeng, YanquanShao, HaoGenge, BélaShao, LeiGenovese, Maria ErminiaShao, Pei-ChiGenovesi, SimoneShao, PengGentet, PhilippeShao, WeimingGeonea, Ionut DanielShao, XinxinGeorgakis, GeorgiosShao, XinxingGeorgantzinos, Stelios K.Shaohua, WangGeorge, SonyShaomin, ZhangGeorge, Thomas F.Shaqarin, TamirGeorge-Jaeggli, BarbaraSharaev, MaksimGeorgescu, Andrei-MugurSharafeldin, MohamedGeorgiadis, CharalamposSharif, MohammadrezaGeorgian, BadicuSharifan, HamidrezaGeorgiev, Daniel G.Sharma, BharatGeorgieva, Milena G.Sharma, GauravGeorgieva, TsvetelinaSharma, Gyani ShankarGeorgiou, TheodorosSharma, JyotsnaGeorgousis, GeorgiosSharma, MrigankGerasimova, Yulia V.Sharma, Piyush SindhuGerislioglu, BurakSharma, VishalGerke, OkeSharpe, Shaun M.Germanese, DanilaShashikumar, Supreeth P.Germanotta, MarcoShattal, Mohammad AbuGernaey, Krist V.Shavezipur, Mohammad K.Geronimo, Andrew M.Shaw, AnthonyGertych, ArkadiuszShaw-Cortez, WenceslaoGervasi, OsvaldoShe, Yong-XinGestal, MarcosSheard, SteveGewali, Utsav B.Sheen, Horn-jiunnGhaani, MasoudSheha, MoatazGhaderi, MohammadamirShehu, AmardaGhaderpour, EbrahimSheikh-Ahmad, JamalGhafar-Zadeh, EbrahimSheka, Elena F.Ghaffarian, SamanShekhani, HusainGhahari, Seyed AliShekhirev, MikhailGhani, ArfanShemer, AmirGhannad-Rezaie, MostafaShemer, LevGhannoum, AbdulRahmanShen, BinGhareh Baghi, ArashShen, BoGharehbaghi, KooroshShen, ChangqingGhassemlooy, ZabihShen, ChaoGhayvat, HemantShen, Chih-HsiungGhemari, ZineShen, ChongGheorghe, GrigorasShen, DazhongGheorghiu, AndreiShen, HongdaGherlone, MarcoShen, Hui-LiangGherm, VadimShen, JianliangGherman, BogdanShen, KaiGhiami, AmirShen, LiGhica, Mariana EmiliaShen, QiangGhica, Mihaela-VioletaShen, QingGhidelli, MatteoShen, RuiqingGhilain, NicolasShen, ShanpuGhisi, AldoShen, WeiGhita, MihaelaShen, XiGhodsi, MojtabaShen, YanfengGholizadeh, AsaShen, YangGhomian, TaherShen, YaochunGhorbanzadeh, OmidShen, YonghangGhorbel, EnjieShen, YuGhoreyshi, Seyed MohammadShen, YuanGhosal, SambuddhaShen, YuechengGhosh, AbhijitShen, ZhiqiGhosh, AbhishekShen, ZhiyuanGhosh, ArijitShenderovich, Ilya G.Ghosh, ChiranjitSheng, JiayiGhosh, DebanjanaSheng, JunGhosh, PradiptaSheng, XingGhosh, SouvikShenoy, AkhilGhosh, SubrataSheppard, ThomasGia, Tuan NguyenSherratt, R. SimonGiachetti, AndreaSheta, AlaaGiacomello, GiampieroSheta, BassemGiacomini, RenatoSheu, Jia-ShingGiacomozzi, FlavioSheu, Ruey-KaiGialampoukidis, IliasShevchenko, SergeyGialelis, JohnShevchik, Sergey A.Giammarchi, MarcoShevgunov, TimofeyGiancane, GabrieleShevtsov, SergeyGiannakeas, NikolaosShevtsov, Sergey N.Giannakis, KonstantinosShi, BinGiannakopoulos, TheodorosShi, Chuang (Beihang University, China)Giannakoulopoulos, AndreasShi, Chuang (University of Electronic Science and Technology of China, China)Giannelli, CarloShi, Hong-LingGiannetto, MarcoShi, HuaGiannini, PaoloShi, JiaGiannoccaro, IvanShi, JianhongGiannoutakis, KonstantinosShi, JianmaiGiarre, LauraShi, JieGiasin (Yasin), KhaledShi, JunboGibas, PiotrShi, KaifangGibbins, David R.Shi, KeGibert, JamersShi, LeiGiberti, AlessioShi, QiongfengGiberti, HermesShi, ShaohuaiGibiino, Gian PieroShi, TielinGideon J., GouwsShi, XiGidon, DoganShi, XiyuGiechaskiel, BarouchShi, YanlingGienko, GennadyShi, YushengGierens, KlausShi, ZhiguoGiergiel, MariuszShiaeles, Stavros N.Gierlak, PiotrShiau, Jaw-KuenGierz, LukaszShibata, NaokiGiger, Jean-ChristopheShibayama, JunGiggins, Oonagh M.Shieh, Chin-ShiuhGiglio, MarilenaShieh, Wann-YunGijón-Nogueron, GabrielShigaki, ShunsukeGil, Ana BelénShih, Ching-longGil, DavidShih, David Ching-FangGil, MiriamShih, TianyuanGilaberte, Raquel LacuestaShiki, Sidney BruceGil-Calvo, MarinaShill, Holly A.Gilland, DavidShilov, NikolayGillanders, Ross N.Shim, ChangbeomGillich, Gilbert-RainerShim, Duk-SunGillich, NicoletaShim, Jae HongGilliot, Jean-MarcShim, Jae HoonGilly, KatjaShim, Su-HyunGiménez, Jesus VicenteShim, Yoon-BoGimenez-Guzman, Jose ManuelShimabukuro, Milton HirokazuGimeno Blanes, Francisco JavierShimabukuro, YosioGinzburg, BorisShimada, KunioGiofrè, Vincenzo PasqualeShimizu, FlavioGiordan, DanieleShimizu, Flavio MakotoGiordano, RaffaeleShimizu, HisashiGiorgetti, AndreaShimizu, YouichiGiorgino, ToniShimizu, YukiGiorgio, IvanShin, Bo-SungGiosan, IonShin, Do HyoungGiovanni, CollodiShin, DongheeGiovannini, GiorgiaShin, JaeyoungGirão Silva, RitaShin, Jong WonGirbacia, FlorinShin, Jong-HoGirelli, Valentina AlenaShin, Joong HoGiri, ParitoshShin, Jung-AhGirolami, MicheleShin, Min ChulGirolamo-Neto, Cesare DiShin, NinaGislum, RenéShin, Soo YoungGitas, IoannisShin, Yun-hoGitis, ValeriShiraki, YoshifumiGiubilato, RiccardoShirazinejad, RezaGiudice, NicholasShirinskaya, AnnaGiudicepietro, FloraShirshin, EvgenyGiuffrè, Angelo MariaShiryayev, OlegGiuliano, AristideShkurinov, AlexanderGiuliano, RomeoShleev, SergeyGiungato, PasqualeShoaib, SultanGiusto, DanieleShokrolah Shirazi, MohammadGjevestad, Jon GlennShokrollahi, PeymanGjoreski, MartinShon, TaeshikGkonis, PanagiotisShorter, Kenneth AlexanderGkonis, Panagiotis K.Shorubalko, IvanGlas, DylanShoydin, SergeyGlaser, JohnShrestha, Anish PrasadGlatt, RubenShrestha, BhanuGlavas, HrvojeShrestha, BhushanGleich, DusanShrestha, MilanGlennie, CraigShrestha, RajuGligor, AdrianShrestha, RupeshGligoric, NenadShrestha, SantoshGlinkowski, WojciechShrestha, SudhirGlisic, BrankoShrirao, AnilGlonek, GrzegorzShtenberg, GiorgiGloria, AntonioShu, LinGlossop, NeilShuai, GaoGłowacki, TadeuszShubenkova, KseniaGłowacz, AdamShukla, MahendraGłowiński, SebastianShults, RomanGlukhova, Olga E.Shuxia, GuoGnade, BruceSi, LiangGnade, Bruce E.Siadat, MaryamGnanasekaran, KarthikeyanSiami-Namini, SimaGnesin, SilvanoSiani, Anna MariaGnilitskyi, IaroslavSichting, FreddyGning, AmadouSiddique, Muhammad AdnanGnyba, MarcinSiddiqui, Arooj MubasharaGobo, João Paulo AssisSiddiqui, Fahad ManzoorGockenbach, ErnstSiddiqui, RehanGodavarty, AnuradhaSiddiqui, Salman I.Godec, RankaSidorov, Denis N.Godina, RaduSiebenaler, ShaneGodinez Tello, RichardSieberth, TillGodinez, Humberto C.Sieciński, SzymonGodoy, Antonio José CalderónSiegel, Robert M.Goedemé, ToonSiegert, IngoGoetzendorf-Grabowski, TomaszSiemiątkowski, ZbigniewGoffredo, MichelaSieni, ElisabettaGoga, NicolaeSienkiewicz, ŁukaszGoggins, KatieSieradzki, RafałGogo, Elisha OtienoSierpiński, GrzegorzGogolin, GregSierra, Javier OrtuñoGoh, Tan LengSierra, Jesus EnriqueGoh, Wilson GohSierra-Castaner, ManuelGohari, SoheilSierra-Hernandez, Juan M.Goiffon, VincentSifuentes, ErnestoGoker, ParisaSigauke, CastonGola, ArkadiuszSigernes, FredGoldan, Amir H.Signore, GiovanniGoldberg, Kenneth A.Sigolaeva, Larisa V.Goldstein, PavelSikdar, ShirsenduGołębiowska, IzabelaSikirzhytski, VitaliGolewski, Grzegorz LudwikSik-Lanyi, CeciliaGoliatt, LeonardoSik-Lányi, CecíliaGolmohamadi, HessamSikora, JanGolnaz, HabibiSikorski, TomaszGolob, MarjanSikorski, WojciechGołofit, KrzysztofSikström, FredrikGolovnin, OlegSila-Nowicka, KatarzynaGolston, Levi M.Silber-Varod, VeredGomariz, SpartacusSilea, IoanGomes, AbelSileo, MariaGomes, Anderson S. L.Silien, ChristopheGomes, RahulSilina, YuliyaGomes, Ruan DelgadoŠiljak, HarunGomes, TeresaSiljeg, AnteGómez Salgado, JuanSillero, AmaliaGomez, Angel M.Silva Girão, PedroGómez, Clara M.Silva Junior, Carlos Antonio DaGomez, FedericoSilva, AdãoGómez, Juan Jesús RoldánSilva, Agnelo R.Gómez, LuisSilva, Alberto Jorge RosalesGomez, Ray (Ramon)Silva, Ana FilipaGómez-Andrés, DavidSilva, ArlindoGómez-Caballero, AlbertoSilva, CarlosGómez-Carmona, Carlos DavidSilva, Catarina L.Gómez-de-Gabriel, Jesús M.Silva, FilipeGomez-Donoso, FranciscoSilva, Gabriela VenturaGómez-Espinosa, AlfonsoSilva, IvanovitchGómez-Meire, SilvanaSilva, JoãoGómez-Pavón, Luz Del CarmenSilva, Lígia TorresGómez-Pulido, Juan A.Silva, Luiz Eduardo VirgilioGómez-Ruano, Miguel ÁngelSilva, MarianneGómez-Soberón, Jose ManuelSilva, NatassyaGomolka, ZbigniewSilva, Pedro Figueiredo E.Gonçalves Antunes, João FranciscoSilva, RuiGoncalves, AlexandreSilva, Saimon MoraesGonçalves, DanielSilva, SamuelGonçalves, Eder Mateus NunesSilva, SusanaGonçalves, GilSilva, Tiago AlmeidaGoncalves, Luís M.Silva-Castro, JhonGoncalves, PauloSilverio, VaniaGonçalves, PedroSilvestre, DanielGonçalves, Wesley NunesSilvestre, MiguelGoncharenko, YuriySilviu, FoleaGong, Cihun-SiyongSima, ChaotanGong, JamesSima, Felix NicolaeGong, ShuSimani, SilvioGong, TianxunSimanjuntak, Firman MangasaGong, XunSimenko, JozefGong, YichengSimeonov, VasilGong, ZhenfengSimian, DanaGong, ZhixiongSimic, MarkoGong, ZidanSimic, MilanGongora-Rubio, Mario RicardoSimion, Cristian E.Gonizzi Barsanti, SaraSimion, GeorgianaGonsalez Bueno, Camila GonsalezSimion, VioletaGonzalez Crespo, RubenSimmchen, JulianeGonzález García, MarianoSimmering, Jacob E.González Jiménez, Luis EnriqueSimon, BertrandGonzález Pérez, IsaíasSimon, Francois-XavierGonzalez, AlfredoSimon, SvenGonzález, CarinaSimone, AndreaGonzalez, FernandoSimone, GabrieleGonzález, FranciscoSimón-Martínez, CristinaGonzalez, JoaquinSimonot, LionelGonzález, Luis Ignacio LoperaSimorangkir, Roy B.V.B.Gonzalez, Pablo CorralSimoska, OljaGonzalez, RodrigoŠimšík, DušanGonzález-Camarena, RamónŠimunič, BoštjanGonzález-Cañete, Francisco JavierSina, AbuGonzález-Cortés, AraceliSinay, JurajGonzález-Díaz, DiegoSingh, AjitGonzález-Domínguez, Pablo IgnacioSingh, AmardeepGonzález-Jaramillo, Víctor HugoSingh, DhananjayGonzalez-Landaeta, RafaelSingh, JitendraGonzález-Landero, FranksSingh, KeshavGonzalez-Lopez, Veronica AndreaSingh, Keshav D.Gonzalez-Ortega, DavidSingh, NavragGonzález-Rocha, JavierSingh, Pankaj KGonzález-Romero, ElisaSingh, Rahul KumarGonzález-Sieira, AdriánSingh, RajaniGonzález-Sosa, EnriqueSingh, RanjanGonzález-Torres, AntonioSingh, Ritesh KumarGonzález-Vanegas, WilsonSingh, RiturajGonzález-Vila, ÁlvaroSingh, Sajal SagarGonzalo, JesúsSingh, SaurabhGonzalvez, Pablo RodriguezSingh, ShaktimanGoodchild, MartinSingh, Surya P.Goodin, Christopher T.Singh, UdayGoodney, AndrewSingh, UtkarshGopalakrishnan, KothandamSingh, VishalGopalsami, NachappaSingh, YogangGope, ProsantaSingha, SubhankarGöpfert, BeatSinha, AyanGorawska, AnnaSinha, RajuGorawski, MarcinSinha, Rashmi SharanGordon, StuartSinibaldi, AlbertoGordon, Wendy R.Siniscalchi, Sabato MarcoGoreham, ReneeSioma, AndrzejGórka, JacekSiozios, KostasGorodkiewicz, EwaSipos, LászlóGorpas, DimitrisŠipoš, MartinGórski, MarcinSipus, ZvonimirGosalbez, JorgeSiqueira Dias, José A.Gosiewski, ZdzisławSiqueira, Hugo ValadaresGostar, Amirali K.Sirbu, DanielaGotor, Alicia AyerdiSiregar, SyahrilGotta, AlbertoSirkeli, Vadim P.Gou, JianpingSirmaçek, BerilGoubej, MartinSirodoev, IgorGoudail, FrançoisŠiška, PetrGoudriaan, MarijeSisto, RenataGouglidis, AntoniosSitnik, RobertGould, MikeSittón-Candanedo, InésGounaris, AnastasiosSiu, Ho ChitGourinat, YvesSivaguru, MayandiGouton, PierreSivakumar, PoopalasingamGouwanda, DarwinSivarajan, SaravananGozdowski, DariuszSivasubramanian, JayaharGoździewska-Harłajczuk, KarolinaSiverns, JamesGrabham, NeilSivrikaya, FikretGrabska, EwaSjödahl, MikaelGrabska, JustynaSkaf, MartaGraci, ValentinaSkaggs, SheldonGracia-Ibáñez, VerónicaSkala, KaroljGradišek, AntonSkaria, SruthyGraf, Hans PeterSkarlatos, DimitriosGraf, MarkusSkiba, MartaGrafton, MilesSkibicki, JacekGragnani, Gian LuigiSkidanov, RomanGraichen, UweSkierucha, WojciechGramaglia, MarcoSkládal, PetrGrammalidis, NikosSkočaj, DanijelGrana, AnnaSkočir, PavleGrana, CostantinoSkoczeń, AndrzejGraña, ManuelSkoczylas, NorbertGranath, MatsSkodras, AthanassiosGranato, MarcoSkog, IsaacGrand, MaximeSkoko, ŽeljkoGranda, Juan CarlosSkonieczny, KrzysztofGrandin, TempleSkordas, EfthimiosGranelli, FabrizioŠkrbić, Biljana D.Granica, SebastianSkrickij, ViktorGranichin, Oleg N.Škrinjar, Jasmina PašagićGrassani, DavideSkruch, PawełGrassi, SilviaSkrzypczyński, PiotrGrasso, Alfio DarioSkwarek, VolkerGrau, AntoniSlaets, PeterGravina, RaffaeleSlama, Mohammed El AmineGray, Michael D.Slámová, MartinaGrazi, LorenzoSlaski, GrzegorzGraziani, AlbertoSlavcheva, MiroslavaGraziano, AlessandroSlavens, BrookeGrbić, RatkoSlavič, JankoGrebel, HaimSlawinski, CezaryGreco, AlessandroSławomir, CellmerGreen, PhilSleewaegen, Jean-MarieGreene, BarryŠlégr, JanGreenwald, RichardŚliwa, JoannaGreer, Adam T.Slobodian, PetrGregić, MajaSłodki, BogdanGregori, AlbertoSloma, MarcinGregori, AndrejSłota, RudolfGregurić, MartinSlouka, ChristophGreiner, DavidSługocki, ŁukaszGrekousis, GeorgeSmagowski, PiotrGrenouillet, FredericSmajic, JasminGric, TatjanaSmall, ChristopherGriffith, HenrySmaragdakis, CostasGriggio, FlavioŠmelko, MiroslavGrigoli, FrancescoSmetana, MilanGrigorszky, IstvánSmieja, MarekGrill, RomanSmith, BethGrilli, EleonoraSmith, DrewGrilo, António ManuelSmith, Eric P.Grilo, CarlosSmith, KevinGringoli, FrancescoSmith, Kirsten A.Grip, HelenaSmith, StewartGrippo, ValentinaSmith, ZacharyGrishin, MaximSmitham, PeterGrisi, MarcoSmołka, BogdanGrisso, RobertSmołka, KrystianGrobelna, IwonaSmuleac, LauraGrochla, KrzysztofSmulko, JanuszGröhl, JanekSmutny, VladimirGrolinger, KatarinaSmyl, DannyGroll, AndrewSmyth, Tim J.Gronauer, AndreasSnyder, DaltonGrondin, FrancoisSo, HongyunGrosch, TheodoreSo, JungminGrosicki, GregSo, Kwok KanGrosjean, GalienSoar, JeffreyGrosprêtre, SidneySoares Dos Santos, Marco P.Gross, BarrySoares Teles, ArielGrossi, MarcoSoares, FabrízzioGrosso, MassimilianoSoares, JoãoGroves, RogerSoares, Rafael IankowskiGroza, RobertSoares, VascoGruber, FlorianSobanjo, JohnGrubeša, SanjaSobaszek, MichałGrubić Kezele, TanjaSobieranski, Antonio CarlosGruev, ViktorSöbke, HeinrichGruić, IgorSobótka, MaciejGrulkowski, IreneuszSobotka, PiotrGruppioni, EmanueleSobron, IkerGryak, JonathanSobue, ShinichiGrygierek, JoannaSocas, RafaelGrzegorzek, MarcinSocha, TomaszGrzes, TomaszSochor, TomasGržić, RenataSocorro Leránoz, Abián BentorGrznár, PatrikSodhro, Ali HassanGrzybek, DariuszSöffker, DirkGu, ChenSofia, DanieleGu, ChunyangSohel Mahmud, S.M.Gu, FengshouSohn, InsooGu, FuqiangSohn, Jung WooGu, HaichangSoilán, MarioGu, KaiSo-In, ChakchaiGu, RentaoSoininen, Juha-PekkaGu, ShengfengSojka, MariuszGu, WentingSokol, ZbyněkGu, XiSokolov, OlegGu, XinyuSokolova, NadezdaGu, YaodongSola-Guirado, Rafael-RubenGu, YuSolak, KrzysztofGuadalupe-Grau, AmeliaSolari, LorenzoGuajardo, MarioSoldatenko, Sergei A.Gualandi, IsaccoSolé-Benet, AlbertGualtieri, LucaSoleimani, ManuchehrGuan, DongSoler, MariaGuan, KeSoler-Llorens, Juan LuisGuan, LeSolernou, AlbertGuan, LianwuSolhusvik, JohannesGuan, WeipengSoliman, Abdel-HamidGuan, XiaorongSoliman, HamdyGuan, ZhitaoSolimene, RaffaeleGuang, ZhengSolla, MercedesGuarneri, Giovanni AlfredoSollai, GiorgiaGuarnieri Calò Carducci, CarloSolle, DörteGuascito, Maria RacheleSolodov, IgorGubal, AnnaSoloviev, VladimirGubin, DenisSoltani, MadjidGuchhait, RekhaSoltaninejad, MohammadrezaGucma, MaciejSoman, RohanGucunski, NenadSoman, RuturajGudimetla, PrasadSomani, NikhilGudoshnikov, SergeySomeya, YuGüeita Rodríguez, JavierSomnath, SuhasGüemes, AlfredoSomorowska, UrszulaGuerboukha, HichemSon, DonghoonGuernouti, SihemSon, JiyoungGuerra, AnnaSon, JongsangGuerra, JorgeSon, JoonwooGuerra, JuanSon, JunggabGuerra, Maria GraziaSon, Pyo-WoongGuerra, RuiSon, Tae YunGuerra-Sanchez, GuadalupeSon, YoungdooGuerreiro, Bruno J. N.Song Nien, TangGuerreiro, Maria João SimasSong, AiguoGuerrero, JoséSong, BaoanGuerrero, JosechuSong, ChaolongGuerrero-Castellanos, FermiSong, ChengGuerrero-Ginel, José EmilioSong, CholhoGuerrieri, AntonioSong, FeiGuevara Lopez, Miguel AngelSong, GiltaeGui, JiangshengSong, HominGui, JieSong, HoubingGui, LinSong, JaeSeungGui, LinqingSong, Jin WooGui, RijunSong, JizhouGui, YilinSong, JuhaGuidi, FrancescoSong, KaichenGuidi, GabrielleSong, KangGuido, FrancescoSong, KechenGuido, GiuseppeSong, Kyoung-YoungGuido, Giuseppe PieroSong, MinkyuGuil, NicolasSong, SeungwonGuillén Gámez, Francisco DavidSong, ShaleiGuillén, MontserratSong, ShuangGuirao, BegoñaSong, SunghoGujski, MariuszSong, WeiGul, MustafaSong, WonhoGuldiken, RasimSong, XiaowenGullo, ParideSong, XiaoyuGuna, JožeSong, YafeiGunasekara, ChamilaSong, YangGunawardana, UpulSong, YaoGüngör, Cihan BerkSong, You-jinGünlü, OnurSong, YuGünther, MatthiasSong, YujaeGuo, BaofengSong, ZhanGuo, BaolongSong, ZhengyiGuo, BofengSoni, AswathiGuo, HangSonnessa, AlbericoGuo, HongzhiSonobe, ReiGuo, JianglongSoomro, KamranGuo, JianhuaSoos, GaborGuo, Jiun-inSophie, GrapeGuo, LeiSophocleous, MariosGuo, LiSorbello, GinoGuo, LingzhongSoriano, AntonioGuo, LongxiangSoriano, Jose A.Guo, LuSorici, AlexandruGuo, MengSorli, BriceGuo, QiandongSorokin, Boris P.Guo, QiangSorrentino, AlessandraGuo, ShuangzhuangSorsa, AkiGuo, WenzhongSortino, MarcoGuo, XiaoliangSosa González, Carlos JavierGuo, XiashengSośnica, KrzysztofGuo, XinSossa, HumbertoGuo, YiSotelo Monge, Marco AntonioGuo, YuanjunSoteriou, Georgios A. SoteriouGuo, YuntaoSotiriadis, PaulGuo, Zhan-HuSotner, RomanGuo, ZhouSoto, FernandoGupta, DeepaSoto, IgnacioGupta, MaanakSoto, PaulGupta, Munish KumarSoto, RobertGupta, SanjuSoto-Cámara, RaúlGupta, ShivamSoto-Mendoza, ValeriaGupta, SiddharthSoubervielle-Montalvo, CarlosGurau, VladimirSouihi, SamiGurban, Ana-MariaSoukup, RadekGurbuz, Sevgi ZubeydeSoulage, Christophe O.Gurka, MartinSoulis, KonstantinosGurrola, Luis Carlos GonzálezSousa, AntónioGurski, VolodymyrSousa, Armando JorgeGurupur, Varadraj PrabhuSousa, Bruno MiguelGuselnikova, Olga AndreevnaSousa, DanielGu-Stoppel, ShanshanSousa, Daniel J.Gut, KazimierzSousa, Fernando R.Gutiérrez Guerrero, Jóse MiguelSousa, João M.C.Gutiérrez Reina, DanielSousa, Joao MiguelGutierrez, AlvaroSousa, JoaquimGutierrez, GuillermoSousan, SinanGutierrez, JonSouza, Israel Donizeti DeGutiérrez, Juan ManuelSouza, Jéssica Sena DeGutierrez, Miguél ÁngelSovic, AnaGutierrez, SebastianSoylu, AhmetGutiérrez-Avilés, DavidSpaanenburg, LambertGutiérrez-Martinez, José MaríaŠpaček, JanGutsch, SebastianSpachos, PetrosGuy, JonathanSpagnol, SimoneGuyonneau, RémySpanakis, Emmanouil G.Guzik, AgnieszkaSpanedda, MariaGuzmán, Borja GenovésŠpanič, ValentinaGuzman, MarceloSpanu, AndreaGuzman-Chavez, Ana DinoraSpasojević, SofijaGuzman-Sepulveda, Jose R.Specht, CezaryGyurcsányi, RobertSpecht, MariuszHa, Don-HyungSpecogna, RubenHa, MinjeongSpencer, NicholasHaack, BarrySpengler, Anderson WedderhoffHaba, Cristian-GyozoSperanza, GiorgioHaber, RodolfoSperlì, GiancarloHabib, AdnaneSpiehl, DieterHabib, CarolSpigulis, JanisHabib, KhaledSpiliotopoulos, DimitrisHabibi, HamedSpiller, DarioHabibović, MirelaSpinazzè, AndreaHabisreuther, TobiasSpinelle, LaurentHabrych, MarcinSpinelli, LorenzoHabuda-Stanić, MirnaSpinsante, SusannaHachaj, TomaszSpitschan, ManuelHachour, SamirSpivak, ArturHadaś, TomaszSpivak, DavidHadas, ZdenekSpohr, Klaus MichaelHaddad, MalikSpoladore, DanieleHaddadi, AbbasSpolaor, FabiolaHaddadi, Seyyed ArashSpolaor, SimoneHadi, Muhammad UsmanSportelli, Maria ChiaraHadjichristofi, GeorgeSprague, Michael A.Hadjipapas, AvgisSpringer, ShmuelHaerinia, MohammadSprong, Matthew EvanHafer, Jocelyn F.Spyrou, EvaggelosHagem, RabeeSquires, AndrewHaghi, MostafaSraml, MatjazHaghighi, Pari DelirSreckovic, Vladimir A.Haghshenas, Sami ShaffieeSreedharan, SreejeshHaghshenas-Jaryani, MahdiSreejith, Kamalalayam RajanHagmüller, MartinSreekanth, Kandammathe ValiyaveeduHahn, MichaelSreekumari, PrasanthiHaider, FasihSreenilayam, SitharaHaider, Mohammad RafiqulSrikanthan, ThambipillaiHaigh, AnthonySriram, RashmiHaitjema, HanSrivastava, GautamHajduch, GuillaumeSrivastava, ShubhamHaj-Hosseini, NedaSrivastava, VishwasHajiyavand, AmirSroka, RyszardHájovský, RadovanSrpak, DunjaHåkansson, JohanStabauer, MartinHakula, HarriStacewicz, TadeuszHakulinen, JaakkoStachiv, IvoHalicek, MartinStachlewska, IwonaHalkon, BenStachowiak, DorotaHall Barbosa, Carlos RobertoStaderini, EnricoHallemans, AnnStaerz, AnnaHallik, LeaStagni, RitaHallil, HamidaStaicu, TeodoraHalunga, Simona VioricaŠtajduhar, IvanHalvorsen, KjartanStamate, EugenHam, SuyunStamatelos, Anastassios M.Hamad, DenisStamatescu, IuliaHamadi, RabyStamenkovic, ZoranHamaguchi, ToyohiroStamm, AndyHamaza, SaluaStamnes, KnutHambly, BrettStamou, AdamantiaHamdani, AriStan, MirunaHamedpour, VahidStana, Anca-DanielaHameed, Ibrahim A.Stanaćević, MilutinHameed, MusabStančin, SaraHamelin, BenoitStanciu, Mariana DomnicaHamidouche, MouradStanculescu, MarilenaHamidullah, MuhammadStaniec, KamilHamilton, AndrewStaniek, MarcinHammer, RenéStaniszewska, MagdalenaHamon, MorganStaniszewski, MichałHamza, RafikStankovic, LinaHamza-Cherif, ArslaneStankovic, VladimirHan, Chin-ChuanStanula, ArkadiuszHan, ChongStarecki, TomaszHan, CongzhengStarke, EricHan, DezhiStarr, ThomasHan, Dong SeogStaš, MichalHan, DongsooStasa, PavelHan, Dong-WookStassi, StefanoHan, FeiStastny, PetrHan, FengtianStateczny, AndrzejHan, GeStateczny, Andrzej EugeniuszHan, Guo-ChengStaten, Paul W.Han, Hyoung-SuStavinoha, Peter L.Han, JinsongStavrakis, ModestosHan, JungheeStavrou, VasilisHan, KeStavroulakis, Georgios E.Han, LeiStefan, Markiewicz JakubHan, MengStefan, OvidiuHan, NingStefańczyk, MaciejHan, PengStefani, AlessioHan, QingwenStefanov, KonstantinHan, RuisongSteffen, ThomasHan, SangwookSteffert, TonyHan, Seong-IkSteglich, PatrickHan, TaeheeStein, AlfredHan, XianhuaSteinbauer, MiloslavHan, XiaoSteinfeld, NiliHan, XiufengSteingartner, WilliamHan, XiuyouSteinke, MichaelHan, YiweiStelitano, DarioHan, YongmingStemler, ThomasHan, Young-GeunStencel, MarekHan, YuxingStenman, MalinHanavan, Ryan P.Stepanov, Oleg A.Hancock, Craig MatthewStepenko, SerhiiHandsfield, GeoffreyStéphane, KemkemianHangan, AncaStephanedes, YorgosHanke, StenStephen, BruceHannu, JariStephens, Andrew FHänsel, AndreasStepien, JacekHansen, ClintStepien, KrzysztofHansen, JohnStępniak, KatarzynaHansen, Mark F.Šter, BrankoHao Jian Zhong, EmilySterca, AdrianHao, FeiStereńczak, KrzysztofHao, NanjingStergiou, AlexandrosHao, YongqiangSternberg, AndrewHao, ZhaoStetter, RalfHao, ZhenhuaStevan Jr., Sergio L.Haq, MuhammadAziz UlStevens, Martin J.Haque, Sardar AnisulStewart, Brian G.Hara, MasanoriStewart, RebeccaHarada, YukinoriStewart, Terrence C.Haras, MaciejStewart, ThomasHarding, ChrisSthal, FabriceHarezlak, JaroslawStiburkova, BlankaHarezlak, KatarzynaStielkowski, WadimHarik, El Houssein ChouaibStiharu, IonHariri-Ardebili, Mohammad AminStipanicev, DarkoHarizanov, StanislavStiros, Stathis C.Harnois, MaximeStobiecka, MagdalenaHaro, GonzaloStoean, CatalinHarris, NickStoean, RuxandraHarris-Birtill, DavidStoia, Dan IoanHarrison, AlexanderStoica, Adrian-MihailHarrison, WillieStoican, FlorinHartley, CarolineStojanovic, BrankaHartman, MariuszStojmenova, KristinaHartmann, JensStokely, Thomas D.Hartnagel, HansStolarczyk, AgnieszkaHartung, JensStolfi, Daniel H.Harvey, RichardStolov, Andrei A.Harvey, ScottStolz, RonnyHasan, Khondker ShajadulStolzenburg, FriederHasan, MahmudulStoop, Ralph L.Hasan, Md MehediStopic, SreckoHasanian, MostafaStorch, TobiasHasegawa, KeisukeStork, MilanHasegawa, Makoto (Chitose Institute of Science and Technology, Japan)Stornelli, VincenzoHasegawa, Makoto (Nagahama Institute of Bio-Science and Technology, Japan)Stortini, AngelaHashemi-Beni, LeilaStoyanov, BorislavHashimoto, Ken-yaStoynov, ViktorHashishin, TakeshiStraka, OndřejHashizume, HiromichiStrakowski, MarcinHaskell-Dowland, PaulStraßer, TorstenHassan, Ahnaf RashikStratos, DavidHassan, EmadeldeenStraub, JeremyHassan, Sammer-ulStrauss, AlfredHassanalian, MostafaStreimikiene, DaliaHassanipour, FatemehStrelnikova, IrinaHassapis, GeorgiosStrianese, MariaHaßlinger, GerhardStrimbu, BogdanHassui, AmauriStrimbu, Bogdan M.Hatami, MohsenStrisciuglio, NicolaHatami, NimaStrle, DragoHattersley, JohnStrnad, RadekHattori, ToshiakiStroh, AlbrechtHattori, YoshihideStrokina, NataliyaHaupt, MichaelŠtroner, MartinHaušild, PetrStroulia, EleniHausman, SławomirStrumillo, PawelHaut, Juan MarioStrungaru, Stefan-AdrianHavemann, StephanStrušnik, DušanHavlík, JanStruzikiewicz, GrzegorzHavran, LudekStumpe, JoachimHawbani, AmmarŠturala, JiříHay, IanSturari, MircoHayar, AbdullahSturdivant, RickHayward, Richard DavidStütz, PeterHazdra, PavelStyers, Diane M.He, BingweiSu, BaofengHe, ChengSu, BingHe, ChengbingSu, ChunyiHe, DapingSu, Chun-yiHe, DenghuiSu, HangHe, DongfengSu, HongboHe, FangningSu, Pi-GueyHe, GuangxinSu, RuoyuHe, HongSu, Ting-LiHe, HuSu, Tung-ChingHe, HuijingSu, Wen-HaoHe, JianjunSu, XinHe, JinSu, YanjieHe, JinxinSu, YishanHe, KaifeiSu, YuanjieHe, LimingSu, ZhongHe, MaggieSuárez Sarmiento, AlvaroHe, MingyiSuarez, AlvaroHe, PingSuarez, ElisabetHe, ShaomingSuárez, Victor Manuel GonzálezHe, ShimingSubbaraman, HarishHe, Suining (China)Subber, WaadHe, Suining (USA)Subedi, SantoshHe, WeiSubirats, LaiaHe, XianqiangSublime, JérémieHe, XiaoxingSubramaniam, RamasamyHe, XipingSucharda, OldrichHe, XuSuchorab, ZbigniewHe, Xue-FengSuciu, AlinHe, YangyangSuciu, Bogdan AndreiHe, YaqianSuciu, GeorgeHe, YihaiSuddapalli, Chaitanya KumarHe, YuanSudmanns, MartinHe, YunSuea-Ngam, AkkapolHe, YunzeSugano, MasashiHe, ZhenyuSugawa, KosukeHe, ZhiweiSugimori, HiroyukiHeadland, DanielSuh, Young SooHeadley, WilliamSuhm, Martin A.Heal, MathewSui, DongHeberger, KarolySui, HaigangHégarat-Mascle, Sylvie LeSui, TingtingHeijnen, Petra W.Sui, XiaohongHeikkilä, Anu M.Suir, GlennHeikkilä, TapioSujecki, SlawomirHeikkinen, Jani ErikSuk, TomášHeim, FredericSukeri, AnandhakumarHeimfarth, TalesSukhov, VladimirHein, JoachimŠuligoj, FilipHeins, BradleySulikowski, PiotrHeirich, OliverSuliman, AhmadHeise, BettinaSułowicz, MaciejHeise, Herbert MichaelSulyman, Ahmed IyandaHejda, JanSum, Yee LoonHeld, PhilippŠumak, BoštjanHelebrandt, PavolSumbwanyambe, MbuyuHellbrück, HorstSumetsky, Michael (Misha)Hellwagner, HermannSumida, PauloHelmi, GhanmiSumma, SusannaHempel, MartinSun, BaoqiHenarejos, PolSun, BaoqingHendawi, AbdeltawabSun, BingHenderson, RobertSun, ChaoliHendijanizadeh, MehdiSun, ChengmingHendrick, PatrickSun, ChuangHendrie, JamesSun, DaHendrikse, HarrySun, DanfengHenesey, Lawrence E.Sun, DaweiHenriques, Felipe Da RochaSun, Da-ZhiHenry, DavidSun, DeGuiHenry, ManusSun, EnjiHensel, SebastianSun, FangminHenze, MartinSun, FenggangHeo, SeongkookSun, FumingHeo, Yong-SeokSun, GuanghaoHeo, Yun JungSun, GuangyuHer, Shiuh-ChuanSun, HaixinHeras, AránzazuSun, HanjunHeras, JónathanSun, HeHerbin, StéphaneSun, HongHerbut, AnetaSun, HongbinHerbut, PiotrSun, HuHermann, NunoSun, HuafeiHermann, TobiasSun, HuchengHermawan, AnggaSun, JiaHermoso-Orzáez, Manuel JesúsSun, JiaminHernández Arriaga, Marco VinicioSun, JianboHernández Creus, AlbertoSun, JiazhenHernández Del Olmo, FélixSun, JiguangHernández Sosa, José DanielSun, JingxuanHernández, AlfredoSun, JinhuaHernández, David NaranjoSun, JinpingHernandez, Eusebio EduardoSun, JinweiHernandez, LeobardoSun, KaiHernandez, LuisSun, Kien-WenHernández, Margarita GonzálezSun, LanxiangHernandez, MonicaSun, Lei (China)Hernandez, SarahSun, Lei (USA)Hernández-Callejo, LuisSun, LiHernandez-Escobedo, QuetzalcoatlSun, LiBoHernandez-Guzman, VictorSun, LingHernández-Hernández, José LuisSun, MengHernandez-Hierro, JoseSun, Ming-JieHernández-Negrete, OfeliaSun, MinhongHernández-Pérez, IvánSun, PengHernández-Ramos, José LuisSun, QuansenHernandez-Vazquez, Jesus-MariaSun, RuiHerndl, MarkusSun, RuopengHernes, MarcinSun, ShangpengHerod, MorganSun, Shih-WeiHéroux, PaulSun, ShilongHerppich, WernerSun, TaoHerraiz, Joaquin L.Sun, Tien-LungHerrera, ManuelSun, WeiHerrera, Pedro JavierSun, WeidongHerrera-López, Enrique J.Sun, WeiweiHerrera-May, Agustin L.Sun, WenHerrera-Peco, IvánSun, XiankaiHerrero, AliciaSun, XiaodongHerring, StevenSun, XuanHerring, SusanSun, YanchaoHerrmann, J. MichaelSun, YanhuaHerth, EtienneSun, YaoHeshmati-alamdari, ShahabSun, YuanxiHespel, LaurentSun, YunfuHess-Dunning, AllisonSun, Yun-LuHesselbach, XavierSun, YunxuHesselbarth, AnjaSun, YushanHeusse, MartinSun, YuxiangHewitt, AndrewSun, ZheHewson, MichaelSun, ZhengHeydari, M. HossainSun, ZhengxingHeyduk, AdamSun, ZhixinHianik, TiborSun, ZhiyongHibara, AkihideSun, ZhongchangHicke, KonstantinSun, ZihengHidalgo López, José AntonioSunar, UlasHidalgo, ArturoSundar, KaarthikHidalgo, Jose Manuel SotoSundararajan, AdityaHieyong, JeongSundararajan, KalaivaniHigo, AkioSung, I-HsinHiguti, Ricardo TokioSuo, GuoquanHildmann, HannoSuppa, AntonioHilfer, MichaelSuppan, LaurentHill, DanielSurace, CeciliaHill, David J.Suresh, Mahima AgumbeHill, Ethan ColeSurian, DidiHill, Lawson DrewSurmeneva, MariaHiller, JochenSurmick, DavidHiller, MatthiasSurovy, PeterHillier, SusanSurowka, ArturHilt, AttilaSurugiu, Maria ClaudiaHimpel, MichaelSuryadevara, Nagender KumarHinman, SamuelSuryadi, FransiscusHino, HideitsuSurženkov, AndreiHinostroza, IsraelSusek, WaldemarHirdaris, SpyrosSusnea, IoanHirmer, PascalSussman, DafnaHirofumi, NogamiSuta, MarkusHiromoto, RobertSutin, AlexanderHirose, YutakaSutlief, ArinHirsch, MatíasSutradhar, ManasHirsch, ThomasSuvocarev, KosanaHitoshi, TsunashimaSuvorova, SofiaHjelme, Dag RoarSužiedelytė-Visockienė, JūratėHjelseth, EilifSuzuki, ArataHladek, DanielSuzuki, DanielaHlaváč, Libor M.Suzuki, SeiyaHlaváčová, ZuzanaSuzuki, YasushiHmila, MariemSuzuki, YoshioHnilicka, FrantisekSuzurikawa, JunHo, Aaron H.P.Švanda, MilanHo, Jun-WonŠvantner, MichalHo, Siu WaiSvehla, DrazenHo, Tan-JanSvensson, AnnHo, Ya-LunSvete, AndrejHoang, Thanh VanSvidró, József TamásHoath, StephenSvihlık, JanHobbs, RichardSwacha, JakubHoblos, GhalebSweeney, DanielHocenski, ZeljkoŚwiątek, JerzyHodge, VictoriaŚwiderski, WaldemarHoffman, JasonŚwiercz, MirosławHoffmann, AndreasŚwiercz, RafałHoffmann, DanielŚwietlicka, IzabelaHoffmann, GundulaŚwirniak, GrzegorzHoffmann, Markus M.Świt, GrzegorzHoffmeister, DirkSyafrudin, MuhammadHöft, MichaelSyczewska, MalgorzataHogsette, Jerome A.Sydänheimo, LauriHogstad, Bjørn OlavSyed Haider, AbbasHoh, Hsin-jenSyed-Ab-Rahman, Sharifah FarhanaHoła, JerzySysoev, VictorHölbl, MarkoSzabo, AlexandreHoldengreber, EldadSzabo, LorandHoldsworth, CloviaSzabó, SzilárdHolé, StéphaneSzabó, ZoltánHo-Le, Thao P.Szabolcsi, RóbertHolik, FilipSzafarczyk, AnnaHoller, StephenSzajerman, DominikHolobaca, Iulian HoriaSzámel, LászlóHolonec, RodicaSzatyłowicz, JanHolst, ChristophSzczegielniak, TomaszHolst, NielsSzczepański, MarekHolub, MichalSzczerska, Malgorzata JędrzejewskaHolubek, RadovanSzczęsna, AgnieszkaHonaker, Lawrence W.Szcześniak, PawełHonari, Mohammad MahdiSzczuko, PiotrHondongwa, DonaldSzczurek, AndrzejHoneine, PaulSzebényi, GáborHong, CharmgilSzegletes, ZsoltHong, Cheng-YihSzékács, InnaHong, ChengyuSzelag, AdamHong, DanfengSzeleszczuk, ŁukaszHong, Jin B.Szénási, SándorHong, JunSzentgyörgyi, HajnalkaHong, MenSzermer, MichalHong, Nien-MingSzeto, GraceHong, PeilongSzewczyk, RomanHong, SanghyunSzewrański, SzymonHong, SugwonSziebig, GaborHong, TaoSzkaliczki, TiborHong, Wei-ChiangSzombara, StanisławHong, Youn-SikSzoszkiewicz, RobertHong, ZhenSzperlich, PiotrHong, ZihanSzpica, DariuszHongliang, LiangSzucs, VeronikaHönnicke, Marcelo GonçalvesSzulczyński, BartoszHonório, Leonardo De MelloSzultka, SewerynHoog Antink, ChristophSzumińska, DanutaHooper, GarySzumski, OskarHopper, RichardSzwajka, KrzysztofHorák, JakubSzwedziak, KatarzynaHordacre, BrentonSzwoch, MariuszHoremuz, MilanSzymanik, BarbaraHorender, StefanSzymborski, TomaszHori, MaiyaSzyszkowicz, MieczyslawHorikawa, IzumiTa, Bao QuocHorla, DariuszTabaa, MohamedHorn, BertholdTabaka, PrzemyslawHorne, MalcolmTabar, Yousef RezaeiHorng, Mong-FongTabassum, ShawanaHorng, Shi-JinnTabatabaei-Jafari, HosseinHorng, Tzyy-ShengTabatabaeipour, MortezaHorri, NadjimTaboada, FernandoHorrillo Güemes, CarmenTaborri, JuriHorst, FabianTabuenca, BernardoHortschitz, WilfriedTada, ShigeruHorvai, GeorgeTaddeo, SophieHorvath, DenisTadesse, Girmaw AbebeHorváth, JuditTadesse, MelkieHorvath, RobertTadyszak, KrzysztofHorvath, TomasTae, Han-ShenHošek, JanTaebi, AmirtahaHosek, JiriTaetz, BertramHoshen, YedidTaffoni, FabrizioHoshiba, KotaroTagarev, TodorHosomi, TakuroTagawa, ToshioHossain, AzadTaghavi, NazitaHossain, BelayatTaghizadeh-Mehrjardi, RuhollahHossain, M. NurTagliaferri, DarioHossain, MahtabTagliapietra, LucaHossain, Md ZakirTaguchi, YoshihiroHossain, Md. FarukTahara, HironobuHossain, Mohammad JobayerTahara, YusukeHosseinzadeh, MehdiTaheri, AbbasHosu, OanaTai, HuilingHou, LiqunTai, Li-ChiaHou, Tsung-ChinTaiar, RedhaHow, Meng-LeongTaillade, FrédéricHoward, Heidi R.Taishi, NakamuraHowcroft, JenniferTakač, IztokHowlader, Matiar RTakaffoli, MahdiHozman, JiriTakahashi, ShuntaroHożyń, StanisławTakaki, ManabuHraníček, JakubTakano, HidekazuHristoforou, EvangelosTakaya, TomohisaHsia, Kuo-HsienTakayama, OsamuHsiao, Hsi LienTakeda, RyoHsiao, Hsu-ChunTakeda, ShigekiHsiao, Hui-HsinTakewaka, SatoshiHsieh, Chia-YehTakizawa, HotakaHsieh, Ching-luTakyi, GabrielHsieh, Hung-LinTalaat, AhmedHsieh, Tung-HsunTalagala, Priyanga DiliniHsieh, Yu-chungTalakoub, OmidHsi-Jen James, YehTaler, JanHsin, Kun-YiTalla, VamsiHsiu, HsinTallarico, MarcoHsu, Che-ChuanTalleb, HakeimHsu, Chen-Chien JamesTama, Bayu AdhiHsu, Chien-ChangTamagnone, MarioHsu, Ching-ChiTamari, SergeHsu, Feng ChiTamarin, OllivierHsu, Heng-TungTamas, Razvan D.Hsu, Huan-HsuanTamazin, Mohamed E.Hsu, Li-TaTamba, Bogdan IonelHsu, Ming-WeiTamburri, Damian A.Hsu, Sam H. Y.Tamilia, EleonoraHsueh, Ya-HsinTammaro, UmbertoHu, Bin (Huazhong University of Science and Technology, China)Tammemae, KalleHu, Bin (Lanzhou University, China)Tamura, HirokiHu, BoyiTamura, KosukeHu, CaihongTamus, Zoltán ÁdámHu, ChuanTan, BoHu, Fang Q.Tan, Chee PinHu, FeiTan, ChekfoungHu, GaoTan, Chin YawHu, GuoliangTan, Chiu C.Hu, HanTan, IsabellaHu, HaofengTan, JeanneHu, HongliTan, JingHu, HongpingTan, JinhuaHu, HuangshuiTan, JunxiangHu, JiaTan, Kuang-HsiungHu, JieTan, KunHu, JingtianTan, NingHu, JunhuiTan, Rex XiaoHu, LilyTan, ShengHu, MaocongTan, Syh-YuanHu, MenghanTan, Yew TeckHu, NingTan, YihuaHu, QijunTan, YisongHu, QinTanabe, KatsuyukiHu, ShengshanTanabe, MasayukiHu, ShunTanackov, IlijaHu, SijungTanaka, HiroshiHu, SimonTanaka, JiroHu, TangaoTanaka, MasayukiHu, TianjiangTanaka, ShigehoHu, TianyuTanaka, ToshiyukiHu, XiangyunTananaiko, OksanaHu, XiaosongTanasiev, VladimirHu, XiaosuTanasova, MarinaHu, XiuqingTänavots, AloHu, XukeTang, CenHu, YangTang, FeiHu, YunpuTang, GuanglinHu, Zhan-YiTang, HeshengHu, ZhaozhengTang, HongHu, ZhenzhongTang, JinjunHua, WenjianTang, JunHuan, Rong-huaTang, LiqunHuang, AnpengTang, MingHuang, AnthonyTang, QiangHuang, BaoqiTang, SuhuaHuang, ChangTang, WeiHuang, ChaoranTang, WenlongHuang, ChaosongTang, XiaojunHuang, Chih-ChingTang, XiyuanHuang, Chih-YungTang, XuHuang, Chiou-JyeTang, YandongHuang, ChuanheTang, YingganHuang, Chung-ChiTang, YongchuanHuang, Chung-JenTang, YunchaoHuang, Chun-YingTang, ZhichuanHuang, Chun-YuanTang, ZhifengHuang, FamingTangirala, Venkata Krishna KarthikHuang, FangTango, FabioHuang, Guang-BinTanner, NathanHuang, GuanwenTanno, TakenoriHuang, GuoshengTao, FeifeiHuang, HailongTao, JianchengHuang, HaipingTao, JinHuang, HaocaiTao, JunHuang, Helen J.Tao, Kai (China)Huang, HuayuTao, Kai (Israel)Huang, HuiTao, LeiHuang, HungJiTao, MingHuang, HuolinTao, MingliangHuang, JennyTao, TianyouHuang, Jheng-JiaTao, XiaofengHuang, JianqiuTao, XuyuanHuang, JiaruiTaousser, Fatima ZohraHuang, JieTarallo, AndreaHuang, JingfengȚarălungă, DragoșHuang, JiyanTaramasco, CarlaHuang, Jun (China)Tarasenkov, Mikhail V.Huang, Jun (UK)Tarasiuk, HalinaHuang, LeiTarasov, AlexeyHuang, LimingTarasov, Anton S.Huang, LvwenTaravat, AlirezaHuang, MingTaravati, SajjadHuang, NantianTarca, RaduHuang, QiangTarczewski, RomualdHuang, QihuanTardif, Pierre MartinHuang, QijunTarnapowicz, DariuszHuang, QinganTarnita, DanielaHuang, QinghuaTartaglione, EnzoHuang, QiyaoTasca, FedericoHuang, RongyongTashakori, ShervinHuang, ShengxiTashev, IvanHuang, Shih-ChangTashpulatov, Sherzod N.Huang, Shih-ChiaTatar, ErdincHuang, ShipingTate, DanielHuang, ShourenTatlas, Nicolas AlexanderHuang, Shyh-ChinTatu, Serioja OvidiuHuang, SonglingTatullo, MarcoHuang, SunanTausif, MuhammadHuang, Tzu-HaoTavakkoli, AlirezaHuang, WeichuanTavakolian, KouhyarHuang, WeiminTavares, Tiago FernandesHuang, Wen ChangTavolato, PaulHuang, WenbinTayara, HilalHuang, XianheTayeb, ShahabHuang, XiaolinTaylor, AndyHuang, XinboTaylor, CianHuang, YanTaylor, ClarkHuang, YingTaylor, SimonHuang, YiqingTchanche, Bertrand FankamHuang, YishuoTchekalarova, JanaHuang, YuTcherniak, DmitriHuang, Yu-HsiTchórzewska-Cieślak, BarbaraHuang, YulongTebekaemi, EniyeHuang, Yung-FaTecpoyotl-Torres, MargaritaHuang, Zhaoran (rena)Tedesco, AnnaritaHuang, ZhaorongTedesco, SalvatoreHuang, ZhipeiTeh, Pin ShenHuda, Md. NazmulTeh, Ying KhaiHudak, RadovanTehrani, KambizHuertas, César S.Tehrani, ZariHueske-Kraus, DirkTeimouri, NimaHughes, BenTeixeira, FernandoHughes-Riley, TheodoreTeixeira, Francisco CuradoHuguet, Silvia SoriaTeixeira, João M.C.Huh, JungwonTeixeira, Marco AntonioHuh, Jun-HoTeixeira, Marcos F.S.Hui, Teo TeeTeixeira, SofiaHui, YilongTeixidó Ullod, TeresaHuignard, Jean-PierreTekeoglu, AliHulsey, LeroyTekes, CoskunHung, Jeih-weihTello, JavierHung, JohnTello-Oquendo, LuisHung, Jui-chungTemplin, TomaszHung, Li-FangTenace, ValerioHung, Pham PhuocTeng, HualiangHung, Shao-KangTeng, Liong SzeHunova, IvaTeng, PengHuo, Lian-ZhiTengfei, WuHuo, LinshengTenhunen, MirjaHuo, YimingTennina, StefanoHuo, YongqingTenreiro, MiguelHuotari, MattiTeo, Ee JinHuptych, MichalTeodor, RusuHupy, JosephTeodorescu, FlorinaHur, ShinTepljakov, AlekseiHurst, WilliamTeppati Losè, LorenzoHussain, GhulamTer Voert, EdwinHussain, NiamatTera, MelaniaHussain, SajjadTeramoto, AtsushiHussain, SuhailTeranishi, NobukazuHussain, TanveerTerreni, EleonoraHussain, ZahirTerroso, MiguelHussain, Zahir M.Tertiş, MihaelaHussein, HusamTeruel, Miguel A.Hussein, MoustafaTesar, JiriHuston, DryverTeslyuk, VasylHuszti, AndreaTessarolo, MartaHutchins, DavidTestoni, NicolaHutchinson, MichaelTetila, Everton CastelãoHuttunen, JanneTeunissen, PeterHuynh, NamTeymourian, HazhirHuynh, Thanh-CanhTfwala, SamkeleHuynh-The, ThienThammasan, NattapongHvattum, Lars MagnusThandavarayan, GokulnathHwang, Bor-JiunnThanh, Kien NguyenHwang, ChangmoThar, KyiHwang, Cheol-HongTharmarasa, RatnasinghamHwang, Chi-HungTheiler, JamesHwang, Dong-HwanThein, Chung KetHwang, Jae YounTheodosiou, AntreasHwang, Jung-hwanTheres Baby, TessyHwang, Keum-CheolThevenon, PaulHwang, Myun JoongThiamwong, LaddaHwang, Shun-FaThibault, GuillaumeHwang, Suk-WonThibbotuwawa, AmilaHwang, SunyouThiel, DavidHwang, WanSikThielemann, ChristianeHwang, YoungbaeThiery, YannickHwu, Jenn-GwoThireau, JérômeHyde, KayleighThives, LiseaneHyun, Byung GwanThiyagarajan, Jothi SaravananHyun, EuginThom, ChristianIacob, Ionut EmilThomas, ChristophIacono, MauroThomas, IliasIacovelli, ChiaraThomas, Jean-BaptisteIacoviello, FrancescoThomas, Philip A.Iadicicco, AgostinoThomas, StefanIanconescu, ReuvenThomaz, Carlos EduardoIancu, Bogdan (Technical University of Cluj-Napoca, Romania)Thompson, MichaelIancu, Bogdan (The Bucharest University of Economic Studies, Romania)Thompson, RodneyIano, YuzoThonfeld, FrankIban, Antolín LorenzanaThongprayoon, CharatIbarra Escamilla, BaldemarThubert, PascalIbarra-Castanedo, ClementeThuewissen, AlbertIbarra-Esquer, Jorge E.Thunemann, MartinIbarra-Manzano, Mario-AlbertoThurow, Brian S.Ibarra-Zarate, DavidThurow, KerstinIbl, MartinTian, DaxinIbrahim, Ahmed Mokhtar NagyTian, FengchunIchikawa, YoTian, Gui YunIckowicz, AdrienTian, JiandongIclodean, CalinTian, JiangweiIdier, JérômeTian, MiaoIeong, RicciTian, Qi-ChongIeracitano, CosimoTian, SaiqiIero, DemetrioTian, ShiyiIffelsberger, ChristianTian, TianIftikhar, NadeemTian, WeisongIgasaki, TomohikoTian, YanqingIgel, HeinerTian, YingweiIglesias Martínez, José AntonioTibaduiza Burgos, Diego AlexanderIglesias, Guillermo RTiberi, GianluigiIglesias, LuisTiboni, MonicaIgnaciuk, PrzemysŁawTiebe, CarloIgnasiak, Niklas KönigTiemann, JanisIgnatiev, VladimirTiemann, MichaelIgoe, DamienTiggelaar, Roald M.Igual, JorgeTigrini, AndreaIhm, InsungTiitta, MarkkuIida, DaisukeTijan, EdvardIida, MichihisaTimlin, Jerilyn A.Iijima, KazutoshiTimon, RabczukIio, TakamasaTimoney, JosephIitani, KentaTimopheev, AndreyIizuka, KotaroTin, PykeIjaz, Muhammad FazalTinarelli, RobertoIkeda, MakotoTinti, GemmaIkehashi, TamioTiong, Leslie Ching OwIkeno, ShinyaTirilly, PierreIkerionwu, CharlesTischler, DirkIkiades, ArisTiseo, CarloIkonomidou, Vasiliki N.Tithi, Jesmin JahanIkpehai, AugustineTittel, FrankIlev, IlkoTittmann, Bernhard RainerIlie, ConstantinTiwari, KshitijIliev, IvoTiwari, Kumar AnubhavIlieva, GalinaTiwari, PrashantIliyasu, Abdullah M.Tiwari, RajeshIllanes, AlfredoTiwari, Surya PrakashIllés, BalázsTjolleng, AmirIlmonen, PauliinaTkach, ItshakIlyas, AzharTlelo-Cuautle, EstebanIm, Tae HoTobe, YoshitoImakura, AkiraTobias, SilviaImamura, GakuToboła, DanielImber, JamesTobon, JorgeImburgia, AntoninoTodaro, Maria TeresaImmich, RogerTodeschini, SaraImming, PeterTodorov, YankoImoto, KeisukeTofael Ahamed, TofaelImperatore, PasqualeTofel, PavelImtiaz, MasudulToffanin, ChiaraImtiaz, Syed AnasTofil, ArkadiuszInan, Omer T.Tognetti, AlessandroInce, Ibrahim FurkanTogni, OlivierInga, EstebanTognolatti, PieroInglada, JordiToker, OnurInglese, GabrieleTokeshi, ManabuIngrid, TonhajzerováTokhi, OsmanIngwersen, JoachimTokuno, ShinichiInnocente, Mauro SebastiánTol, SerifeInnocenti, GiacomoTole, IldaIno, KosukeToledo, JavierInoue, AtsushiToley, Bhushan J.Inoue, JunichiTollefsen, DagInterdonato, RobertoTolvanen, JarkkoIntini, PaoloToma, Daniel MihaiInuso, GiuseppinaToma, KojiInzerillo, LauraToma, MilanInzunza-González, EverardoTomašegović, TamaraIoakimidis, ChristosTomasic, IvanIoan, TudosaTomasin, StefanoIoana, AdrianTomasz, RymarczykIoana, CornelTomaszewski, DariuszIoannidis, CharalabosTomažič, SimonIoannidis, KonstantinosTomczyk, ArkadiuszIodice, AntonioTomer, Vijay K.Ion, Rodica-MarianaTomita, SatoshiIonescu, Adrian M.Tomiyama, HiroyukiIonescu, Elena RodicaTomków, JacekIonescu, Radu TudorTomkowski, RobertIonescu, Tudor C.Tommaselli, Antonio M.G.Ionescu, Valeriu ManuelTomori, HirokiIonete, Eusebiu IlarianTonacci, AlessandroIordanescu, SergiuTonboe, RasmusIorga, MagdalenaTonello, Kelly CristinaIosa, MarcoTonello, SarahIqbal, Hafiz M. N.Tonetti, LorenzoIqbal, RazibTonezzer, MatteoIqbal, UmarTong, FengIqbal, ZafarTong, JinwuIriarte, XabierTong, KennethIrina, MocanuTong, LiangIrles, ClaudineTong, MingleiIrofti, PaulTong, Van-CanhIrrera, FernandaTong, WeitianIrvine, Gordon W.Tong, YexiangIsabelle, VerrierTong, ZhemingIsaeva, Vera I.Tönges, LarsIsakovic, Abdel F.Tonhajzerova, IngridIsam, ShahrourToniolo, RosannaIsar, AlexandruTopp, Elin AnnaIsgrò, FrancescoTorcal Milla, Francisco JoseIshiguro, YoshioTöreyin, HakanIshii, KeikoTorgaev, Stanislav N.Ishitani, LucilaTorgal, Fernando PachecoIslam, Hasan Mahmood AminulToride, KinyaIslam, Kh TohidulTorija Martinez, Antonio J.Islam, M. M. ManjurulTorikai, HiroyukiIslam, Md SaifulTornari, ViviIslam, Mohammad S.Torralba, AntonioIslam, MonsurTorre Toledano, DoroteoIslam, Muhammad AminulTorres, Javier MartínezIslam, NahinaTorres, Jhon James GranadaIslam, RobiTorres, José A.Islam, S. M. RiazulTorres, LizethIslam, SafulTorres, Pedro M. B.Islam, ShamaTorres, Ricardo Da SilvaIsoaho, NooraTorres-Cisneros, MiguelIsomura, TakuyaTorres-Diaz, IsaacIsoyama, NaoyaTorres-Freyermuth, AlecIsraeli-Korn, Simon DanielTorres-Gomez, IsmaelIssartel, JohannTorres-Martínez, Jose AlbertoIstrate, DanTorres-Ramírez, EduardoIstrate, DanielaTorres-Rivero, KarinaItrić, KatarinaTorres-Roman, Deni LibradoIudice, IvanTorres-Rua, AlfonsoIvan, LoredanaTorres-Sánchez, IreneIvanescu, MirceaTorres-Sospedra, JoaquínIvanjko, EdouardTorrisi, NunzioIvanov, DanilTorroba, TomasIvanov, IvanTorti, EmanueleIvanov, NikolayTortosa, Dídac D.Ivanov, Rosen StefanovTortschanoff, AndreasIvanov, ValentinTosi, DanieleIvanova, Alla V.Totero Gongora, Juan SebastianIvanovici, MihaiTóth, Ágota BányaiIvanovska, TatyanaToth, TeodorIvasic-Kos, MarinaToth, ViktorIvorra, EugenioToubanaki, DimitraIvsic, BranimirToubekis, ArgyrisIvšinović, JosipTouryan, JonathanIwai, DaisukeTown, ChristopherIwata, TatsuyaTowner, WalterIwendi, CelestineTowse, TheodoreIyota, TaketoshiToyama, NobuyukiIzadgoshasb, ImanToyama, ShigeruIzadyar, AnahitaToyoda, KentarohIzaguirre, AlbertoToyota, HirofumiIzaguirre, Alberto CordobaTraer, JamesIzal, MikelTrajkcovic, NebojsaIzawa, Kazuhiro P.Trajkovski, JovanIzonin, IvanTrakadas, PanagiotisIzquierdo-Fuente, AlbertoTramarin, FedericoIzu, NoriyaTrammell, Scott A.Izumi, YutaTran Anh, DuongIzydorczyk, JacekTran, Duc TanJaakkola, KaarleTran, Gia KhanhJabalameli, AmirhosseinTran, Kim PhucJabari, ShabnamTran, Thi HongJabba, DaladierTran, Thien ToanJaber, MonaTran, Van-ThaiJacinto, TiagoTran, YvonneJack, NaylonTranca, Dumitru CristianJackman, Joshua A.Traulsen, ImkeJackson, LisaTravagliati, MarcoJackson, NathanTravassos, BrunoJacob, Maik H.Travieso-González, CarlosJacob, RomainTravieso-Gonzalez, Carlos M.Jacobi, IanTravlos, IliasJacobsen, Rune HylsbergTregua, MarcoJacobson, Nicholas C.Trematerra, AmeliaJácome, CristinaTrendafilova, IrinaJacquir, SabirTreutler, KaiJacquot, MaximeTrevathan, JarrodJadidi, ZahraTrevisan, RodrigoJadwicienczak, WojciechTridello, AndreaJafari, AzadehTriggiani, Antonio IvanoJafari, MohammadTrigili, EmilioJafari, RahelehTrigona, CarloJafaritadi, MojtabaTrihinas, DemetrisJaganathan, ArunTrinh, Minh-ChienJagannath, JithinTrinh, ThuatJagiela, MariuszTrinh-Van, SonJagtap, SandeepTripathi, PraveenJahier Pagliari, DanieleTripathy, NirmalyaJaime, MarceloTripolitsiotis, AchilleasJain, AadharTripon-Canseliet, CharlotteJakab, GergelyTripp-Barba, CarolinaJakob, TorstenTriviño, AliciaJakub, LevTrögl, JosefJakubik, Wiesław Pawel Trogu, AntonioJakus, GregaTroiani, EnricoJalal, AhmadTrojaniello, DianaJalal, AhmedTronci, StefaniaJalali, FatemehTrontelj, JanezJalil, BushraTropea, MauroJaliu, CodrutaTroutman-Jordan, MeredithJallais, ChristopheTrovati, MarcelloJamalabadi, Mohammad Yaghoub AbdollahzadehTrovato, Maria RosaJames, RobinTrue Accord, Vladimir IglovikovJamil, GeorgeTrujillo, Logan T.Jamil, MohsinTrujillo-Romero, FelipeJamoos, AliTrujillo-Schiaffino, GerardoJämsä, TimoTrung, NgoThanhJamshidi, SajadTruong, NguyenJan, Sana UllahTruong-Hong, LinhJana, AtanuTrusiak, MaciejJana, Raghavendra B.Trusovas, RomualdasJanacova, DagmarTrusso Sfrazzetto, GiuseppeJanas, DawidTryggestad, JeanieJanczyk, GrzegorzTsagaris, ApostolosJandl, RobertTsai, Arthur Chih-HsinJaneliukstis, RimasTsai, Cheng-FaJanfaza, SajjadTsai, Chi-YiJang, Ho WonTsai, DavidJang, Hyong DooTsai, Hsiao-PingJang, HyungseokTsai, JamesJang, IngookTsai, JichiangJang, JinwooTsai, Kun-LinJang, JongwookTsai, Ming-FongJang, Seong HoTsai, Pang-WeiJang, Sung-HwanTsai, Tsung-HanJang, Sun-JooTsai, Yi-JuJang, Young-ChanTsai, Yu-TingJankau, JerzyTsai, Zuo-MinJankauskas, AudriusTsakalof, AndreasJanko, VitoTsakanikas, PanagiotisJankovic, MilicaTsamis, ChristosJankowski, ŁukaszTsang, Kim FungJankowski-Mihułowicz, PiotrTsangouri, EleniJannesari, ReyhanehTsangrassoulis, ArisJanos, RudolfTsarabaris, PanagiotisJanota, AlešTsaramirsis, GeorgiosJanowski, LukaszTsarev, AndreiJansen, Carl-PhilippTscheulin, ThomasJansen, Kaspar M.B.Tseng, Hsiao-TingJanusas, GiedriusTseng, Shu-MingJara, NicolasTseng, Tsun-MingJaradat, AbdullahTseng, Victor Farm-GuooJaradat, Mohammad A.Tseng, Wei-LungJaramillo-Morán, Miguel A.Tseng, Yi-HsingJarczewska, MartaTseng, Yuan-WeiJaren, CarmenTsenova, BoryanaJarolmasjed, SanazTsiakas, KonstantinosJaroslav, VrchotaTsigaridas, GeorgiosJaroszewicz, ZbigniewTsigaridas, Georgios N.Jarzabek, DariuszTsigopoulos, Andreas D.Jasielec, Jerzy J.Tsiligiridis, Theodore A.Jasińska, ElżbietaTsiligkaridis, John (Ioannis)Jasinski, GrzegorzTsipouras, Markos G.Jasinski, RadoslawTsiropoulou, Eirini EleniJastrzębska, MałgorzataTsogka, ChrysoulaJat, PrahladTsolakis, Apostolos V.Jatužis, DaliusTsoukalas, DimitrisJauregui, Juan CarlosTsoumanis, GeorgiosJáuregui-Vázquez, DanielTsounidi, DimitraJavadi, BahmanTsow, Francis (Tsing)Javadi, HamedTsubasa, MinematsuJavornik, TomazTsuboi, TsutomuJavorski Eckert, JonyTsuburaya, TomonoriJawinski, PhilippeTsuda, ShuichiJaworek-Korjakowska, JoannaTsui, Brian Y.Jaworski, PiotrTsuji, SatoshiJayachandran, K. P.Tsujii, ToshiakiJayakody, NimeshTsukahara, TsuneoJayaraman, ChandrasekaranTsuru, Takeshi GoJayarathna, TitusTsutsui, MakusuJayathunga, SadeepaTsutsumida, NarumasaJayousi, SaraTu, ChengJayson, GiffordTu, LiangchengJech, J. MichaelTu, Min-chengJechow, AndreasTu, RuiJee, SolkeunTu, ShanshanJee-In, KimTu, YifengJeerapan, ItthiponTu, ZhigangJegelevičius, DariusTucan, PaulJekova, IrenaTucciarelli, TullioJelitto, JensTuci, ElioJelonnek, JohnTucu, DumitruJeltsch, AlbertTudorache, FlorinJen Hong, TanTufvesson, FredrikJenčová, EdinaTuhtan, Jeffrey A.Jendernalik, WaldemarTukhbatullin, AdisJendli, ZouhaierTulbure, AdrianJeng, YihTuluri, FrancisJenkins, BrianTumakov, DmitriiJenkins, ChadTung, Tao-HsinJenkins, KennethTung, Tran ThanhJenkins, Samir V.Turalchuk, PavelJennifer A., SchulienTuran, CigdemJeon, ByunghwanTurco, SimonaJeon, Heung SeokTurcza, PawelJeon, Jun-CheolTurgul, VolkanJeon, Ju-WonTurner, Martin J.Jeon, MansikTusset, Angelo MarceloJeon, SangminTutak, MagdalenaJeong, Chang KyuTwu, Ruey-ChingJeong, DongwonTylecek, RadimJeong, Han-YouTyler, DavidJeong, Hoon EuiTyliszczak, BożenaJeong, In CheolTypiak, AndrzejJeong, Jin SuTyralis, HristosJeong, Jin-WooTysiąc, PawełJeong, JungsikTzallas, AlexandrosJeong, JunhoTzeng, Huey-MingJeong, Seong HoonTzounis, LazarosJeong, Young-SeobTzouvaras, MariosJerez-Mesa, RamónTzu Wu, YuJerkovic Stil, VedranaUbe, TakujiJerković Štil, VedranaUbertini, FilippoJesenik, MarkoUccheddu, FrancescaJessa, MieczyslawUchibe, EijiJessel, Jean-PierreUchiyama, KenjiJessel, JoshUddin Ahamed, NizamJesus, IsabelUddin, JiaJewell, JosephUddin, Mohammad SalahJezeršek, MatijaUdelhoven, ThomasJezova, JanaUdris, DainiusJhon, Young MinUdroiu, RazvanJi, BaofengUdugama, AsangaJi, JianUeberschär, OlafJi, KefengUeda, TaroJi, ZhanlinUgarte, Juan P.Ji, ZhengUgbolue, UkadikeJia, ChunrongUgo, PaoloJia, DongbaoUgon, AdrienJia, FucangUherek, FrantišekJia, KunUhl, AndreasJia, PinggangUhlemann, SebastianJia, ShaofengUjihara, MasakiJia, WenyanUl-Abdin, ZainJia, XiaoqingUlaby, FawwazJia, XiaotingUlacha, GrzegorzJia, XiaoweiUlapane, NalikaJia, XudongUlbrich, DariuszJia, YuUllah, AminJia, YunfangUllah, Amm SharifJia, ZhifengUllah, IsrarJian, Bo-LinUllah, KifayatJian, MuweiUllah, MohibJiang, ChanghuiUllo, SilviaJiang, ChaoUllo, Silvia LiberataJiang, ChaoyangUllrich, TorstenJiang, ChaozheUlrich, MarkusJiang, ChengminUlusoy, BeyzaJiang, ChunxiaoUm, DuganJiang, DaweiUm, JumyungJiang, DazhiUmberto, RiccardiJiang, DongUngurean, IoanJiang, FanUngureanu, FlorinaJiang, HaoUnnithan, Ranjith RajasekharanJiang, HongchuanUno, TomohideJiang, HongjieUnterthiner, ThomasJiang, HuaxingUr Rasool, RaihanJiang, JiahuanUrata, JunjiJiang, JiajiaUrbanek, JacekJiang, JingUrniezius, RenaldasJiang, JunUrquiza-Aguiar, Luis F.Jiang, JunfengUrsu, IoanJiang, JunjunUrwyler, PrabithaJiang, JunleUsama, Syed MuhammadJiang, JunzhengUsarek, ZbigniewJiang, MiUseche, Sergio A.Jiang, MingshunUshakov, NikolaiJiang, MingzheUshakov, Nikolay M.Jiang, NiUstinovičius, LeonasJiang, PingUstuner, MustafaJiang, QiUsui, NoriyoshiJiang, QingshanUtkin, AndreiJiang, RuinianUtrilla-Manso, ManuelJiang, ShanUtsuzawa, ShinJiang, ShuqiangUykur, EceJiang, TingyiUzhga-Rebrov, OlegJiang, WanshouVacavant, AntoineJiang, WeikangVaccaro, LuisJiang, WenchaoVafaee, FatemehJiang, WuguiVagedes, JanJiang, XiVahabi, MaryamJiang, XiantaVahtmäe, EleJiang, XiqianVaiana, NicolòJiang, XuefengVaiana, RosolinoJiang, YinlaiVaida, CalinJiang, YizhangVakanski, AleksandarJiang, ZhiyuVakhania, NodariJiang, ZhuangDeVakros, JohnJianjiang, WangValais, IoannisJiao, ChaoqunValais, Ioannis G.Jiao, HeidiValandro, SilvanoJiao, PengchengValčić, MarkoJiao, WanguoValderrama, CarlosJiao, XiaohongValderrama, JoaquinJiao, YuboValdettaro, LorenzoJie, Zhanming (Allan)Valdez, Manuel TravassosJigena-Antelo, BismarckValdivia-Moral, PedroJimenez Angeles, LuisValença, JónatasJiménez Ballesta, RaimundoValencia-Palomo, GuillermoJiménez Martín, Juan JoséValente, AntonioJiménez Millán, JuanValente, Renata Kelly MendesJiménez Ramos, Maria Del CarmenValentin, JanJiménez, Alfredo ArcosValentini, ValentinoJimenez, Antonio R.Valentino, RobertoJiménez, FelipeValenzuela, Jose LuisJimenez, MelanieValera, EnriqueJimenez, RicardoValeria, OsvaldoJiménez-Alonso, Javier FernandoValeriani, DavideJiménez-Herranz, José MiguelValero, EdelmiraJimeno-Morenilla, AntonioValero, MariaJin, BoValero, Mario M.Jin, ChaowuValiente, DavidJin, CongruiValigi, Maria CristinaJin, GeValkai, SándorJin, GuofanVallan, AlbertoJin, HaimingVallejos, SaúlJin, HanbitVallet, BrunoJin, HaoVallicelli, Elia ArturoJin, HuaipingValluzzi, Maria RosaJin, JingValous, NektariosJin, LishengValsesia, DiegoJin, Ning-deValt, MatteoJin, Ren ChengValtierra-Rodriguez, MartinJin, Seung-SeopValušis, GintarasJin, ShuanggenValverde, AlfredoJin, TianValverde, Jose R.Jin, XiValvo, Paolo SebastianoJin, Xinvan der Ham, InekeJin, Xue-boVan Der Perre, LiesbetJin, YuanweiVan Der Velde, EnnoJin, ZhigangVan Der Werff, Harald M.A.Jin, ZongwenVan Der Zwaard, StephanJino, RamsonVan Do, TienJiralerspong, TrongmunVan Eerdenburg, FrankJo, DongsikVan Erps, JurgenJo, Han-ShinVan Erp-van Der Kooij, LennyJobst, MarkusVan Gasse, KasperJochem, Warren C.Van Gastel, MarkJócsák, IldikóVan Hulle, MarcJoe, HangilVan Le, DucJofre Roca, LluísVan Schaik, JaapJohannes, Hans-HermannVan Staden, RalucaJohannes, KieferVan Timmeren, JanitaJohansson, ChristerVan Torre, PatrickJohns, EdwardVan Vaerenbergh, StevenJohnson, AnjuVan Verre, WouterJohnson, EricVan Winden, StevenJohnson, Lee F.Van, Nguyen Thi PhuocJohnson, MarkVandemeulebrouck, JeanJohnson, OwenVandewalle, PatrickJohnston, J. SpencerVangelista, LorenzoJohnstone, WalterVanlanduit, SteveJokinen, OlliVannozzi, GiuseppeJolly, PawanVantroys, ThomasJoly, CyrilVaradarajan, VijayakumarJombo, GbanaibolouVaranini, MaurizioJonas, StephanVaranis, MarcusJonas, Stephan M.Vardaki, MarthaJones, Albert T.Vardasca, RicardoJones, Rodney P.Varga, PalJones, SamVargas, JavierJönsson-Niedziolka, MartinVargas-Rodriguez, EverardoJonuscheit, JoachimVargic, RadoslavJoo, Ki-NamVaro-Martinez, MartaJoonas, IivanainenVaron Perez, CarolinaJoos, VictorVarotsos, CostasJordan, AlastairVarotsos, George K.Jordans, RoelVarriale, AntonioJörg, BeckmannVarum, TiagoJorge Luis Zambrano, MartinezVaseashta, AshokJose, MarcoVashist, Sandeep KumarJosep, ArnalVasilakos, AthanasiosJoseph, Francis KalloorVasilakos, ChristosJoseph, JamesVasilateanu, AndreiJoseph, RoshanVasile, Bogdan StefanJoshi, Gyanendra P.Vasile, SimonaJoshi, Gyanendra PrasadVasilescu, AlinaJoshi, NiravVasilescu, Mircea DorinJoshi, SaumyaVasiliev, AlexeyJoubert, TrudiVasiljević, GoranJouda, MazinVasiljević-Radović, DanaJoung, JingonVasquez, Jorge A. TobonJovanović, VukicaVásquez-Correa, Juan CamiloJovanovski, VaskoVasylyev, DmytroJove, EstebanVatalaro, FrancescoJovičić, NenadVathy-Fogarassy, ÁgnesJoyce, MalcolmVaughan, Christopher L.Jozanović (Horvat), MarijaVaughn, Israel J.Józwik, MichałVauhkonen, MarkoJraidi, ImèneVavrinský, ErikJu, FengVaz Alves, GleiferJu, HeongkyuVaz, CarlosJu, Shen-hawVaz, Pedro D.Ju, XiangyangVaz, WarrenJuan, Carlos G.Vázquez, Carlos Andres LunaJuang, Jyh-ChingVazquez, FranciscoJuárez-Gracia, Antonio-GustavoVazquez-Becerra, Guadalupe EstebanJudd, MartinVázquez-Castillo, JavierJudek, SlawomirVazquez-Castro, ÁngelesJudit, OláhVazquez-Guardado, AbrahamJuez, CarmeloVecellio, EliaJugessur, AjuVeeran Ponnuvelu, DineshJugo, JosuVeeraraghavan, PrakashJuhar, JozefVega, ManuelJuhasz, JanosVega, MiguelJulio, Bastos ArrietaVega-Oliveros, Didier AugustoJulrat, SakolVegas Hernández, Jesús M.Jun, Dong SanVégh, Attila GergelyJun, KondohVehkaoja, AnttiJun, QiuVeitch-Michaelis, JoshJun, Sang BeomVela, NuriaJun, YoungchulVelas, MartinJung, Chien-ChengVelasco, Juan RamónJung, DaewoongVelasco-Alvarez, FranciscoJung, DongwonVelázquez, RamiroJung, HaejoonVelebil, LukášJung, Hee-TaeVelev, Dimiter GeorgievJung, Ho-YoulVélez, José FranciscoJung, Jason J.Velha, PhilippeJung, JiyoungVelichko, AlexanderJung, Kwang-HyoVella, FilippoJung, Seong-OokVelmuzhov, AlexanderJung, Soon KiVeloso, MarcoJung, SunghunVelotta, RaffaeleJung, Woo-SungVelten, HermanoJung, Yong JuVelthuis, JaapJung, YoonsungVeltink, PeterJung, YunhoVelusamy, GandhimathiJungeblut, ThorstenVėlyvytė, SeverinaJunghans, MarekVenafra, SaraJunior, AntonioVenanzoni, GiuseppeJunli, LiVendik, Irina B.Junyent-Ferré, AdriàVenditti, IoleJuodkazis, SauliusVendittozzi, CristianJuraic, KrunoslavVenediktov, VladimirJurek, MichałVenegas, PabloJūrėnas, VytautasVenkataraman, RajeshJurić-Kaćunić, DanijelaVenkatasubramanian, AnandramJuřík, VojtěchVenkatasubramanian, KrishnaJurišić, MladenVenkatraman, SitalakshmiJusas, VaciusVentouras, Erricos-ChaimJusko, OttoVentura, Joao OliveiraKaajakari, VilleVentura, Silvia MastrolemboKabakchiev, ChristoVenturino, LucaKabala, CezaryVenu, AnnamdasKabassi, KaterinaVerbelen, TimKabir, K. M. MohibulVerbiest, JorisKabiri Ameri, ShidehVerbree, EdwardKaboli, MohsenVerde, FrancescoKacem, NajibVerde, LauraKacem, ThabetVergara, DiegoKachanov, Vladimir KlimentievichVergaz, RicardoKacik, DanielVergiev, StoyanKačmařík, MichalVergos, GeorgeKačur, JánVerhaert, NicolasKaczmarczyk, JarosławVerhulst, TobiasKaczmarek, Adam L.Veríssimo, MartaKaczmarek, KrzysztofVerkholyak, OxanaKaczmarek, MariuszVerlekar, TanmayKaczmarek, MichalVerma, Ajay K.Kaczmarek, PiotrVerma, ManishaKadam, AvinashVerma, RajkumarKaddoum, GeorgesVerma, SumitKadhum, Mohammad R.Verna, AlessioKaewunruen, SakdiratVerona, EnricoKagawa, KeiichiroVeronika, SzücsKager, JulianVerotti, MatteoKagiyama, NobuyukiVershovskii, AntonKahng, SungtekVerstaevel, NicolasKainz, AndreasVerstraete, David BenjaminKaiser, SusannaVertuccio, LuigiKaiser, Thomas M.Verwilst, PeterKaiwartya, OmprakashVescio, BasilioKajánek, PavolVesna, ZeljkovicKajimoto, ShinjiVessio, GennaroKajiwara, YusukeVestergaard, Mundelanji C.Kakarountas, AthanasiosVéstias, MárioKaklauskas, ArturasVetter, ChristianKalalas, CharalamposVettoretti, MartinaKalambura, SanjaVetturi, DavidKalangala Sikkanther Batcha, Syed AliVetyukov, YuryKalashnikov, AlexanderVezzetti, EnricoKalatzis, NikosVia, BrianKalina, JanVialetto, GiulioKalinicheva, EkaterinaViassone, MilenaKaliniewicz, ZdzisławVibell, JonasKalinnikov, VladislavVicarelli, LeonardoKaliske, MichaelVicencio, Rodrigo BarrazaKaliszewski, MironVicent, José F.Kalita, Jakub ZbigniewVicente Pérez, RubénKalivas, GrigoriosVicente, Ferreira De Lucena JuniorKaliviotis, StathisVicente, María AsunciónKalkanis, KonstantinosVidal, JoelKalkman, JeroenVidal, Jorge MaestreKalkowski, Michał K.Vidal-Verdú, FernandoKalkur, T.S.Vidic, JasminaKalliatakis, GrigoriosViegas, VítorKalliokoski, MattiVieira Filho, JozueKallioniemi, ElisaVieira, Alex BorgesKallmayer, ChristineVieira, DarioKalluru, PolirajuVien, Benjamin StevenKalman, HaimVien, Quoc-TuanKalogeras, DimitrisVieri, MarcoKalogeropoulos, KleomenisViespe, CristianKalogianni, DespinaViggiano, AndreaKaloop, MosbehVigneron, VincentKaloxylos, AlexandrosVignoli, ValerioKaltiokallio, OssiVignudelli, StefanoKaltsas, GrigorisViik, JariKalyana Sundaram, Ramalingam VenkatVila Sobrino, Xosé AntónKam, MosheVilajosana, XaviKamanina, NataliaVilanova, XavierKamavuako, ErnestVilaseca Ricart, MeritxellKambezidis, Harry D.Vilhelm, JanKameda, MasaharuVilla, AndreaKamel, Mohamed A.Villa, CarlaKamenar, ErvinVilla, IreneKamencay, PatrikVilladangos, JesusKa-Meng, LeiVilladangos, José ManuelKamikawachi, RicardoVillafaina, SantosKamini, YadavVillalba, JavierKamińska, DorotaVillalonga, AlbertoKaminska-Chuchmala, AnnaVillalon-Turrubiates, Ivan E.Kamiya, OsamuVillamizar, Sandra R.Kammer, DietrichVillani, Maria GabriellaKamola, MariuszVillanueva Vásquez, DanielKampa, AdrianVillanueva-Llauradó, PaulaKamshilin, AlexeiVillar, José R.Kan, ChiwaiVillarejo Mayor, John JairoKanakaris, VenetisVillarreal-Gómez, Luis JesúsKanamoto, ToshikiVillaverde, Miguel Ángel NayaKanarachos, StratisVillecco, FrancescoKandris, DionisisVilleneuve-Faure, ChristinaKanellopoulos, DimitrisVillordo Jimenez, IcliaKanerva, MikkoViman, LiviuKang, BongsoonVimarlund, VivianKang, ByeongkeunVimieiro, Claysson BrunoKang, ByungseokVinayagam, ArangarajanKang, ChulhunVinayaraj, PoliyapramKang, FeiVincent, TimothyKang, GuVincenti, Maria AuroraKang, Gu EonVincenzi, LorisKang, HyunchulVinel, AlexanderKang, InpilVingerhoeds, Rob A.Kang, JamesVinh, Nguyen QuangKang, JeeunVinogradov, AndreyKang, Je-WonVinogradov, Evgenii (Genia)Kang, JianVinogradov, SergeyKang, KevinVinogradov, Sergey L.Kang, LeiVinueza Naranjo, Paola GabrielaKang, Li-WeiVipulanandan, CumaraswamyKang, Min-SeokVirag, ZoltanKang, MoonsooVirdee, BalKang, SaewonVirdis, AntonioKang, Suk JuVirgala, IvanKang, WoonhakVirk, SimerjeetKang, Yang JunVirkki, JohannaKang, YuejunVirmontois, CédricKang, ZihoVirzonis, DariusKangro, KerstiVisconti, PaoloKaniewski, PawełVisentin, FrancescoKano, ShinyaVisvizi, AnnaKanrzavelou, IoannaVitale, MarcelloKansanen, KimmoVitaletti, AndreaKant, KrishnaVitali, RachelKantarci, BurakVitek, StanislavKantartzis, Nikolaos V.Viti, CarloKanzian, ChristianVittadello, MicheleKao, Hsuan-LingVittoria, BruniKao, Yung-ChouVitulano, DomenicoKapcia, Konrad JerzyVivanco, Alfonso PadillaKapczyński, AdrianVivar-Quintana, Ana M.Kapitsaki, GeorgiaVivas, Oscar AndresKaplan, AndreyVivek, SkandaKapłonek, WojciechVivet, DamienKaplun, DmitriiVižintin, GoranKapoulas, VaggelisVizza, PasqualeKappatos, VassiliosVizzarri, AlessandroKapuscinski, TomaszVizziello, AnnaKara De Maeijer, PatriciaVlachogiannis, DiamandoKarabanovas, VitalijusVladareanu, LuigeKaragulian, FedericoVlădăreanu, VictorKaraim, MalekVlad-Bubulac, TachitaKarakašić, MirkoVladescu, MarianKarakatič, SašoVladimir, BadenkoKarakostas, AnastasiosVladislavić, NivesKaralitsios, SpyrosVladyko, AndreiKaramacoska, DianaVlaj, DamjanKaramitsos, IoannisVlase, SorinKarantanellis, EfstratiosVo, Ba-NguKarapetis, StefanosVo, Nguyen-SonKaratza, AgelikiVochin, MariusKarg, TimoVodeneev, VladimirKargas, GeorgeVodnar, Dan C.Kargas, GeorgiosVoglhuber, ThomasKargoll, BorisVoglhuber-Brunnmaier, ThomasKarim, AhmedVoglitsis, DionisiosKarim, AsifVohnsen, BrianKarim, LutfulVoiculescu, IoanaKarim, NazmulVojtech, LukasKarim, NissarVolckaert, BrunoKarim, ZehaniVoliani, ValerioKarimi, Hamid RezaVolk, JánosKarimi, HassanVolker, KörstgensKarimian, NimaVolker, SchatzKaripott, SalilVolkov, VadimKarkanis, StavrosVolkova, Elena G.Karkazis, Panagiotis A.Vollero, LucaKarki, SitaVollrath, AndreasKarkula, MarekVolmajer, JulijaKarlovsky, PavelVolmer, MariusKärnfelt, CamillaVolná, EvaKarnowski, KarolVolosencu, ConstantinKarnowski, Thomas P.Volpi, AndreaKaroly, Philippa JaneVoltr, PetrKaroń, GrzegorzVon Holst, ChristophKarpetis, AdoniosVon Toussaint, UdoKarpov, DmitryVon Wangenheim, AldoKarsai, ArpadVornicu, IonKarsznia, IzabelaVosselman, GeorgeKarsznia, Krzysztof R.Voulkidis, ArtemisKarthik, RajVoulodimos, AthanasiosKartsakli, ElliVoutetaki, Maria–StylianiKaruppannan, Senthil KumarVouyioukas, Demosthenes D.Karuppuswami, SaranrajVozel, BenoitKarvonen, HeikkiVoznak, MiroslavKarvounis, ArtemiosVražić, MarioKarwowski, KrzysztofVrba, JanKaryakin, ArkadyVrbka, JaromírKaryotis, VasileiosVretos, NicholasKasai, NaoyaVrhel, MichaelKašalynas, IrmantasVrigneau, BaptisteKashcheyev, AntonVrochidis, StefanosKashevnik, AlexeyVroegindeweij, BastiaanKashif, MuhammadVrysis, LazarosKasiński, KrzysztofVu, Huong Thi ThuKasneci, EnkelejdaVu, Ngoc SonKasnesis, PanagiotisVu, Viet-HungKasprzak, WłodzimierzVucinic, DeanKasprzyk, JerzyVuillaume, Jean-FrancoisKassanos, PanagiotisVujović, IgorKassaras, IoannisVukovic, MarinKasser, MichelVukovic, NatashaKastis, GeorgeVulic, MilivojKästner, ChristianVulpe, AlexandruKästner, MarkusVulpe, AlexanduKastridis, AristeidisVun Jack, ChinKataev, EvgenyVural, MertKataoka, HirokatsuVyšata, OldřichKateris, DimitriosVysocký, AlešKaterski, AtanasWachulak, PrzemysławKatina, PolinpapilinhoWacogne, BrunoKatinić, MarkoWada, TomohitoKatkovnik, VladimirWada, YujiKato, KunihiroWade, MatthewKato, MasayukiWagdy, AymanKatona, JozsefWagih, MahmoudKatos, VasilisWagner, ChristianKatsikas, SokratisWagner, NealKatsikis, GeorgiosWagner, PatrickKatsikouli, PanagiotaWagner, Patrick H.Katsumata, KeiWagner, Stefan RahrKatt, BaselWagner, StevenKatunin, AndrzejWahaia, FaustinoKatz, EvgenyWahl, MartineKauhaniemi, KimmoWahlström, JohanKaur, AmanWajman, RadosławKaur, AmardeepWajrak, MagdalenaKaur, HarleenWakamiya, NaokiKaur, NavpreetWakayama, ToshitakaKaushal, VinayakWalas, KrzysztofKaushik, AbhishekWalcarius, AlainKaushik, AniruddhaWalcek, ChrisKavadias, KosmasWalch, OliviaKavallieratou, ErginaWalczak, KrzysztofKavianpour, SanazWalczak, NataliaKawabata, NorifumiWalczak, StanisławKawakami, JunWalczykowski, PiotrKawakami, MichiyukiWaldman, ChristophKawakita, HidetakaWales, DominicKawala-Sterniuk, AleksandraWales, Dominic J.Kawalec, AdamWałęsa-Chorab, MonikaKawalec, JacekWalker, JacquelineKayacan, ErdalWalker, Ross M.Kayes, A. S. M.Wallace, Carlington W.Kazak, JanWallace, Gregory Q.Kazanavičius, EgidijusWaller, MichaelKazancı, ZaferWallner, MarioKazanskiy, NikolayWalsh, KerryKazanzides, PeterWalters, John Paul N.Kazimierczuk, KrzysztofWalther, AndreiKazimierski, WitoldWalton, JeffreyKaźmierczak, RafałWan, LeiKazuya, MoriWan, LiliKazys, RymantasWan, ShaohuaKe, ChihKunWan, ShibiaoKe, XiaoWan, WenhuiKe, YanWan, XiaoyuKearney, RobertWandowski, TomaszKearns, CathrionaWang, AichenKechagias-Stamatis, OdysseasWang, AiningKefauver, Shawn CarlisleWang, BaicunKehl, ChristianWang, BaoshuaiKehl, FlorianWang, BinKeillor, JeffreyWang, BingKeinänen, MarkkuWang, BoKeiser, GeorgeWang, CemingKelemen, MichalWang, ChangchengKeler, AndreasWang, ChanghongKeller, StephanWang, Chao (Singapore)Keller, YosiWang, Chao (Tianjin University, China)Kelley, StevenWang, Chao (University of Chinese Academy of Sciences, China)Kelner, JanWang, Chao (University of Science and Technology of China, China)Kemper, BjörnWang, Chao (USA)Kemsaram, NarsimluWang, Chao (Yunnan University, China)Kendler, ShaiWang, ChenKenett, Ron S.Wang, ChengKengelbach-Weigand, AnnikaWang, Cheng-LiangKenry, KenryWang, ChenxuKent, AnthonyWang, Chih-HungKenyeres, MartinWang, Chi-KueiKenyon, Nicholas J.Wang, ChuanlongKeogh, John G.Wang, DaleiKeramaris, EvangelosWang, DaweiKeramidas, GeorgiosWang, DepengKerekes, JohnWang, Di (Germany)Kerekes, TamásWang, Di (Singapore)Kereszturi, AkosWang, DingKerman, KaganWang, DingkangKermarrec, GaëlWang, DongKern, StefanWang, DongyiKerr, DonaldWang, Edward J.Kertész, GáborWang, Fa-QiangKesavan, ArunWang, Fei (Nanjing University of Aeronautics and Astronautics, China)Keser, TomislavWang, Fei (Northeastern University, China)Keshmiri, SoheilWang, Fei (Southern University of Science and Technology, China)Keszöcze, OliverWang, Fei (Tsinghua University, China)Kevat, AjayWang, Fei (Xi’an Jiaotong University, China)Khadka, RoshanWang, FeipengKhalaf, WalaaWang, Fu-KangKhalid, AzfarWang, FupengKhalife, JoeWang, FuyongKhalil, IslamWang, GuanghuiKhalili Mobarakeh, AliWang, GuifenKhalili, AshkanWang, GuijinKhalili, SiavashWang, GuoliKhameneifar, FarbodWang, GuomingKhamesi, Atieh R.Wang, HaiboKhamis, HebaWang, HairongKhamis, MohamedWang, Hao (China)Khan, AsiyaWang, Hao (USA)Khan, AzizWang, Harry HaoxiangKhan, FaheemWang, HeKhan, Foysal ZahidWang, HengKhan, IftekharWang, He-shegKhan, Mohammad A.Wang, HongKhan, MuhammadWang, HongboKhan, Muhammad AhmedWang, HongwuKhan, Muhammad ShujaWang, HongyanKhan, MuradWang, HongyuKhan, NabeelWang, HongzeKhan, NaimulWang, HongzhiKhan, PervezWang, Hsiang-ChenKhan, SulemanWang, Hsueh-Cheng NickKhan, TareqWang, HuKhan, UsmanWang, HuabingKhan, WasiqWang, HuaqingKhan, Zeeshan AhmadWang, HuaxiangKhanal, Salik RamWang, HuxuanKhanesari, Mojtaba AhmadiehWang, JiabinKhanh Le, Nguyen QuocWang, JiachouKhanna, SouravWang, Jia-JungKharbach, MouradWang, JiananKharel, RupakWang, JiangtaoKharismalatri, Hefryan SukmaWang, JianqiKhatib, Emil J.Wang, JiaweiKhatibi, Akbar A.Wang, JiazeKhatibi, SiamakWang, Jin (Fujian University of Technology, China)Khatir, SamirWang, Jin (Yangzhou University, China)Khatoon, AsmaWang, Jing (China)Khattab, TawfikWang, Jing (UK)Khattak, Asad MWang, JingchenKhawam, KindaWang, JinghuiKhazraee, MiladWang, JingjingKhelifi, LazharWang, JingyuKhemchandani, Sunil L.Wang, JingzheKhemmar, RedouaneWang, JinshengKhenchaf, AliWang, Jin-YuanKhilkevich, VictorWang, JuKhilwani, RakeshWang, JunKhonina, Svetlana N.Wang, JunfengKhoramshahi, EhsanWang, JunqiKhorov, EvgenyWang, JunshengKhoshnam, MahtaWang, Kai (China)Khosla, AjitWang, Kai (USA)Khosla, Kiran E.Wang, KainanKhosravy, MahdiWang, KanKhoury, HiamWang, Ke (Beijing University of Posts and Telecommunication, China)Khushi, MatloobWang, Ke (Harbin Institute of Technology, China)Kia, Seyed MostafaWang, KeshengKiaghadi, AminWang, Kevin I-KaiKiam, Jane JeanWang, KeyanKiang, Jean-FuWang, Kun (China)Kiefer, RudolfWang, Kun (USA)Kierat, WojciechWang, Kun ChingKiesewetter, DmitryWang, KunchingKik, TomaszWang, LefanKikuchi, HiroakiWang, LeiKildashti, KamyarWang, LiangKilikevičius, ArtūrasWang, Liang-HungKillgore, JasonWang, LiangminKim, BeomSeokWang, LidaiKim, BohungWang, LifengKim, Byeong HeeWang, LiLiKim, Byung HoonWang, Lin (China)Kim, Byung-CheolWang, Lin (Korea)Kim, Byung-GyuWang, LingKim, Chang-SeiWang, LingyunKim, ChangUWang, LinweiKim, ChangwonWang, LiuKim, ChulhongWang, LizhenKim, DaeEunWang, Long (China)Kim, DaeheeWang, Long (USA)Kim, Daniel Eun-SeongWang, LujiaKim, Dong KeunWang, LuyuKim, Dong MinWang, MengKim, Dong-SeongWang, MiKim, Dong-ShikWang, MingkangKim, Duk KyungWang, Ming-ShyanKim, DukhyeonWang, MinjuanKim, Gi-WooWang, Mu-chunKim, GunzungWang, MuguangKim, HakjinWang, NanKim, Hak-juWang, NingKim, HaroldWang, PeihongKim, Hoe JoonWang, Peng (China)Kim, Ho-YeonWang, Peng (UK)Kim, Hye-JinWang, Pi-ChungKim, HyeokWang, PingKim, Hyeon TaeWang, QianKim, Hyoung-HoWang, QianchengKim, Hyug-HanWang, QiangKim, Hyun JaeWang, QingQingKim, Hyun K.Wang, Qiong-HuaKim, HyunbumWang, QiuKim, Hyung SeokWang, QiuguKim, In YoungWang, QiuhuaKim, InsooWang, RenjieKim, JaeyoungWang, RuiKim, Jang AhWang, RuijunKim, Jea SooWang, SaiKim, JeehyeongWang, SenKim, Jee-InWang, SenzhangKim, JeonggukWang, ShapengKim, JeonghyunWang, ShengkaiKim, Jeong-JungWang, ShengnianKim, JeongraeWang, ShengqiangKim, JibumWang, Sheng-ShihKim, Ji-HwanWang, Shin WenKim, JihyeWang, ShoukunKim, Jin YoungWang, ShuangquanKim, JingyunWang, Shu-CingKim, JinkiWang, ShuohongKim, JinwookWang, TaoKim, Jin-YeonWang, TaoyangKim, JinyoungWang, TianKim, Jong-ChanWang, TianyiKim, Jong-DeokWang, WeiKim, Jong-EunWang, WeisongKim, Jong-HeonWang, WeizhenKim, JonghoekWang, WenKim, JoonyoungWang, WenguanKim, Jung Tae (Steve)Wang, WenguangKim, JunghoonWang, WenjinKim, JunghwanWang, WenshuoKim, Jung-hyunWang, WilliamKim, JungkwunWang, XanderKim, JungyoonWang, XianboKim, JusungWang, XianghongKim, Kang WookWang, XianpengKim, KeonwookWang, XianzhiKim, Ki-IlWang, XiaochanKim, KimanWang, XiaofengKim, KiyoungWang, XiaoyuKim, Kwang-YulWang, XiaoyuanKim, Kyeong-seopWang, XilinKim, KyujungWang, XiluanKim, KyungsooWang, XingyuanKim, Mi JeongWang, XinhengKim, MinWang, XinyuKim, Min JungWang, XinZhenKim, MinsuWang, XiuminKim, Moon DeockWang, XuanKim, Moon IlWang, XuefengKim, MoonseongWang, XuewenKim, MyungsunWang, XuezhiKim, NamWang, YanKim, Nam-YoungWang, YangKim, Pyung-SooWang, YanyanKim, RobinWang, YawenKim, SanghyunWang, YeliKim, SangkilWang, YiKim, SangtaeWang, Yibing M.Kim, Seok-KyoonWang, YideKim, Seong JinWang, YingKim, Seong-CheolWang, YingyingKim, Seong-EunWang, YipingKim, Seung-SepWang, YishouKim, ShihoWang, YongKim, SoaramWang, YouqingKim, Soo YounWang, YuKim, Soo YoungWang, Yu-ChengKim, SooyoungWang, YueKim, SunghoWang, YufengKim, SungjunWang, YuhaoKim, SunwookWang, YuhuiKim, TaehongWang, YushengKim, TaeyeonWang, YuzheKim, WonjunWang, ZhanyongKim, Won-TaeWang, ZhaoKim, YejinWang, ZhaobinKim, YongWang, ZheKim, Yong SikWang, ZhenboKim, Yong-GukWang, ZhengKim, Yong-HwaWang, ZhengfangKim, Yong-JooWang, ZhengqiangKim, YongminWang, ZhengyuKim, Yoon-ChulWang, ZhenxinKim, YoungbokWang, ZhenyuKim, Young-DukWang, ZhenzhenKim, Young-GabWang, ZhenzhouKim, YounghoWang, Zhi-BoKim, YoungokWang, ZhiguangKim, Young-PilWang, ZhiweiKim, YunkyungWang, ZhongliKim, Yun-SoungWang, ZhouyiKimura, TakehideWang, ZhuKinar, Nicholas J.Wang, ZhuweiKindu, MengistieWang, ZiranKinet, DamienWang, ZixiongKing, ChristineWan-Wendner, LinKing, DanielWarczek, JanKing, Juliet L.Ward, Jamie A.King, Scott A.Warfield, Angus D.Kingsley, J. DerekWarne, DavidKingston, AndrewWarren-Smith, StephenKintzios, SpyridonWarrick, PhilipKiourt, ChairiWarriner, KeithKirby, AndrewWashizawa, YoshikazuKirchner, JensWasiak, AndrzejKirchschlager, FlorianWasilewski, TomaszKireev, DmitryWasisto, Hutomo SuryoKirillova, Nataliya (N.P.)Waslander, Steven L.Kirk, AndrewWasmeier, PeterKirov, RoumenWasserzier, ChristophKirsanov, DmitryWasswa, JosephKiryukhantsev-Korneev, Ph.V.Wasylka, Justyna PłotkaKisala, PiotrWatanabe, EiichiKisiel, TomaszWatanabe, YasuakiKisielewicz, TomaszWatanabe, YasumasaKiss, OrsolyaWataru, SatoKiss, RainerWatkins, Anthony NealKiss, Rita M.Watlet, ArnaudKissinger, ThomasWatson, DesKitagawa, JiroWatson, Elizabeth BurkeKitajo, KeiichiWatson, RobertKitano, ClaudioWatts, AdamKitazumi, YukiWatts, SimonKitrungrotsakul, TitinuntWebb, AnnKiyono, KenWebb, R. ChadKizilova, NatalyaWebber, JulianKlakow, DietrichWeber, AndersonKlancar, GregorWeber, JürgenKlein, BerndWeber, RobertKlein, ItzikWeber, Silke Anna TheresaKlein, LeventeWeddell, Stephen JohnKlein, RenataWegiel, TomaszKlein-Paste, AlexWęglarski, MariuszKleinschmidt, JoãoWęglarz, ArkadiuszKlem, KarelWęglowski, MarekKlich, SebastianWehn, NorbertKlichowski, MichałWehner, MichaelKliks, AdrianWei Tan, Edward KahKlimaszewski, JanWei, ChengliKlimaszewski, KrzysztofWei, DunwenKlimov, AleksandrWei, FengKljusurić, Jasenka GajdošWei, GangKlomp, MatthijsWei, GuohuaKłonica, MariuszWei, HaoyunKlose, AndrewWei, Hua-liangKłosowski, GrzegorzWei, JunhongKłosowski, ŁukaszWei, LeiKlouček, TomášWei, MengKlueh, UlrikeWei, MenglianKluge, FelixWei, MinKlum, MichaelWei, QingyangKlun, MatejaWei, TaoKmoch, AlexanderWei, XiongKmon, PiotrWei, XiukunKnaflitz, MarcoWei, YangKnap, LechWei, YanliKnap, WojciechWei, ZhenboKnauth, StefanWeidinger, TamásKnechtle, BeatWeigel, RobertKnipfer, ChristianWeihnacht, ManfredKnopp, AndreasWeijtjens, WoutKnyaz, VladimirWeill-Duflos, AntoineKnypiński, ŁukaszWeinberg, MarcKo, ByoungChulWeiner, PascalKo, HoonWeingaertner, TimKo, Hyoung HoWeisong, LiuKo, JeongGilWeiss, Anthony J.Ko, Yeong HwanWeissman, MichaelKobayashi, FutoshiWeitzen, Jay A.Kobayashi, MakikoWeizman, YehudaKober, Vitaly I.Welearegay, Tesfalem GeremariamKobetičová, KláraWeller, AndreasKoblauch, HenrikWells, LeeKoblischka, Michael R.Wen, ChangbaoKobusińska, AnnaWen, Chih-YuKoc, BurhanettinWen, ChunyiKoç, OkanWen, GuanghuiKocer, Basaran BahadirWen, HaoranKoceva, Aleksandra RashkovskaWen, HongKoch, AlexanderWen, JianmingKoch, ReinhardWen, WeiKochanowicz, MarcinWendel, JanKochańska, IwonaWendzel, SteffenKochhar, AbhayWeng, ShunKocian, AlexanderWeng, WubinKocifaj, MiroslavWenger, ChristianKocik, MarekWennmalm, StefanKociuba, WaldemarWenskovitch, JohnKockum, Anton FriskWeres, JerzyKocsis, ImreWerhahn, OlavKodet, JanWerneck, MarceloKodors, SergejsWerner, ChristianKoech, RichardWernimont, Susan M.Koelpin, AlexanderWesarg, StefanKoerner, Lucas J.Wesolowski, KrzysztofKoeva, Mila NikolaevaWesołowski, MarekKoganezawa, KoichiWest, AndrewKoh, AhyeonWestberg, JonasKoh, Amanda S.Westbrook, PaulKoh, ElizabethWesterdahl, DaneKoh, Jae ChulWestern, DavidKoh, JaehanWestwick, DavidKoh, Seok-JooWetherley, ErinKohidai, LaszloWhang, MincheolKohli, UtkarshWhitehill, AndrewKohut, PiotrWhitelaw, GailKoike, MariWhiteley, HughKoike, YasuharuWiak, SlawomirKoike, YoshikazuWiczyński, GrzegorzKoit, MareWiderska-Chadaj, Zaneta S.Kojima, RyosukeWiegerink, RemcoKok, EllenWielgosz, MaciejKokado, KentaWieringa, FokkoKokkonen, TeroWierzba, PawełKokubun, TakanoriWierzbicki, DamianKołakowska, AgataWierzbicki, SławomirKolakowski, JerzyWijesingha, JayanKolakowski, MarcinWijesinghe, Ruchire ErangaKolar, DavorWijk, Kasper VanKolcun, RomanWijnands, Jasper S.Kolios, StavrosWijnhoven, RobKolivoška, ViliamWiktorski, TomaszKolláth, ZoltánWilczyński, JacekKöllner, AntonWilkins, RossKolokoussis, PolWilkinson, BenKoltermann, JanWilkman, OlliKołtunowicz, Tomasz NorbertWilkowski, ArturKolwas, MaciejWill, ChristophKomaris, Dimitrios SokratisWillemsen, ThomasKomarov, MikhailWillert, AndreasKomarova, Natalia V.Williams, Gustavious PaulKomerath, Narayanan M.Williams, ManodKomori, MochimitsuWilliams, RyanKomorska, IwonaWilliams, SimonKompatsiaris, IoannisWilliams-Bell, MichaelKon, Mark A.Willis, BrianKonar, ArkaprabhaWillis, Katharine S.Koncar, VladanWilmer, ChristopherKonefał, MarekWilson, Alphus D.Kong, BaileyWilson, Christopher G.Kong, Byeong YongWilson, ColinKong, De-MingWilson, DanKong, Kien VoonWilson, Stephen G.Kong, LingfuWilson, William C.Kong, LingjieWilt, Kyle R.Kong, MeiweiWinczek, JerzyKong, TiantianWindholz, LaurentiusKong, XiangboWindolf, MarkusKong, XiangxiongWiniarski, TomaszKongsro, JørgenWiniczenko, RadosławKönig, CarolineWinkler, Thomas E.Kono, YasuyukiWińska, MałgorzataKononikhin, Alexey S.Wiora, AlicjaKonovalov, Cepгeй ИльичWirth, DagmarKonovalov, Dmitry A.Wisniewski, AdamKonovalov, SergeyWiśniewski, RemigiuszKönsgen, AndreasWiśniowski, PiotrKonstantakopoulos, Ioannis C.Wissman, JamesKonstantinos, FysarakisWissmeyer, GeorgKonstantinos, VotisWitczak, MarcinKontar, EduardWitczak, PiotrKontar, Raed AlWitkowski, MarcinKontogiannis, SotiriosWitkowski, UlfKontoni, Denise-Penelope N.Witowski, JanKontopoulos, IoannisWitte, Hartmut F.Konyakhin, Igor A.Wixted, AndrewKonys, AgnieszkaWodarski, PiotrKoo, ChiwanWojciechowski, AdamKoohbor, BehradWojciechowski, JerzyKooman, Jeroen P.Wojciechowski, SzymonKoparan, CengizWójcik, DariuszKopecký, DušanWojcik, GrzegorzKopeika, NatanWójcik, Grzegorz MarcinKopparthy, Varun LingaiahWójcik, KrzysztofKoprda, StefanWójcikowski, MarekKoprinkova-Hristova, PetiaWojnar, PiotrKoral, CanWojnowski, WojciechKorbel, PiotrWojtanowski, JacekKorbiel, TomaszWojtas, JacekKordestani, MojtabaWójtewicz, SzymonKordos, DamianWojtowicz, KonradKordoš, MarcelWójtowicz, RyszardKordos, MiroslawWolanśki, WojciechKordov, KrasimirWoldemariam, Gezahegn WelduKore, RajkumarWolffsohn, James J.Koren, KlausWöll, DominikKoreň, MilanWollmann, PhilippKorka, ZoltanWolny, AdaKorkmaz, EmrullahWolny, TomaszKormaníková, EvaWon, Jong-HoonKorneev, DenisWondrak, ThomasKorobiichuk, Igor (Poland)Wong, Boon-SengKorobiichuk, Igor (Russia)Wong, David Shan-HillKorodi, AdrianWong, Duo Wai-ChiKorolkov, Ilya V.Wong, HangKorotcenkov, GhenadiiWong, Kainam ThomasKorzeniewska, EwaWong, Lawrence L.P.Korzun, DmitryWong, Ling TimKos, AndrejWong, Terence T. W.Kos, AntonWongkasem, NantakanKos, MarkoWoo, HanwoolKosa, GaborWoo, HongukKosacka-Olejnik, MonikaWoo, KyooheeKosaka, Priscila M.Woo, Wai LokKościelny, Jan MaciejWood, AihuaKose, UtkuWood, AndrewKosec, TadejaWood, Richard L.Kosman, JoannaWood, Stephen L.Kosmas, PanosWoodhead, IanKošnar, KarelWoon, Wei-YenKost, GabrielWorkman, Craig D.Kostal, PeterWorsey, Matthew ToKostek, BożenaWotzka, DariaKostelecký, JakubWoubishet, TaffeseKostin, Vladimir NikolaevichWouters, Michael J.Kostoglou, KyriakiWouters, PeterKostrzewa, DanielWoyessa, GetinetKostrzewski, MariuszWozniak, MarcinKostyła, PawełWright, Ruishu F.Koszela, KrzysztofWright, ThomasKot, SebastianWróbel, KrzysztofKotelba, AdrianWrobel, PiotrKothe, SteffenWronkowicz-Katunin, AngelikaKotis, KonstantinosWrzesiński, GrzegorzKotnala, AbhayWtorek, JerzyKotropoulos, ConstantineWu, BenKotsiantis, SotirisWu, BianKotteda, V. M. KrushmaraoWu, BinKotyński, RafałWu, CelimugeKotzanikolaou, PanayiotisWu, ChenKou, ChangjiangWu, ChenshengKou, GangWu, Ching-ChouKouba, JanWu, Chun HoKoubias, StavrosWu, Chung-Tse MichaelKouchaki, SamanehWu, ChunleiKoucká Knížová, PetraWu, ChunshengKoudelka, PetrWu, Chyan-ChyiKoudelková, ZuzanaWu, DajunKoudouridis, Georgios P.Wu, DapengKouh, TaejoonWu, DufanKoulamas, ChristosWu, FalinKounelis, IoannisWu, FanKoustoumpardis, PanagiotisWu, GuoqiangKoutras, AthanasiosWu, GuoZhongKoutras, ThanasisWu, HaibinKoutsokeras, LoukasWu, Hai-ChenKoutsouras, Dimitrios A.Wu, HangbinKovač, OndrejWu, HejunKovacević, TonkoWu, Hong RenKovacic, IvaWu, HongminKovács, PéterWu, HuaKovács, ZoltánWu, HuamingKovácsházy, TamásWu, Ja-lingKovalev, Valeri I.Wu, Janne WhaKovanič, ĽudovítWu, Jason (Dayong)Kovář, JiříWu, JiajunKovari, AttilaWu, JianKoven, Steven G.Wu, JianboKowalczyk, AgataWu, JianfengKowalczyk, CezaryWu, JiankangKowalczyk, KamilWu, JianqingKowalska, Aneta AnielaWu, JiayangKowalski, DariuszWu, JingKowaluk, TomaszWu, JinsongKowzan, GrzegorzWu, KaiKozak, DražanWu, KeyuKozak, MaciejWu, Li-ChungKozakevicius, Alice J.Wu, LinKozawa, YuichiWu, LingtaoKozhoridze, GiorgiWu, LixiangKozierkiewicz, AdriannaWu, LyndiaKozioł, MichałWu, MinKozuba, JaroslawWu, MoleiKraatz, Heinz-BernhardWu, MousongKraemer, Karl MichaelWu, Nan (Canada)Krafft, FriederWu, Nan (China)Kraft, MarekWu, PenghaiKraft, MirkoWu, QiKrajewski, AdamWu, QingcunKrajewski, PiotrWu, ShangquanKraleva, RadoslavaWu, ShengbiaoKramer, RickWu, ShengchuanKrasilov, ArtemWu, Shin-TsonKraska, ThorstenWu, Shuen-DeKrasoulis, AgamemnonWu, ShuicaiKrasovsky, TalWu, ShunanKrasteva, VesselaWu, TailinKrasuski, KamilWu, TonghuaKratochvil, TomasWu, WanqingKratzke, NaneWu, WeiKraus, MartinWu, WeidongKrause, Hans-JoachimWu, WengangKrause, PawełWu, XiangKrausz, NiliWu, XianjunKrauze, PiotrWu, XiaodongKravari, KalliopiWu, XiaofengKrawczuk, MarekWu, XinjunKrawczyk, PiotrWu, XiongbinKrawec, Walter O.Wu, XuejianKrcum, MajaWu, XueruiKrebsz, MelindaWu, XuezhiKrejcar, OndrejWu, YichenKrejsa, JiříWu, YiruiKrell, Mario MichaelWu, YuankaiKrenicky, TiborWu, YuanmingKrewinkel, RobertWu, YuanweiKrichen, MoezWu, YuanxinKrieger, AxelWu, YueKriegner, DominikWu, YuqingKrintz, ChandraWu, ZhanjunKrishnan, SiddharthWu, ZhengKrishnaswamy, BhuvanaWu, ZhiyiKristian, YosiWu, ZhongKristoffersson, AnnicaWu, ZuchengKrivanek, VaclavWuenschell, JeffreyKřivda, VladislavWurz, Marc ChristopherKrivetskiy, ValeriyWychowaniec, JacekKrobka, NikolayWyffels, FrancisKroczak, RafalWymeersch, HenkKról, AleksanderWymore, Mathew L.Krol, MichałWysocki, MarianKrólczyk, JolantaWyszkowski, MirosławKrólicka, AgnieszkaWytrębowicz, JacekKromanis, RolandsWyver, ShirleyKropf, JohannesXavier, António M.S.Kropotov, JuriXavier, JollyKrstulović-Opara, LovreXavier, MiguelKrtalić, AndrijaXavier-de-Souza, SamuelKrueger, EddyXhafa, FatosKrug, SilviaXi, PengChengKrüger, BjörnXi, RuijieKruglov, RomanXi, XiaoliKrupitzer, ChristianXi, XugangKrusienski, Dean J.Xia, ChunleiKrygier, JaroslawXia, ChunmingKrylov, SlavaXia, DahaiKrystofiak, TomaszXia, DunzhuKryvinska, NataliaXia, FengKrzaczek, PawełXia, LinyuanKrzan, GrzegorzXia, WenfengKrzemiński, JakubXian, XiaojunKrzeszowski, TomaszXiang, ChaoqunKrzywiecki, ŁukaszXiang, DeliangKrzyzak, AdamXiang, DongKrzyżak, Artur TadeuszXiang, HengxueKtena, AphroditeXiang, Hong-JunKu, TaeseoXiang, JiaweiKuan, Yean-DerXiang, LiangzhongKuanar, ShibaXiang, RongKuang, DengfengXiang, WangKuang, ShaolongXiang, WeidongKuang, YangXiang, XianboKuang-Yi, HsuXiang, YanKuangyu, ZhengXiang, YujiangKubacki, RomanXiang, ZhiyuKubasov, IlyaXiao, ChiweiKubera, ElżbietaXiao, FuyuanKubiak, IreneuszXiao, GuijianKubicek, JanXiao, JielingKubik, AndrzejXiao, JunKubík, ĽubomírXiao, LiangKubik, ZdenekXiao, QinkunKubina, RobertXiao, RuyaKubinyi, MiklósXiao, XuesuKubo, NobuakiXiao, YueleiKučera, ErikXiao, ZhitaoKucharska, KarolinaXiao, ZhuKucharski, JanXiao, ZhuolingKuczynski, Anika KuXiaohua, ZhaoKuczynski, SzymonXiaopeng, ShaoKudela, PawełXiaoying, OuyangKudiiarov, ViktorXie, GuotaoKudriashov, VolodymyrXie, HongtaoKudrna, PetrXie, HualongKudryashov, Sergey I.Xie, JingKueppers, BastianXie, JunqiKuester, MicheleXie, KunKuestner, ThomasXie, LiangKuffer, MonikaXie, LingxiKugler, SzymonXie, LipingKuhar, AlešXie, LiyuKühbeck, ThomasXie, PingKühling, InsaXie, QiaoyunKuhlmann, KarstenXie, RuiKuhn, HartmutXie, ShangranKuiper, HansXie, ShengkunKuitche, Maxime Alex JuniorXie, ShiyuKujawska, MałgorzataXie, XiufengKukaev, AlexanderXie, YuedongKukla, MateuszXie, YuliangKukli, KaupoXie, ZhijianKulau, UlfXie, Zhong-RuKulesza, WlodekXin, GuiyangKulesza, ZbigniewXin, XiangjunKulhandjian, HovannesXing, ChenKulich, MiroslavXing, FeiKulinich, SergeiXing, MengdaoKulka, JozefXing, YanqiuKulkarni, AtulXing, YuxinKulkarni, TanmayXingquan, CaiKulyukin, Vladimir A.Xinyi, TangKumar K B, VinayaXiong, BinKumar Khambampati, AnilXiong, FengchaoKumar, AbhishekXiong, GuangmingKumar, AmitXiong, HuKumar, GaneshXiong, JiaqingKumar, ManojXiong, JieKumar, PankajXiong, JipingKumar, PawanXiong, KaiKumar, PiyushXiong, LinKumar, PradeepXiong, ShupingKumar, SandeepXiong, WeiKumar, SanjeevXiong, WenKumar, SatishXiong, XiaogangKumar, ShailabhXiong, ZehuiKumar, ShitijXiong, ZhihuaKumar, TaneshXiong, ZhiweiKumar, VikasXiros, NikolaosKumar, VineetXu, BaodongKumara, IndikaXu, ChangqiaoKumavor, Patrick D.Xu, ChaoKumhála, FrantišekXu, Chi (China University of Geosciences, China)Kuncová, GabrielaXu, Chi (Chinese Academy of Sciences, China)Kundu, ShamikXu, ChongKundu, TribikramXu, ChunyanKunicki, MichałXu, Dong-shengKunimori, HirooXu, FengqiuKuo, CalvinXu, FujunKuo, Chi-ChingXu, GuangquanKuo, Hsien-WenXu, GuobaoKuo, Tsung-TingXu, GuoshengKuo, Wen-chengXu, HaitaoKuok, Sin-ChiXu, HaoKuo-Liang, ChungXu, HongleiKupidura, PrzemysławXu, HongliKuppannagari, Sanmukh R.Xu, HuapingKuramitz, HidekiXu, HuiKuras, PrzemysławXu, JiaKurc, BeataXu, JianKurdi, Fayez TarshaXu, JiangtaoKurdi, HebaXu, JiaqiangKuremoto, TakashiXu, JiawenKuriakose, MajuXu, JingKurihara, ToruXu, JingzhongKuriqi, AlbanXu, JuanjuanKurita, KazunariXu, JunliKurlyandskaya, Galina V.Xu, Kai (China)Kurte, KuldeepXu, Kai (USA)Kurtser, PolinaXu, KaichenKuś, WacławXu, Kai-DaKusari, ArpanXu, KaikaiKusetogullari, HuseyinXu, KailiKushch, SergiiXu, KailiangKuśmierek-Tomaszewska, RenataXu, KeKusters, IljaXu, KeleKusumoto, TamonXu, KuiwenKutafina, EkaterinaXu, Kun (Beihang University, China)Kutila, MattiXu, Kun (University of Chinese Academy of Sciences, China)Kutser, TiitXu, LangKuusik, AlarXu, LeiKuwatani, TatsuXu, LijunKużelewska, UrszulaXu, LinchuanKuznetsov, Artem A.Xu, LinfengKuznetsova, Iren E.Xu, LingweiKwak, Bong SeopXu, LinruKwak, Keun-ChangXu, LishengKwan, ChimanXu, MinpengKwapisz, AgnieszkaXu, NanKwoczyńska, BogusławaXu, RongqingKwon, Hyuck-InXu, RuiKwon, HyungjuXu, ShengKwon, JaerockXu, ShengliKwon, JinhyeongXu, ShengyongKwon, JunseokXu, ShicaiKwon, Ki-ChulXu, ShoujunKwon, Seong JungXu, TaoKwon, Soon-HongXu, TianhuaKwon, Soon-HyungXu, TianjianKwon, SoonilXu, TingtingKwon, YounggooXu, TuanweiKwon, YoungMinXu, WeiKwon, Yung-KeunXu, WeiheKyamakya, KyandoghereXu, WeitaoKyrarini, MariaXu, Wen TaoKyriakou, Georgios A.Xu, WenjunKyriakou, KalliopiXu, XiangKyriazakos, SofoklisXu, XiangyangLa Deda, MassimoXu, XiaochuanLa Foresta, FabioXu, XiaominLa Forgia, DanieleXu, XiaowenLa Russa, MauroXu, YangLa Russa, Mauro FrancescoXu, YingLa Spada, LuigiXu, YongLa Tona, GiuseppeXu, YongfengLabant, SlavomirXu, YongmingLabiod, HoudaXu, YougenLabra Gayo, José EmilioXu, YuanLabun, JánXu, YuandongLabus, AleksandraXu, YuquanLabzovskii, Lev D.Xu, YushengLacaze, BernardXu, ZhenLacerda, Márcio JúniorXu, ZhengguoLacerda, Rodrigo GribelXu, ZhenlongLachapelle, GerardXu, ZhezhuangLacidogna, GiuseppeXu, ZhihuaLacik, JaroslavXu, ZhongyangLackovic, IgorXuan, QiLaefer, DebraXuan, ShouhuLafata, PavelXuan-Mung, NguyenLaffranchi, MatteoXue, FanLafruit, GauthierXue, JinlinLagaňa, RastislavXue, KunLagarde, FlorenceXue, NingLaghrouche, MouradXue, TianyuLagkas, ThomasXue, XiaoyongLago, PaulaXue, XinyuLago, Tiffany R.Xue, YifeiŁagowski, PiotrXue, ZhidongLagrosas, NofelXylomenos, GeorgeLaguarda-Miró, NicolásYadav, AnimeshLagundzin, DraganaYadav, Hemraj M.Lahmer, TomYadav, NandakishorLahmiri, SalimYagati, Ajay KumarLai, Chao-SungYaici, WahibaLai, Chiu-KengYakavets, IlyaLai, Chow YinYalikun, YaxiaerLai, CristianYamada, AkiraLai, JinxingYamada, IchiroLai, JizhouYamagishi, KentoLai, Loi LeiYamagiwa, ShinichiLai, PuxiangYamaguchi, TomoyukiLai, Ying-ChihYamakawa, ToshitakaLai, YingxuYamamoto, HirokazuLaidig, DanielYamamoto, TatsuyukiLain, Jenn-KaieYamane, KatsuLaín, SantiagoYamashita, MegumiLaine, ChristopherYan, BingxiLajevardipour, AlirezaYan, JiaLajos, NagyYan, JinLalalouna, DavidYan, JingLalbakhsh, AliYan, JiningLaliberte, JeremyYan, JunkunLalit K., MesthaYan, KeLall, Gurprit S.Yan, NanLallana, Pedro C.Yan, SenLallart, MikaelYan, ShengLallas, Efthymios N.Yan, WeiLam, BhanYan, XiaofeiLam, Hak KeungYan, YajingLam, Soi HoiYan, YuLam, Wei HaurYan, ZhengLambers, KarstenYan, ZhongjiangLambert, NicholasYanambaka, Venkata PrasanthLamberti, NicolaYanase, TakashiLamberti, Vincent E.Yanaseko, TetsuroLambertini, AlessandroYang, AixiaLambert-Torres, GermanoYang, BinLambin, PhilippeYang, CeLambrinoudakis, CostasYang, ChengLamonaca, FrancescoYang, Cheng-FuLan, JianglinYang, Chia-MinLan, RushiYang, Chih-ChinLan, ShengchangYang, Ching-YuLancashire, HenryYang, ChunhuaLancaster, DavidYang, DachiLanceros-mendez, SenentxuYang, DiLancini, MatteoYang, DiangeLanconelli, NicoYang, DingtianLandi, CarmineYang, DongLandi, MarcoYang, DongkaiLandi, SilviaYang, DujuanLandoni, MatteoYang, FanLandowska, AgnieszkaYang, FangLandulfo, EduardoYang, FeiLane, AndrewYang, FengLane, DavidYang, GengLane-Serff, GregoryYang, GongpingLang, FengkaiYang, GuanciLang, Hans PeterYang, GuangLang, Jochen (Canada)Yang, GuisongLang, Jochen (France)Yang, GuoanLang, MichaelYang, GuowuLang, NorbertYang, HaifengLang, TingtingYang, HangLang, WalterYang, HaoLange, EwaYang, Hee-DeokLange, RalphYang, HengLangford, ZacharyYang, HongtaLangley, PhilipYang, HuaiLangone, RoccoYang, HuaiyuLanza, GiselaYang, HuiLanza, Marcel BahiaYang, JasonLanzone, JacopoYang, JiachenLapington, JonYang, JiamiaoLapkovskis, VjaceslavsYang, JianLaplace, ThibaultYang, JianjunLapray, Pierre-JeanYang, JianxiaoLara Molina, Fabian AndresYang, JiaqiLara, EvangelinaYang, JinLara-Cueva, Román AlcidesYang, JingyuLarge, JamesYang, Jinn-MinLarion, MioaraYang, JinxiangLarios, Diego FranciscoYang, Jong-RyulLarisa N., SidorovaYang, Judy P.Larsen, YngvarYang, KailunLas-Heras Andrés, FernandoYang, KundeLasheras, AndoniYang, LeLas-Heras-Casas, JesúsYang, LiangjingLashkari, Arash HabibiYang, LidongLashkari, BahmanYang, LiuLaski, Pawel AndrzejYang, Ming (China)Lasky, TyYang, Ming (USA)Lasri, TuamiYang, MingweiLastra, MiguelYang, PanlongLászló Orbán, CsambalikYang, Po-ChihLászló, MakraYang, QiLata, MichaelYang, QiangLatella, ChristopherYang, QuanlongLatif, TahmidYang, RuiLatorrata, SaverioYang, RuitaoLatré, StevenYang, RuixinLatychevskaia, TatianaYang, SejungLau, Daniel LeoYang, ShixiLau, MichaelYang, ShufanLaudani, AntoninoYang, ShuhuiLaufer, JanYang, ShupengLaukkanen, Anne-MariaYang, ShuqingLaupré, Gabriel FrançoisYang, SihuaLaureti, StefanoYang, TiannanLaurin, JérômeYang, WeiLaurini, EleonoraYang, Wei-GangLaurini, RobertYang, Wei-hsiungLautner, GergelyYang, WenchengLavacca, Francesco G.Yang, WenwuLavadiya, Dayakar NaikYang, WenxianLavi, BahramYang, WoosungLavorgna, MarinoYang, XiLavrenko, AnastasiaYang, Xiao-longLavric, AlexandruYang, XinLaw, DavidYang, XinghuaLawman, SamuelYang, XinmaiŁawniczak, PawełYang, XuLawnik, MarcinYang, XueLay-Ekuakille, AimeYang, XusanLazar, Adriana VulpoiYang, YangLazar, IwonaYang, YansongLazaridis, PavlosYang, YiLázaro, AntonioYang, YilongLázaro, JoséYang, Yuan-SenLázaro, María TeresaYang, YunfanLazaroiu, GheorgheYang, YushiLazarov, AndonYang, ZhaohuiLazarov, Andon DimitrovYang, ZhenchuanLazarowska, AgnieszkaYang, Zhi ChunLazarus, NathanYang, ZhiboLazik, DetlefYang, ZhileLazzaretti, André EugênioYang, ZhixinLazzaroni, MassimoYang, ZhongliangLe Breton, Jean-marieYanine, Franco FernandoLe Bris, ArnaudYankov, Metodi PlamenovLe Moal, PatriceYannis, GeorgeLe Thai, DuyYano, InacioLe, Dinh ThuanYano, KazuyoshiLe, Duc VanYao, BaichengLe, Nam TuanYao, Da-JengLe, NamTuanYao, DongdongLe, Nguyen Quoc KhanhYao, HaipengLe, Trung-KienYao, JiafengLe, TuongYao, JunLeal-Junior, ArnaldoYao, LeLeba, MonicaYao, LingLebedev, SergeyYao, Xin-WeiLebègue, EstelleYapici, Murat KayaLebel, KarinaYaroshchuk, IgorLebental, BérengèreYasa, Immihan CerenLeBlanc, Roger M.Yasrab, RobailLebon, FrédéricYasukawa, TomonoriLeca, Cristian LiviuYasumoto, MasatoLeccese, FabioYasuoka, YumiLech, PiotrYasyukevich, YuryLech, RafalYatabe, RuiLechuga, YolandaYatchev, IvanLecieux, YannYau, YungLeder, NenadYazdani Asrami, MohammadLedo, AnaYazdanpanah, HamedLeduc, DamienYe, ChaofengLeduc, DominiqueYe, EnyiLee, AhyoungYe, FanLee, BumshikYe, HaihangLee, Byeong HaYe, HangLee, Byung ChulYe, JiaxingLee, ByunghunYe, JingLee, ByungKwanYe, LingjianLee, ChanghoYe, WeilinLee, Chang-RyeolYe, YalanLee, Chao-HsienYe, YuLee, ChengkuoYe, YuhangLee, Che-RungYe, YunpengLee, Chou-YuanYe, ZhenLee, Dae YoungYeager, John D.Lee, Dae-HyunYeh, Bih-ChyunLee, Dong MyungYeh, Chien HungLee, DonghunYeh, Chih-FengLee, Eui ChulYeh, Kuo-HuiLee, EunsongyiYeh, Sheng LihLee, Geon JoonYelamarthi, KumarLee, Gi-JaYe-Lin, YiyaoLee, Gwang GooYemelyanov, Vitaliy A.Lee, Gyu MyoungYen, Chen-wenLee, GyudoYen, Li-HsingLee, GyumyoungYen, Yi-KuangLee, Hae YoungYeo, HongLee, HaeyoungYeo, Joo ChuanLee, Han EolYeo, JunhoLee, Han LimYeoh, Justin K.W.Lee, Hee-JoYeom, EunseopLee, HoonYeom, JunghoonLee, HoseonYeom, SeokwonLee, Hsiang-ChiehYeong, Wai YeeLee, Huang-ChenYerima, Suleiman Y.Lee, HwasungYerolatsitis, StephanosLee, Hyo JongYesilkoy, FilizLee, HyonCheolYi, ChangyanLee, HyosangYi, HwangLee, Hyunjoo J.Yi, JianxinLee, HyunseopYi, WeiLee, HyunsooYi, YougenLee, Il-GuYi, ZaoLee, In SooYi, ZhengkunLee, Jae MinYigitcanlar, TanLee, Jae WoongYildirim, TanjuLee, JaehoonYilmaz, MehmetLee, JaewooYilmazoglu, OktayLee, JeffYim, ChanghoonLee, Jeong SuYin, ShenLee, Jeong WooYin, TaoLee, JihoonYin, XiaobinLee, Jin-ShyanYin, XiaokangLee, JinsilYin, YongkaiLee, Jong YongYin, ZhenLee, JonghunYinka-Banjo, ChikaLee, Jong-SubYioultsis, Traianos V.Lee, Jong-WookYitayew, Temesgen GebrieLee, Joon-YongYitmen, IbrahimLee, Joshua En-YuanYizhuo, ZhangLee, Jun SeokYngvesson, SigfridLee, Jung KeunYo, TanakaLee, Jung-RyunYodo, NitaLee, JunseopYokoyama, TakashiLee, Ju-YiYoo, ByungseokLee, JuyongYoo, ChuckLee, KeekeunYoo, Jae-HyuckLee, KeunhwaYoo, JaehyunLee, Keun-HyeungYoo, Jang-HeeLee, KooktaeYoo, Jung SunLee, KyoeRehYoo, SeehwanLee, Kyoung-joungYoo, Seong-eunLee, Kyu-haengYook, Jong-GwanLee, KyumanYoon, DukyongLee, KyungroulYoon, GiwanLee, Li-AngYoon, HyosangLee, MeonghunYoon, HyoseokLee, Moon-KyuYoon, HyungchulLee, Myeong-jinYoon, Hyungchul (Henry)Lee, OnseokYoon, HyungkooLee, PosenYoon, Ji-WookLee, ReginaYoon, SangpilLee, Sang HyunYoon, SeokhoonLee, Sang JunYoon, Yeo-SunLee, Sang-GonYoon, Yong-IkLee, SanghyoYordanova, KristinaLee, Sang-MaeYoshida, HiroshiLee, SangminYoshikawa, HirokiLee, SangukYoshimoto, ShunsukeLee, Sang-WoongYoshino, ToshihikoLee, SeokjunYoshinobu, TatsuoLee, SeongwookYoshitake, YasuhideLee, SeulkiYou, Cheol-HwanLee, SeungYou, HeecheonLee, Seung HeeYou, IlsunLee, Seung-BeckYou, LiLee, SeunghwanYou, ShutangLee, Shie-JueYou, XingeLee, Suk JinYou, Young-HwanLee, SukhoYoun, Ik-HyunLee, Sung SikYoun, InchanLee, Sung-HakYoun, JonLee, SunghoYoun, WonkeunLee, SunminYounes, RabihLee, TaekYoung, Timothy M.Lee, TempleYousaf, JawadLee, Tian-FuYousefi, BardiaLee, WinsonYoussef, AliLee, Won ChulYoussef, AmrLee, WoncheolYu, Anthony W.Lee, WonseokYu, AnxiLee, Woong-HeeYu, CaoyangLee, XuhuiYu, ChenLee, Yang SunYu, FabiaoLee, YangsunYu, Feng-YihLee, Yen ChienYu, HaiLee, Yong OhYu, HakkiLee, Yong-WookYu, HaofeiLee, Young-SilYu, HongfaLee, Yu-ChiYu, HongyuLee, Yun-JuYu, HuangchaoLee, ZhongPingYu, HuapengLees, John E.Yu, JaehyungLefik, MarekYu, JiayongLegg, MathewYu, JingangLehikoinen, AnttiYu, JingjinLehmann, ArminYu, JinhuaLei, HongjiangYu, JunzhiLei, JinchengYu, LianDongLei, TaoYu, LiangLei, WenyingYu, LiangjiangLei, YingkeYu, NingboLeightley, DanielYu, QingminLeineweber, MatthewYu, ShaodeLeinonen, MarkusYu, ShuoLeister, WolfgangYu, TianqiLeite Filho, Waldemar C.Yu, Ting-ToLeitgeb, MarkusYu, WenLeithardt, Valderi Reis QuietinhoYu, WenbinLeitner, GerhardYu, XiaoLeivadeas, ArisYu, XiaojunLeiviskä, KaukoYu, XiaopengLemaire, JoëlYu, XunLemmens, MathiasYu, YangLenac, KristijanYu, YanguangLenart, MałgorzataYu, YanweiLenci, StefanoYu, ZhibinLeng, JiewuYuan, BingLeng, LuYuan, ChengxunLeng, PeiYuan, HongyongLeng, YuxinYuan, HuaLengden, MichaelYuan, JinhuiLennox, BarryYuan, LeiLenton, GavinYuan, MingLentzas, AthanasiosYuan, WeiLenzo, BasilioYuan, WeijieLeo, MarcoYuan, XiaoLeón Espinoza, Edgar AlejandroYuan, XiaofengLeón, JulioYuan, YeLeón, OlgaYuan, YihongLeonardi, FrancescaYuan, ZhenLeonardi, LucaYuan, ZhiqinLeonardi, MauroYuan, ZhongyunLeone, GiovanniYudin, DmitryLeoni, AlfieroYue, AlexLeoni, ClaudiaYue, JingLeonowicz, ZbigniewYue, John K.Leontidis, GeorgiosYue, JunLepadatu, DanielYue, TaoLeporini, MariellaYue, YueminLerch, AlexanderYuen, ChauLerga, JonatanYuen, Ka-vengLerma, ClaudiaYufera, AlbertoLeroux, PaulYuffa, Alex J.Leru, PolianaYuizono, TakayaLesecq, SuzanneYun, Byoung-JuLesiak, PiotrYun, DaqingLeslie, GraceYun, Hyun DoLeśniewicz, AnnaYun, Ji-HoonLesselier, DominiqueYun, KiminLesser, MichaelYun, TingLete, CeciliaYunusa-Kaltungo, AkiluLetia, Tiberiu StefanYurtsever, EkimLettieri, StefanoYuste Delgado, Antonio JesúsLeu, Fang-YieZabala, MichaelLeucci, GiovanniZaballos Diego, AgustinLeung Patrick, Hui ChiZabierowski, WojciechLeung, CarsonZaborowicz, MaciejLevine, JudahZabot, Giovani L.Levine, MindyZabotin, NikolayLevine, Zachary H.Zacharaki, Evangelia I.Levitan, JacobZacharis, Constantinos K.Levitt, Jonathan B.Zachsenhouse, MiriamLevski, DeyanZafra, Ángel RuizLevy, StevenZagajewski, BogdanLewandowicz, ElżbietaZaghloul, MohamedLewińska, PaulinaZagidullin, NaufalLewis, GregoryZagouras, AthanassiosLewis, QuinnZaharia, MihaiLeychenko, ElenaZaharieva, DessiLeylavi Shoushtari, AliZaimes, GeorgeLezzerini, MarcoZaitouny, AyhamLhotska, LenkaZaitsev, Boris D.Li, AlanZajc, MateaLi, AnlongZajec, BojanLi, BeiwenZakerzadeh, RanaLi, BinZakharov, Igor L.Li, Bor-RanZakharov, Oleg V.Li, BoyangZakinyan, ArthurLi, BuyinZakrzewska, KatarzynaLi, ChangZalewski, PawełLi, ChangjianZam, AzharLi, ChengdongZamani Meymian, NimaLi, ChengqingZambonin, Giuliano (Italy)Li, ChongZambonin, Giuliano (Sweden)Li, ChuanZambrana-Vinaroz, DavidLi, ChunguoZambrano, DavideLi, ChunxuZambrano-Martinez, Jorge LuisLi, CuiyunZamfira, Constantin SorinLi, DavidŻamojć, KrzysztofLi, De-junZamora, MarcoLi, DeminZamora, WillianLi, DeshiZamorska, JustynaLi, DongZampieri, NicolòLi, DongshengZampogna, AlessandroLi, DuoZampolli, StefanoLi, FanZamzmi, GhadaLi, FangyuZanardi, ChiaraLi, FashuaiZanchetta, GiulianoLi, FeiZandavi, Seid MiadLi, Feng (Qingdao Agricultural University, China)Zander, FabianLi, Feng (Xi’an Jiaotong University, China)Zandi, OmidLi, FenghuaZandonadi, Renata PuppinLi, FengqiangZanella, AlbertoLi, FrederickZang, XiaoqinLi, FuZangeneh-Nejad, FarzadLi, GenZankiewicz, AndrzejLi, GuofaZannis, TheodorosLi, GuoyuanZannone, NicolaLi, HaiyanZanut, AlessandraLi, HanchuanZappalà, GiuseppeLi, HaoZappatore, MarcoLi, HaojieZárate, Luis E.Li, HongZarayeneh, NedaLi, HongguiZarazaga, Francisco JavierLi, HongkunZareei, MahdiLi, HonglangZareinia, KouroshLi, HongqiangZarejousheghani, MashaalahLi, HongshengZariffa, JoseLi, HuafengZarifi, Mohammad H.Li, Hui (Beihang University, China)Zarpalas, DimitriosLi, Hui (China University of Geosciences, Wuhan)Zarpelão, BrunoLi, Hui (Chinese Academy of Sciences, China)Zarpelão, Bruno BogazLi, JiaZarri, Gian PieroLi, Jia-hanZarrin, HadisLi, JiamingZaťko, BohumírLi, JiangZaukuu, John-Lewis ZiniaLi, JianqingZaunseder, SebastianLi, Jianzhong (Guangdong University of Technology, China)Zavabeti, AliLi, Jianzhong (Northeastern University, China)Zavala-Rivera, PaulLi, JiayuZawada, KatarzynaLi, JieZawieska, DorotaLi, JihengZayas, Almudena DiazLi, JinZbořil, FrantišekLi, JingZdravevski, EftimLi, Jing SongZec, JoskoLi, JiweiZednik, RicardoLi, JunZefferer, ThomasLi, JunhuiZega, ValentinaLi, JunrongZegers, PabloLi, JunxinZeid, AbeLi, Kai ChunZeitoun, PhilippeLi, KaitaoZeleniakiene, DaivaLi, KaiweiZema, Nicola RobertoLi, KezhiZembaty, ZbigniewLi, KuiZemcik, PavelLi, LeileiZemouri, RyadLi, LiZeng, DawenLi, LiangZeng, HuanqiangLi, LijieZeng, JianwuLi, LinchaoZeng, LiminLi, Ling-YunZeng, NianyinLi, LixiangZeng, PengLi, LongZeng, RunqiangLi, LongbiaoZeng, XianyiLi, LongjiangZeng, ZhoumoLi, MaokunZennaro, Marco (Italy)Li, Miao (China)Zennaro, Marco (UK)Li, Miao (Switzerland)Zerhouni, NoureddineLi, MinZet, CristianLi, Ming-AiZeuch, SteffenLi, MingchaoZewdie, Ephrem TakeleLi, MingchuZeydan, EnginLi, Ming-FuZgłobicki, WojciechLi, Ming-HuangZha, FushengLi, NaipengZha, GangqiangLi, Nan (Australia)Zhai, ChaoLi, Nan (China)Zhai, LushengLi, PengZhan, RonghuiLi, Po-HsienZhan, WeiLi, QiangZhan, YinweiLi, QingZhang, A PingLi, QingliZhang, AnLi, RongpengZhang, BaochengLi, RuiZhang, BinLi, RuijunZhang, BingLi, ShandongZhang, BoLi, ShengZhang, BobLi, ShenghongZhang, ChaoLi, ShigangZhang, ChengLi, Shih-YuZhang, ChiLi, ShiyangZhang, ChunweiLi, ShuangZhang, CongchunLi, ShuangmingZhang, DaliLi, ShuguangZhang, DalinLi, ShunboZhang, DamingLi, ShuoZhang, DanLi, ShuxiaoZhang, DapengLi, SimengZhang, DeganLi, SongZhang, DejunLi, Sui-XianZhang, DequanLi, TaiyongZhang, DimingLi, TanZhang, DingguoLi, TengZhang, DingwenLi, TianchengZhang, DongLi, TianliangZhang, DongmingLi, TianxingZhang, DongyuLi, WanwanZhang, DouLi, WeiZhang, FanLi, WeideZhang, FengchunLi, WeihuaZhang, FuminLi, WeijieZhang, GongLi, WeiliZhang, GuangmingLi, WenzhuoZhang, GuangpenLi, XiZhang, GuogangLi, XiangZhang, GuojunLi, XianjuZhang, GuoqingLi, XiaoZhang, GuoxingLi, XiaocanZhang, GuyueLi, XiaodongZhang, HaiLi, XiaoluZhang, HaifengLi, XiaomengZhang, HaihongLi, XiaopengZhang, HaijunLi, XiaoqiangZhang, HanLi, XiaorunZhang, HaoLi, XinZhang, Haopeng (China)Li, XinbinZhang, Haopeng (USA)Li, XingZhang, HeLi, XinghuaZhang, HeminLi, XinyuZhang, HengcaiLi, XinzhongZhang, HengxuLi, XishengZhang, HeyeLi, XuanZhang, Hong (China)Li, XuechaoZhang, Hong (USA)Li, XuekeZhang, HongboLi, YaanZhang, HongpengLi, YanZhang, HuichunLi, YangZhang, HuilongLi, YanshengZhang, JanetLi, YantaoZhang, JiahuaLi, YeZhang, JianmingLi, YidaZhang, Jianzhong (Harbin Engineering University, China)Li, YinZhang, Jianzhong (Taiyuan University of Technology, China)Li, Ying-ChunZhang, JiashuLi, YingsongZhang, Jie (Newcastle University, UK)Li, YiyanZhang, Jie (The University of Sheffield, UK)Li, YongZhang, JingLi, YuhangZhang, JingdongLi, YuhongZhang, JinyangLi, YuhuaZhang, JizeLi, YulongZhang, Jun (Anhui University, China)Li, YundongZhang, Jun (National University of Defense Technology, China)Li, YunghuiZhang, KaihuaLi, Yung-HuiZhang, KehanLi, YunyaoZhang, KejiaLi, YupingZhang, KuangLi, YuqiZhang, KunLi, YuwenZhang, LameiLi, YuxingZhang, Lei (Nanjing Normal University, China)Li, ZengkeZhang, Lei (Zhejiang University, China)Li, ZhanZhang, LifuLi, ZhaohuiZhang, LihaiLi, ZhengZhang, LijunLi, Zheng (Eddie)Zhang, LiqiangLi, ZhenglinZhang, LiyanLi, ZhengweiZhang, LuLi, ZhenhuaZhang, MingshaoLi, ZhenqingZhang, MingzheLi, ZhiZhang, PeiyunLi, ZhigangZhang, PinjiaLi, ZhihuaZhang, QichunLi, ZhijieZhang, QieshiLi, ZhikangZhang, QijinLi, ZhiqiangZhang, QingLi, ZhixiongZhang, QixingLi, Zhongju (Hugh)Zhang, Quan (China)Li, ZidongZhang, Quan (USA)Li, ZishenZhang, QunLi, ZongtaoZhang, RongLi, ZuohuaZhang, RuiLian, Kuang-YowZhang, RunLiang, Chiu-kuoZhang, ShaohuiLiang, DongZhang, ShaojieLiang, FuyouZhang, ShaolinLiang, Hai NingZhang, ShenLiang, HuaweiZhang, ShengLiang, JianZhang, ShengkaiLiang, JinxingZhang, ShimingLiang, JiuzhenZhang, ShuaiLiang, Jun-RuiZhang, ShulingLiang, RongguangZhang, ShunpingLiang, XihuiZhang, ShunxiangLiang, ZhifangZhang, ShuqingLiang, ZhikaiZhang, SonglinLiang, ZhiqiangZhang, SuLiang, ZiluZhang, Sung-UkLiao, XinqinZhang, TaoLiao, YabinZhang, TianshengLiao, Ying-ChihZhang, TieLiarokapis, FotisZhang, TingtingLiaw, Jiun-JianZhang, TongLiberati, DiegoZhang, WeiLiberatore, NicolaZhang, Wei-QiangLibertino, SebaniaZhang, WeiwenLibra, MartinZhang, WeixingLica, SeptimiuZhang, WenLiccardo, AnnalisaZhang, WenboLicitra, GaetanoZhang, WenjuanLiebermeister, LarsZhang, Wenjun (Chris)Lieberwirth, HolgerZhang, WentaoLiesenberg, VeraldoZhang, WenzengLigat, GaetanZhang, WuxiongLiivik, ElizavetaZhang, XiandouLiKamWa, RobertZhang, XianfengLikhite, RugvedZhang, XiangLikitlersuang, JirapatZhang, XianghuiLiles, Alexandros A.Zhang, XiangyinLilienthal, Achim J.Zhang, XiankuLillo, Paolo DiZhang, Xian-MingLim, GilbertZhang, XiaoLim, JiseokZhang, XiaochenLim, JongwooZhang, XiaofeiLim, Jun LongZhang, XiaolingLim, KihoZhang, XiaomeiLim, MingyuZhang, XiaoqinLim, SeongdongZhang, XiaoshengLim, Si-HyungZhang, XiaosongLim, SolZhang, XiaotongLim, Yee YanZhang, Xiao-YuLima E Silva, Sandro Metrevelle MarcondesZhang, XiaoyueLima, FredericoZhang, XinjieLima, José LuisZhang, XinMingLima, Jose Luiz SousaZhang, Xin-xiangLimiti, ErnestoZhang, XinxinLimniotis, KonstantinosZhang, XuLimongiello, MarcoZhang, XuanjunLin, BeiyuZhang, XudongLin, BorongZhang, XueboLin, Bor-ShingZhang, XueZinLin, Bor-ShiunZhang, XuguangLin, Bor-ShyhZhang, Xu-HuiLin, Cheng-HungZhang, XunLin, Cheng-TeZhang, YahuiLin, ChengyouZhang, Ya-nanLin, Che-WeiZhang, YanshunLin, Chih JerZhang, YanxinLin, Chih-HongZhang, YaoLin, Chih-LangZhang, Ye-WenLin, Chih-LungZhang, YiLin, Chih-MingZhang, YichengLin, Chih-PingZhang, YinLin, Chih-TingZhang, YinshengLin, Chin-FengZhang, YiyiLin, ChingShunZhang, YongLin, Chi-YiZhang, YongboLin, Chi-YingZhang, YonghongLin, Chung-YinZhang, YouminLin, Chun-LingZhang, YouwenLin, Daw-TungZhang, YuLin, Der-YuhZhang, YuanzhiLin, Feng-ChengZhang, YudongLin, FuhongZhang, YuetingLin, Gong-RuZhang, YufeiLin, GuoqingZhang, YunLin, Han-YuZhang, YunfeiLin, HongZhang, YunshengLin, Hong-DarZhang, YutingLin, Hsueh-chunZhang, ZenghuiLin, Huei-YungZhang, ZexuLin, HungyenZhang, ZhaoLin, Hung-YinZhang, ZhaoweiLin, JianZhang, ZhenLin, JianminZhang, ZhengLin, JiaruiZhang, ZhenjiangLin, JuZhang, ZhiguoLin, Jun (China)Zhang, ZhirongLin, Jun (Singapore)Zhang, ZhishengLin, LinZhang, ZhiyangLin, MengZhang, ZhonghuaLin, RuiZhang, ZhouLin, Shu-YenZhang, ZhuoyongLin, TingtingZhang, ZonghuaLin, Tsung-HungZhang, ZuxingLin, Tzu-EnZhangjie, LiuLin, Tzung-HanZhao, ChaofangLin, WeiZhao, ChaoyingLin, WeiguoZhao, ChenLin, Wei-TingZhao, ChunhuiLin, WenpengZhao, DezunLin, Xian-QiZhao, Gang (Shanxi University, China)Lin, YangZhao, Gang (University of Science and Technology of China, China)Lin, Yang-weiZhao, GejianLin, Yi-BingZhao, HaiyanLin, Yu KueiZhao, HongLin, YuankunZhao, HongyuLin, Yuan-PinZhao, HubinLin, Yue-DerZhao, HuiLin, Yu-HaoZhao, HuichanLin, Yu-ShengZhao, JiandongLin, ZhibinZhao, JiangLin, ZhimiaoZhao, JianwenLin, ZihanZhao, JieLinares, Luis JimenezZhao, JingzhouLinares-Barranco, AlejandroZhao, JinlingLinck, RolandZhao, JinqiLindeberg, TonyZhao, JinsongLinden, MariaZhao, JunxuanLindsay, AngusZhao, KaiqiLing, FengZhao, KegangLing, Jeen-MinZhao, LingliLing, Maurice H.T.Zhao, MeiyuLing, SteveZhao, MengtingLinhares, JoãoZhao, MinghaoLinker, RaphaelZhao, MingminLinnartz, Jean-PaulZhao, NanLino, PaoloZhao, NingLins, Raphael DueireZhao, QingchunLins, Vanessa De Freitas CunhaZhao, QinpeiLinstead, ErikZhao, Raymond K.Linzon, YoavZhao, RuiLioe, De XingZhao, ShuaiLiou, MichelleZhao, ShuanfengLipecki, TomaszZhao, SichengLipovac, AdrianaZhao, SihaoLipovac, VlatkoZhao, TiebiaoLipovský, PavolZhao, WeiLipuš, BogdanZhao, WenbingLirn, Ted T. C.Zhao, XuefengLisak, DanielZhao, YangLisboa, Adriano ChavesZhao, YiLisiński, PrzemysławZhao, YingLisjak, DragutinZhao, YongjunLiska, JindrichZhao, YongqiangLisnevskaya, InnaZhao, YongshengLisowiec-Wachnicka, JolantaZhao, YubinLisowski, JózefZhao, YulongLissorgues, GaelleZhao, ZhanListanti, MarcoZhao, ZhiweiLitao, HanZhao, ZhongxiangLitke, AntoniosZhao, ZuominLittke, KimZhaohui, JiangLitwin, DariuszZhen, DongLiu, AnfengZhen, ZhenLiu, BeijiaZheng, BoLiu, BinxinZheng, ChengshiLiu, Bo (Beijing University of Technology, China)Zheng, DonghaiLiu, Bo (Canada)Zheng, GuangchaoLiu, Bo (Ministry of Environmental Protection, China)Zheng, GuoanLiu, Bo (USA)Zheng, GuoqiangLiu, ChangZheng, Hai-TaoLiu, ChangjieZheng, HongYuLiu, ChangqingZheng, HuadanLiu, ChangrongZheng, HuarongLiu, ChaoZheng, Jia-ChunLiu, ChaoranZheng, JunLiu, ChaoxingZheng, LiangLiu, ChengqingZheng, LihongLiu, ChengyuZheng, LimeiLiu, Chia-HuiZheng, LingxiangLiu, Chien-ShengZheng, QiyeLiu, ChuanjunZheng, ShuLiu, Chung-ChiunZheng, ShuanghaoLiu, ChunweiZheng, Tong-Xingliu, DatongZheng, Wei-LongLiu, DongZheng, XianweiLiu, EnjieZheng, XiaochenLiu, Fang (Anhui University, China)Zheng, XingmingLiu, Fang (Hunan University, China)Zheng, YangongLiu, FangmingZheng, YifengLiu, Fei (Canada)Zheng, YihaoLiu, Fei (China)Zheng, YuhuiLiu, GuigenZheng, ZhenrongLiu, GuixiongZheng, ZhubinLiu, GuoliangZherdev, Anatoly V.Liu, HaijunZherebtsov, EvgenyLiu, HailongZhironkin, SergeyLiu, HaiwenZhiyuan, MaLiu, HaiyunZhmud, VadimLiu, HaojieZhong, BotaoLiu, HongchaoZhong, DexingLiu, HonghaiZhong, JiahaoLiu, HongshunZhong, JingangLiu, HongweiZhong, RuofeiLiu, HongyunZhong, ShuncongLiu, HuafengZhong, YuanhongLiu, HuajianZhongyu, WangLiu, HuajunZhou, AoLiu, HuanZhou, BrianLiu, HuiZhou, ChangluLiu, Hung-HuanZhou, ChaoLiu, JialunZhou, ChenLiu, JiangZhou, ChengweiLiu, JianhuaZhou, CimingLiu, JianqiZhou, DingfuLiu, JiaruiZhou, FengLiu, JieZhou, FengfengLiu, JiechaoZhou, GenghengLiu, JieminZhou, GongjianLiu, JinfuZhou, GuoqingLiu, JingZhou, HaiboLiu, JingfeiZhou, HongliangLiu, Jing-SinZhou, HuachunLiu, JuewenZhou, HuapengLiu, JunZhou, JiLiu, KaiZhou, JianLiu, Kai-ChunZhou, JianhuaLiu, KaizhouZhou, JinghaiLiu, KangliZhou, JunLiu, Keng-HaoZhou, KaiLiu, Keng-KuZhou, KaiqingLiu, KunZhou, LelaiLiu, LanjunZhou, LianjieLiu, LeiZhou, LinrenLiu, LiZhou, MengchuLiu, LiangguiZhou, MingchuanLiu, LianqingZhou, MingdaLiu, LianxiZhou, NanLiu, LinboZhou, NanrunLiu, LingjieZhou, ShangboLiu, LinshanZhou, ShijieLiu, LongZhou, SusanLiu, LongjuZhou, TanLiu, MeilingZhou, TunheLiu, Meng-Kun (Jason)Zhou, WeixunLiu, MenglongZhou, WenLiu, MengmengZhou, WenjunLiu, MinZhou, WenyaLiu, Ming (China)Zhou, WuLiu, Ming (USA)Zhou, Xiao DongLiu, MinghuaZhou, XiaoboLiu, MingyongZhou, XiaohongLiu, MinjingZhou, XinLiu, MinliangZhou, XuLiu, Peng (China)Zhou, Yang (UK)Liu, Peng (USA)Zhou, Yang (USA)Liu, PengchengZhou, YongjinLiu, PengfeiZhou, YuLiu, PingweiZhou, YuanLiu, QiZhou, YubinLiu, QiangZhou, YufengLiu, QiongZhou, YunlaiLiu, QuanhuaZhou, ZebingLiu, RanZhou, ZeboLiu, RongZhou, ZeyangLiu, ShangzhongZhou, ZhengganLiu, ShaoqingZhou, ZhenyuLiu, Sheng-ChiZhou, ZhidongLiu, ShenghengZhou, ZhiqiangLiu, ShijunZhou, ZhuhuangLiu, Shing-HongZhou, ZimuLiu, ShuaiqiZhu, AijunLiu, ShuangZhu, BangyanLiu, SongtaoZhu, BenpengLiu, SongzuoZhu, BowenLiu, TaihongZhu, ChiLiu, TaoZhu, ChunshengLiu, TengZhu, CongxuLiu, TongZhu, Eric Y.Liu, Tzong-shiZhu, FengLiu, WankeZhu, GuohunLiu, WeiZhu, HangLiu, WeiboZhu, HaoLiu, WeiluZhu, LidaLiu, Wei-MinZhu, LipingLiu, Wei-RongZhu, LiqiangLiu, WeitingZhu, LiujunLiu, WeiweiZhu, MengshiLiu, Wen-FungZhu, MinminLiu, XianghongZhu, NiLiu, XianglinZhu, QiuguoLiu, XiaoboZhu, QiumingLiu, XiaofanZhu, WenbinLiu, XiaofengZhu, XiLiu, XiaomingZhu, XiaoliangLiu, XiaoweiZhu, XiaozhangLiu, XiaoyuZhu, XiufangLiu, XinZhu, XuanLiu, XinanZhu, Xu-DongLiu, XingjianZhu, YaguangLiu, XiongyingZhu, YongLiu, XiupingZhu, YuchuanLiu, YadongZhu, ZhigangLiu, YahuaZhu, ZijieLiu, YanZhu, ZuqingLiu, Yan-AnZhuang, ChijieLiu, YangZhuang, HanqiLiu, YeZhuang, XinLiu, YiminZhuang, YanLiu, YingZhuo, LiLiu, YingtaoZhuo, YueLiu, YingxiangZi, BinLiu, YitaoZia, Muhammad Yousuf IrfanLiu, YizhiZiakopoulos, ApostolosLiu, YongŽibrat, UrošLiu, Yong-JinZidanšek, AleksanderLiu, YuZielinski, SlawomirLiu, YuelingZielinski, ZbigniewLiu, YufeiZiembik, ZbigniewLiu, YuHaoZigan, LarsLiu, YukangZigel, YanivLiu, ZhaobinZikidis, Konstantinos C.Liu, ZheliZikria, Yousaf BinLiu, ZhengZilberman, NadezhdaLiu, ZhenglinZillger, TinoLiu, ZhenguangZima, BeataLiu, ZhengxiongZimmerman, WilliamLiu, ZhengyongZimnoch Dos Santos, João HenriqueLiu, ZhentaoZimon, GrzegorzLiu, ZhenzhongZinno, RaffaeleLiu, ZhifuZiock, KlausLiu, ZhiliangZiogou, ChrysovalantouLiu, ZhipingZiółkowski, CezaryLiu, ZhiyongZiolkowski, PatrykLiu, ZhuofuZiolkowski, PawełLiuzzi, GiulianoZiółkowski, RobertLivens, StefanZissis, GeorgesLivieris, Ioannis E.Zivcak, MarekLivreri, PatriziaŽivković, IgorLizama-Pérez, Luis AdrianZiyatdinova, GuzelLizcano Casas, DavidZizzo, GaetanoLizzi, LeonardoZlatev, ZlatinLlamas Bello, CésarZmarzły, PawełLlaria, AlvaroŽmuk, BerislavLlata, Marta RuizZografopoulos, DimitriosLlera, MiguelZohrabi, NasibehLlobet, EduardŻokowski, MariuszLlopis Castelló, DavidZola, EnricaLlopis-Lorente, AntoniZolotoukhina, TatianaLloret, JaimeZong, ChengLloyd, HuwZong, QunLo Iacono, FrancescoZong, XianzhengLo, Kin HingZorbas, DimitriosLo, On-Yee AmyZormpas, DimitriosLo, Shi-WeiZorzi, MattiaLo, Wai LunZotti, AldobenedettoLo, Yi-KaiZou, FangxinLoayssa, AlaynZou, LiangLobato, Daniel Ferreira MoreiraZou, ShihongLobato-Morales, HumbertoZou, TingLobaton, EdgarZou, WulinLobo, AndersonZou, XiangjunLobo-Castañón, María JesúsZou, XiaoboLoc, Ho HuuZou, XinLoddo, AndreaZou, XudongLoeff, LuukZou, YajieLoget, GabrielZou, YongpanLogozzo, SilviaZou, ZhiqiangLogsdon, SallyZouhar, AlexanderLoh, PaulZribi, MehrezLohan, Elena-SimonaZrinjski, MladenLok Woo, WaiZu, Theresah Nom KorbiehLokhorst, KeesZu, YouliLokshina, IzabellaZubizarreta, AsierLolli, SimoneZubrzycki, JarosławLombardi, AngelaZucali, MaddalenaLombardi, FedericoZucco, MassimoLombardo, LucaZuccon, PaoloLombardo, TeresaZude, ManuelaLomillos Pérez, Juan ManuelZuffo, MichelaLomonaco, TommasoZügner, RolandLomond, KarenŽukowski, Paweł V.Loncarski, JelenaŽulec, MirnaLonetti, FrancescaZuluaga, Maria A.Long, ChengZupan, DejanLong, TengfeiZuppella, PaolaLongo, RiccardoŽuraulis, VidasLongo, SavinoZurauskiene, NerijaLongo, Umile GiuseppeZurbuchen, AdrianLongoni, LauraZurita, GroverLongoria-Gandara, OmarZvánovec, StanislavLontio Fomekong, RoussinZweiri, YahyaLontos, AntoniosZwierko, TeresaLopes Rosa, RenataZych, MarcinLopes, FernandoŻyczkowski, MarekLopes, Sérgio I.Żygierewicz, JarosławLopez Anton, RicardoZygmunt, MarekLópez Espí, Pablo LuisZymelka, DanielLópez Granado, Otoniel MarioZywica, GrzegorzLopez Neri, Emmanuel


